# Mass Difference Matching Unfolds Hidden Molecular Structures of Dissolved Organic Matter

**DOI:** 10.1021/acs.est.2c01332

**Published:** 2022-07-14

**Authors:** Carsten Simon, Kai Dührkop, Daniel Petras, Vanessa-Nina Roth, Sebastian Böcker, Pieter C. Dorrestein, Gerd Gleixner

**Affiliations:** † Molecular Biogeochemistry, Department of Biogeochemical Processes, Max Planck Institute for Biogeochemistry, Hans-Knöll-Straße 10, 07745 Jena, Germany; ‡ Chair for Bioinformatics, Faculty of Mathematics and Computer Science, Friedrich Schiller University Jena, Ernst-Abbe-Platz 2, 07743 Jena, Germany; § Collaborative Mass Spectrometry Innovation Center, Skaggs School of Pharmacy and Pharmaceutical Sciences, University of California San Diego, 9500 Gilman Drive, MC 0657, La Jolla, California 92093-0657, United States of America; ∥ CMFI Cluster of Excellence, Interfaculty Institute of Microbiology and Medicine, University of Tübingen, Auf der Morgenstelle 24, 72076 Tübingen, Germany

**Keywords:** natural organic matter, NOM, DI-ESI-MS/MS, FTMS, Orbitrap, tandem mass spectrometry, MS/MS, deconvolution

## Abstract

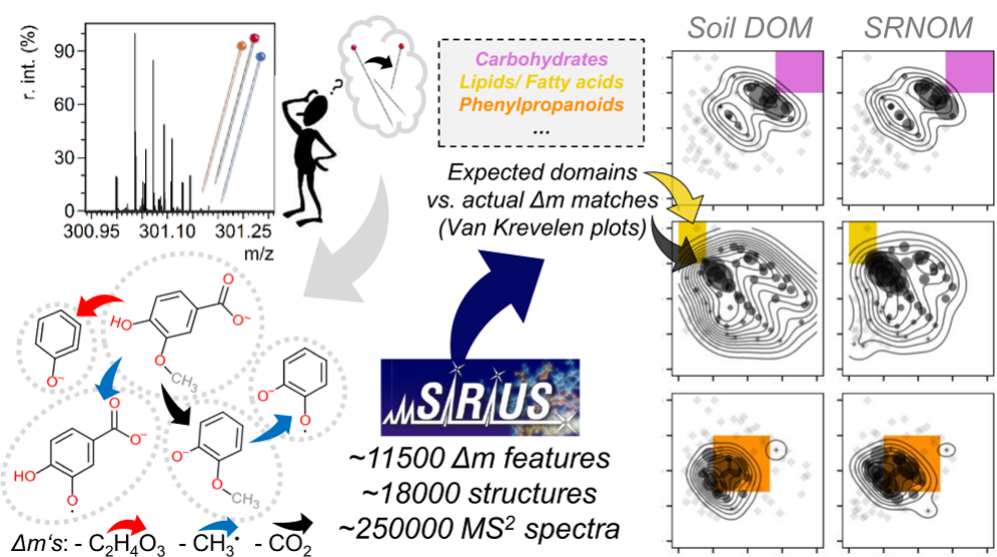

Ultrahigh-resolution Fourier transform mass spectrometry (FTMS) has revealed unprecedented details of natural complex mixtures such as dissolved organic matter (DOM) on a molecular formula level, but we lack approaches to access the underlying structural complexity. We here explore the hypothesis that every DOM precursor ion is potentially linked with all emerging product ions in FTMS^2^ experiments. The resulting mass difference (Δ*m*) matrix is deconvoluted to isolate individual precursor ion Δ*m* profiles and matched with structural information, which was derived from 42 Δ*m* features from 14 in-house reference compounds and a global set of 11 477 Δ*m* features with assigned structure specificities, using a dataset of ∼18 000 unique structures. We show that Δ*m* matching is highly sensitive in predicting potential precursor ion identities in terms of molecular and structural composition. Additionally, the approach identified unresolved precursor ions and missing elements in molecular formula annotation (P, Cl, F). Our study provides first results on how Δ*m* matching refines structural annotations in van Krevelen space but simultaneously demonstrates the wide overlap between potential structural classes. We show that this effect is likely driven by chemodiversity and offers an explanation for the observed ubiquitous presence of molecules in the center of the van Krevelen space. Our promising first results suggest that Δ*m* matching can both unfold the structural information encrypted in DOM and assess the quality of FTMS-derived molecular formulas of complex mixtures in general.

## Abstract 2

We present an approach to deconvolute and explore the structural composition of co-fragmented mixtures of organic molecules in environmental media.

## 1 Introduction

Complex mixtures are key study objects in environmental and industrial applications, but their analysis remains challenging.^1−4^ One of the most complex mixtures in natural ecosystems is dissolved organic matter (DOM).^5,6^ DOM is a central intermediate of ecosystem metabolism and mirrors molecular imprints of interactions with its abiotic and biotic environment,^7−9^ which form the basis for processes such as carbon sequestration and nutrient recycling.^10,11^ Despite significant advances in ultrahigh-resolution mass spectrometry (FTMS)^2,4^ and nuclear magnetic resonance spectroscopy,^12^ scientists still struggle to decode this information on the molecular level,^13−17^ and novel approaches to identify distinct structures are required to translate molecular-level information into improved process understanding.

Open and living systems promote the formation of ultracomplex mixtures of thousands to millions of individual constituents^18,19^ that mirror large environmental gradients.^20−22^ As a consequence, DOM poses significant challenges to separation, isolation, and structure elucidation. Direct infusion (DI) FTMS techniques have become indispensable tools for the molecular-level analysis of DOM as they reveal unprecedented details of molecular formulas using the exact mass (MS^1^ data, *m*/*z*) even without prior separation.^23^ However, FTMS techniques are selective and do not resolve all structural detail observed at the exact mass in DOM, as the presence of isobars and isomers hinders the identification of particular structures from these molecular formulas.^19,23−25^ Additionally, current structural databases cover only a small fraction of molecular formulas encountered and typically lead to annotation rates <5%.^18,26,27^

One way to obtain structure information on isomers and isobars is through collision-induced dissociation (CID; MS^2^, or multistage MS^n^).^27−29^ The relatively wide isolation window (∼1 Da) of mass filters applied for precursor ion selection commonly hinders the isolation and subsequent fragmentation of single exact masses, leading to mixed “chimeric” MS^2^ spectra of co-fragmented precursor ions.^30^ Even though some authors achieved isolation of single masses or improved description of chimeric tandem MS data, fragmentation patterns were found to be universal across DOM samples.^18,19,31−35^ Most of these studies, however, focused on the major product ion peaks (fragments), which usually make up only 60–70% of the total product ion abundance, and thus disregarded many low-abundance signals that may be more suitable to detecting structural differences.^19,31^

The major product ions encountered in tandem mass spectra of DOM relate to sequential neutral losses of common small building blocks, mainly CO_2_, H_2_O, or CO units.^14,33^ A mass difference between a precursor and a product ion in an MS^2^ spectrum is herein called “delta mass” and referred to as Δ*m* (plural Δ*m*’s). Common Δ*m*’s such as CO_2_ or H_2_O are deemed nonindicative for the identification of structural units.^18,28,31,33,36^ In contrast, other studies found recurring low-*m*/*z* product ions (e.g., at *m*/*z* 95, 97, 109, 111, 123, 125, 137, 139, 151, and 153) that were interpreted as a limited set of core structural units substituted with a set of functional groups, yet in different amounts and configurational types that would lead to highly diverse mixtures.^37−44^ From a stochastic standpoint, the occurrence of common neutral losses may not be surprising; many structures contain hydroxyl groups that could yield H_2_O losses, and CO_2_ could originate from ubiquitous carboxyl groups.^45^ In contrast, the occurrence of two molecules sharing a larger substructure would be less probable and less easily detected as a major peak. Signatures of DOM’s structural diversity could thus prevail in the high number of low-abundance fragments usually detected below *m*/*z* 200–300, as opposed to the higher abundance of fragments connected to losses of CO_2_ or H_2_O. Given the large number of estimated isomers and isobars underlying usual DOM data,^18,19,31,32,39,45−48^ we here build upon the hypothesis that every co-fragmented precursor ion potentially contributes to every emerging product ion signal. We interpret the resulting chimeric MS^2^ data as a structural fingerprint that can be deconvoluted to obtain individual precursor ion Δ*m* matching profiles. The analysis of Δ*m*’s that link precursor and product ions is independent of the masses of the unknown precursor ions and known reference compounds in databases of annotated Δ*m* features, and therefore does not rely on indicative product ions (fragments) alone. Although this approach sacrifices the identification of true knowns, it allows for the identification of potential structural analogues via indicative Δ*m*’s and may be especially suited when annotation rates are as low as in the case of DOM, i.e., when most compounds are unknown.^18,26,27^

Despite the unknown identity of most of the molecules present in DOM, its potential sources can be constrained reasonably well. Plants produce most of the organic matter that sustains food webs in natural ecosystems. Plant metabolites such as polyphenols thus represent a major source of DOM. Therefore, an early decomposition phase likely exists when the imprint of soluble/solubilized plant metabolites is still detectable by MS^2^ experiments using current FTMS technology: Lignin-related compounds show indicative methoxyl/methyl radical losses,^18,49,50^ glycosides indicate a sugar loss,^51,52^ and hydrolyzable tannins may lose galloyl units.^52^ Mass differences related to atoms such as N, S, P, Cl, Br, I, and F could also help to identify unknown organic nutrient species or disinfection byproducts, thereby widening the applicability of the approach.^1,53^ Finally, indicative Δ*m* fingerprints could provide constraints to putative compound group annotations derived from molecular formula data alone (van Krevelen diagrams) or allow for a more precise annotation.^54−56^

We hypothesized that DOM from swamps and topsoil, in close contact with plant inputs and active microbial communities, would reflect recognizable plant-related source imprints that can be revealed by tandem mass spectrometry. Specifically, we explored links between precursor ion Δ*m* matching profiles and precursor ion characteristics such as nominal mass, mass defect, initial ion abundance, fragmentation sensitivity, oxygen-to hydrogen ratio (O/C), heteroatom content, and structure suggestions. These properties are in part predictable from the assigned molecular formula, and thus allow for an evaluation of the approach (“proof of concept”) while also revealing potential nonassigned molecules (e.g., P-, Cl-, Br-, I-, and F-containing molecular formulas). Finally, we hypothesized that indicative Δ*m* features of plant phenols, e.g., lignin- and tannin-related losses, would match their yet unknown structural analogues in DOM and that these patterns would reflect commonly applied “structural domain” distributions.^55,57,58^

## 2 Experimental Section

A detailed experimental procedure is provided in the Supporting Information of this article (Note S-1). In short, we chose 14 aromatic reference compounds as representative plant metabolites (Figure S-1 and Table S-1) and a forest topsoil pore water isolate^59^ and Suwannee River Natural Organic Matter (SRNOM)^60^ as exemplary DOM samples. All reference and sample solutions were directly infused into the ESI (electrospray) source of an Orbitrap Elite (Thermo Fisher Scientific, Bremen) at negative ionization mode (Table S-2) and fragmented by collision-induced dissociation (CID, MS^2^). We chose four nominal masses within the mass range typically observed in terrestrial DOM samples (*m*/*z* 200–500) for fragmentation (*m*/*z* 241, 301, 361, and 417, herein referred to as isolated precursor ion mixtures, “IPIMs”) to test the approach.^61^ Soil DOM was analyzed at three normalized collision energy (NCE) levels (15, 20, and 25%). MS^3^ spectra of selected key product ions (aglycons of flavonoids and demethylated dimethoxy-methyl-benzoquinone) were acquired at NCE 20 or 25. After recalibration with known (Table S-3) or predicted product ions (losses of CO_2_, H_2_O, etc.), all major product ions were annotated with a molecular formula in reference compounds (Figure S-2, Tables S-4, and Table S-5) and DOM. Formula annotation was conducted with a Matlab routine recently incorporated into an open FTMS data processing pipeline.^62^

For MS^2^ data analysis, we generated Δ*m matrices* of every pairwise combination of precursor and product ions (“Δ*m* fingerprints”). Every value in this matrix is referred to as a Δ*m feature* or Δ*m*. We compared the unknown Δ*m* features in DOM to three lists of known Δ*m* features:

(a) 54 Δ*m* features ubiquitously found in DOM (Table S-6),
(b) 55 Δ*m* features from the set of 14 reference compounds (Table S-7), and
(c) 11477 Δ*m* features from a negative ESI MS^2^ library with 249 916 reference spectra of 17 994 unique molecular structures annotated by SIRIUS^63^ (Figure S-3; list in supporting datasets). Reference spectra were collected from GNPS, MassBank, MoNA, and NIST.^64,65^

The detection of a known Δ*m* feature in DOM is herein called “Δ*m matching*” and detected Δ*m* features are called Δ*m matches*. Matching was conducted at a mass tolerance of ± 0.0002 Da (2 ppm at 200 Da). The array of Δ*m* matches of a single precursor ion is called the Δ*m matching profile*, and all precursor ion profiles of an IPIM form the subset of matched Δ*m*’s of the Δ*m* matrix introduced above. The decomposition of the MS^2^ spectrum into a Δ*m* matrix and therefore, individual Δ*m* matching profiles is what we define as the *deconvolution* step in this study. Δ*m*’s of lists (a) and (b) showed some overlap and were largely part of list (c) as well. The specificity of any Δ*m* feature in list (c) was checked by their association to compound classes as defined by ClassyFire.^66^ The top 15 significantly associated classes were then obtained for each Δ*m* feature in list (c) and included in analyses using the reference-compound-derived list (list b) as well.

We assessed the probability of false-positive matches and accounted for the number of elements in the formula, ion abundance, and measures of fragmentation sensitivity to validate our approach. The matching data were combined for each NCE level and transformed into a binary format. We classified Δ*m* matching profiles of DOM precursor ions and reference compounds of lists (b) and (c) by two-way hierarchical clustering using Ward’s method and Euclidean distance, as well as Principal Components Analysis (PCA) in PAST (v3.10) for list b.^67^ We visualized numbers of individual Δ*m* matches and Δ*m* cluster matches in van Krevelen space for all lists. We chose the structural domains reprinted in the 2014 review by Minor et al. for reference because this represents the general level of detail and type of classes distinguished in recent DOM studies (Figure S-4).^57,58,68−70^ In two separate analyses, formulas were also classified with a more general and a data-based van Krevelen scheme besides the reference one.^58,71^

Finally, we assessed the agreement between structures predicted by Δ*m* matching and those suggested in natural product structural databases. We combined structure suggestions from different databases, including Dictionary of Natural Products,^72^ KNApSAcK,^73^ Metacyc,^74^ KEGG,^75^ and HMDB,^76^ as well as their expanded in silico annotations based on predicted enzymatic transformations in the MINEs database.^77^ Although the MINEs database covers 198 generalized chemical reaction rules it may not include all potential environmental reactions because those are not solely driven by enzymes. The InChi-Key of structures was used to exclude stereoisomers and classify suggested structures into compound classes by ClassyFire.^66^

## 3 Results and Discussion

### 3.1 Tandem MS Fragmentation of Reference Compounds and Construction of Δ*m* Lists

The 14 reference compounds (Figures S-1, S-2, and S-4) yielded 42 unique Δ*m* features (i.e., not covered in list a, Table S-6) but also eight that were described in DOM. These eight Δ*m* features (namely: H_2_O, 18.0106; CO, 27.9949; C_2_H_4_, 28.0313; C_2_H_2_O, 42.0106; CO_2_, 43.9898; CH_2_O_3_, 62.0004; C_2_O_3_, 71.9847; and C_3_O_5_, 115.9746) were kept in list (b) to compare DOM and reference compounds (Table S-7). Besides precursor ion formulas #2 (Hydroxy-cinnamic acid, or p-coumaric acid; C_9_H_8_O_3_, 164.0473), #3 (Gallic acid; C_7_H_6_O_5_, 170.0215), and #5 (m-Guaiacol; C_7_H_8_O_2_, 124.0524), which were found among the 42 Δ*m*’s as potential structural equivalents, five Δ*m*’s of potential substructures likely to be found in DOM were added to list b, namely, precursor ions #1 (Vanillic acid; C_8_H_8_O_4_, 168.04226), #4 (Creosol, C_8_H_10_O_2_, 138.0681), #8 (Ellagic acid; C_14_H_6_O_8_, 302.0063), and #10 (Catechin; C_15_H_14_O_6_, 290.0790), and the neutral aglycon of compounds #12 and #13 (flavonol core of Spiraeoside and Isoquercitin; C_15_H_10_O_7_, 302.0427). More details on reference compound fragmentation are given in the SI (Note S-2).

### 3.2 Fragmentation Behavior of Soil DOM

DOM precursor ions were isolated and fragmented to obtain Δ*m* data via matching (Figure S-5). To find the best collision energy to fragment DOM, we analyzed soil DOM at three NCE levels (15, 20, and 25). All IPIMs showed similar fragmentation properties (Note S-3 and Table S-8). Highest numbers of product ions were found at the highest NCE (Figure S-6). Product ion spectra did not indicate abrupt structural changes upon increasing NCE, showing no separation of isomers/isobars but a continuous increase in fragmentation across all precursor ions. Based on the above results, NCE of 25 was chosen to fragment SRNOM for comparison.

**Figure 1:**
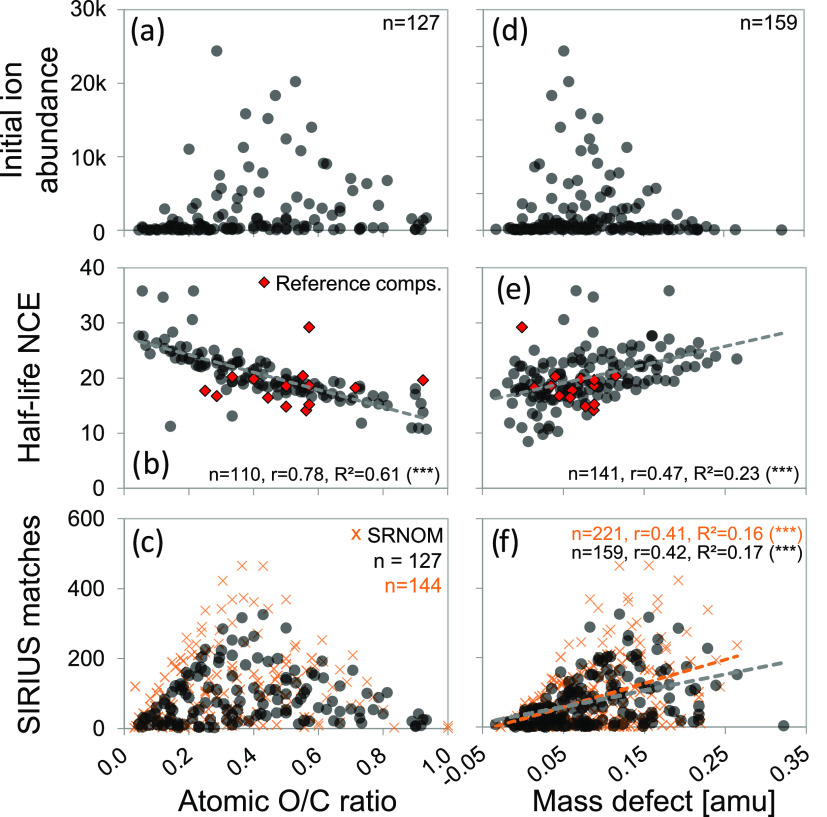
Links between selected DOM precursor ion properties (top panels, initial ion abundance at NCE 0; middle panels, half-life normalized collision energy (NCE) at which ion abundance has dropped by 50%; bottom panels, matches of delta masses (Δ*m*’s) of measured precursor and product ion masses (delta masses, Δ*m*) with a list of 11477 known Δ*m* features from SIRIUS) and each precursor ions’ O/C ratio (a–c) or mass defect (d–f). O/C ratios can only be shown for precursor ions with an annotated molecular formula. Black dots are individual soil DOM formulae. Additional data from reference compounds (red diamonds, see also Figure S-4) and SRNOM (orange crosses) is shown in middle and bottom panels, respectively. Statistical data were derived from linear fits; asterisks (***) denote *p*-value < 0.001.

**Figure 2:**
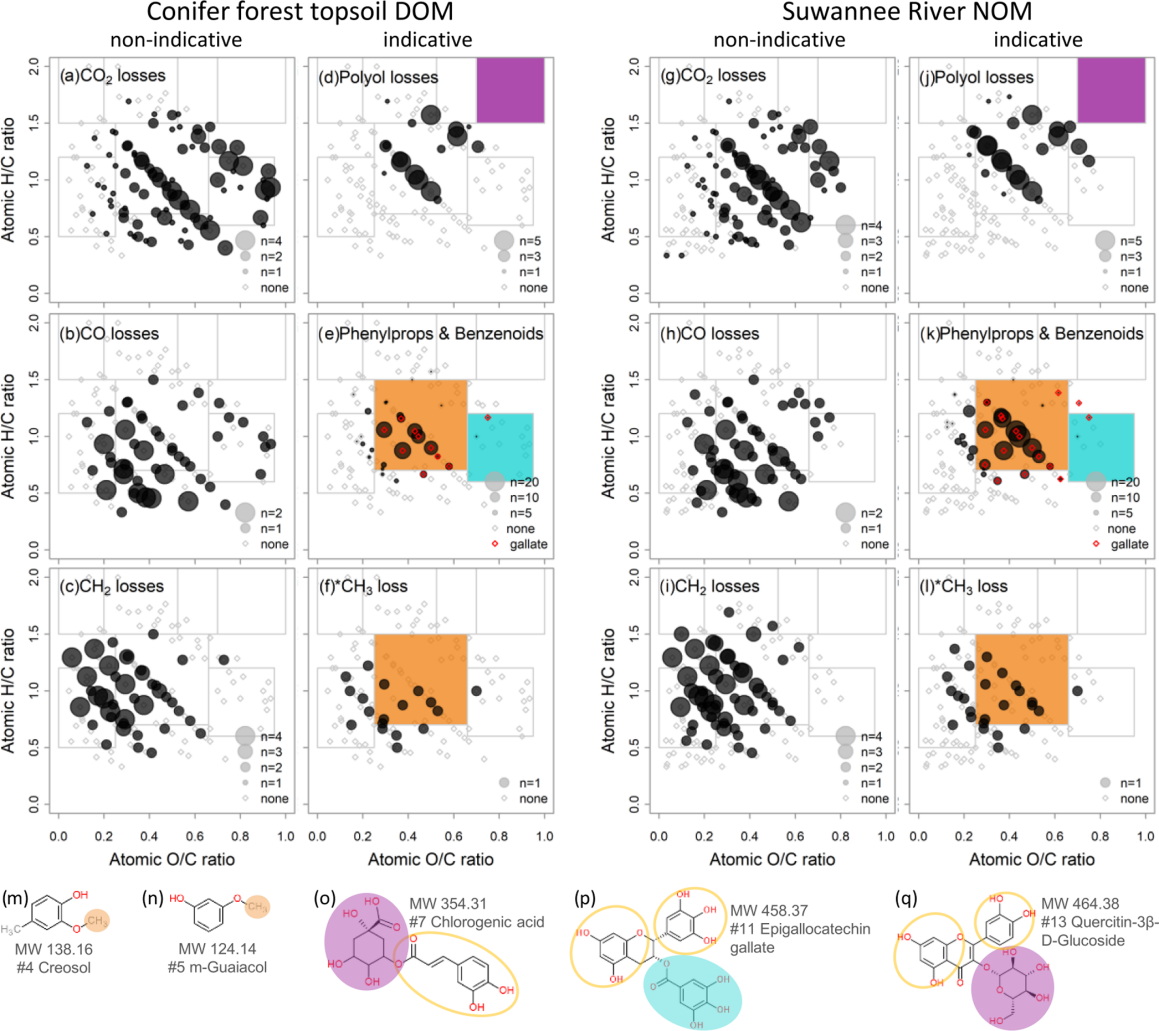
Δ*m* matches visualized in chemical space for soil (porewater) DOM (a–f) and SRNOM (g–l). Exemplary reference compound structures with potential indicative Δ*m* units are shown in bottom panels (m–q). “Phenylprops” refers to the shortened Classyfire class of “Phenylpropanoids and polyketides”. Gray outlined boxes refer to anticipated structural domains (Figure S-4).^64^ (a–l) Precursor ions with an annotated molecular formula by their atomic H/C and O/C ratios (van Krevelen plot; soil DOM, *n* = 127; SRNOM, *n* = 144). Dot size encodes the number of matches to nonindicative (a–c, g–i) vs indicative Δ*m*’s (d–f, j–l); see legends in every plot. Colored boxes in indicative VK plots mark the expected structural region of formulas that would be expected to yield the respective Δ*m*, and colors refer to the structural motifs marked in (m–q). Phenylpropanoid or benzenoid-like (sub-)structures as the ones shown in empty circles (o, p, q) may also contain methyl or methoxy groups (filled orange dots in m, n) that could produce methyl radical losses. Calculations based on Δ*m* data are presented in more detail in Table S-13. Highlighted red open diamonds in (e) and (k) indicate loss of up to three gallic acid equivalents (size not drawn to scale).

**Figure 3:**
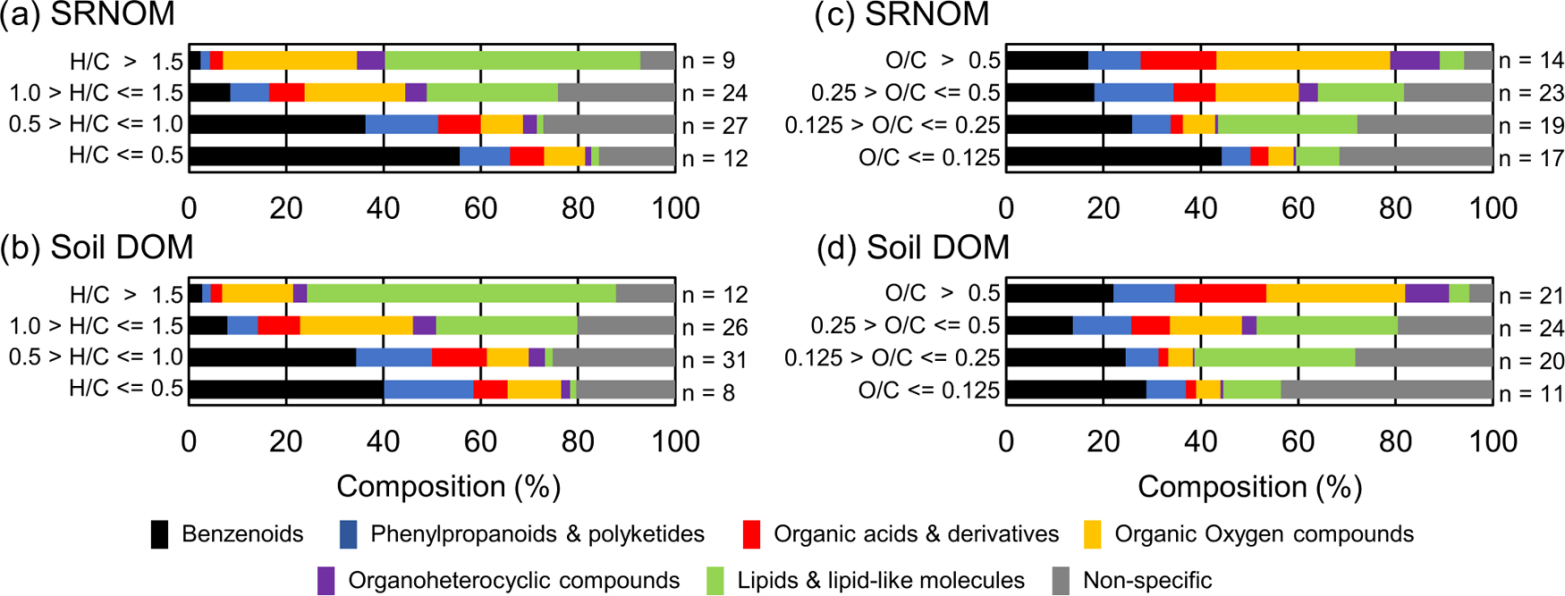
Potential structural composition of exemplary sections in van Krevelen space shown in the H/C (a, b) and O/C (c, d) dimensions. Data from both DOM samples and for CHO precursors are presented. Each bar represents 100% of information derived from matched Δ*m* features (list c) per precursor ion; compositions of individual precursors are aggregated as averages here for visualization (numbers of precursors given behind bar). Examples of individual precursor compositions are shown in the Supporting Information (Figure S-12). The analysis is based on the association of Δ*m* features (list c) with their SIRIUS-annotated molecular (host) structures (details given in Note S-1). Structural classes are shown at the most aggregated class level of the Classyfire Ontology here (compare innermost circle in Figure S-3) but would allow for finer differentiation in future applications.

**Figure 4:**
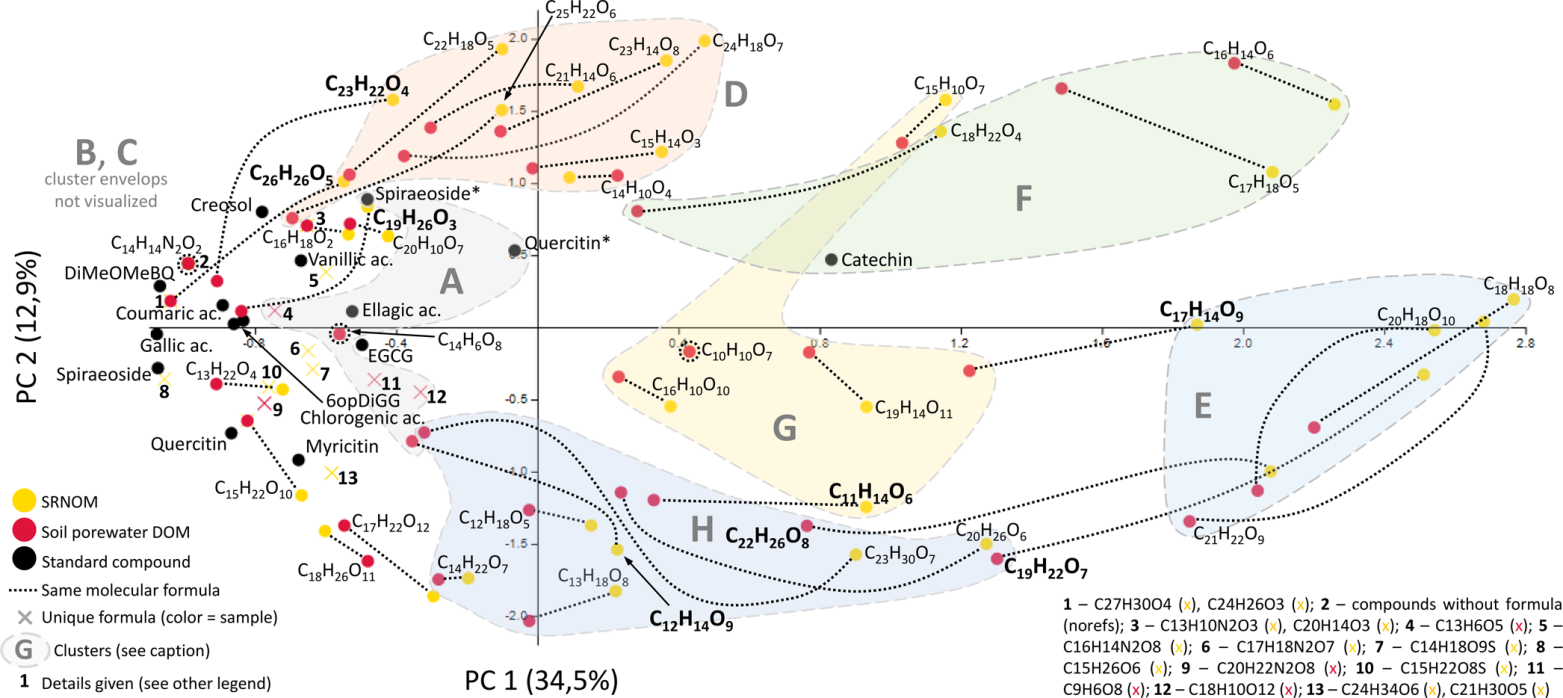
Separation of DOM precursor ions based on Δ*m* matching with list b. Principal component analysis of all precursor ions with more than one match to indicative Δ*m* features of the 14 reference compounds (i.e., Δ*m* features shown in Table S-7 that are not part of Table S-6, see Section 3.1). Colors of dots distinguish precursor ions from both samples and reference compounds (see legend). Precursor ions detected in both samples are connected by dotted black lines. Precursor ion clusters (A–H) are marked by envelopes and letters (compare Tables 1 and S-14). Eight shared precursor ions that switched precursor ion clusters are highlighted by bold molecular formula (C_12_H_14_O_9_, A in soil DOM → H in SRNOM; C_19_H_26_O_3_, B → C; C_26_H_26_O_5_ and C_23_H_22_O_4_, B → D; C_17_H_14_O_9_, G → E; C_19_H_22_O_7_ and C_22_H_26_O_8_, H → E; C_11_H_14_O_6_, H → G).

**Figure 5:**
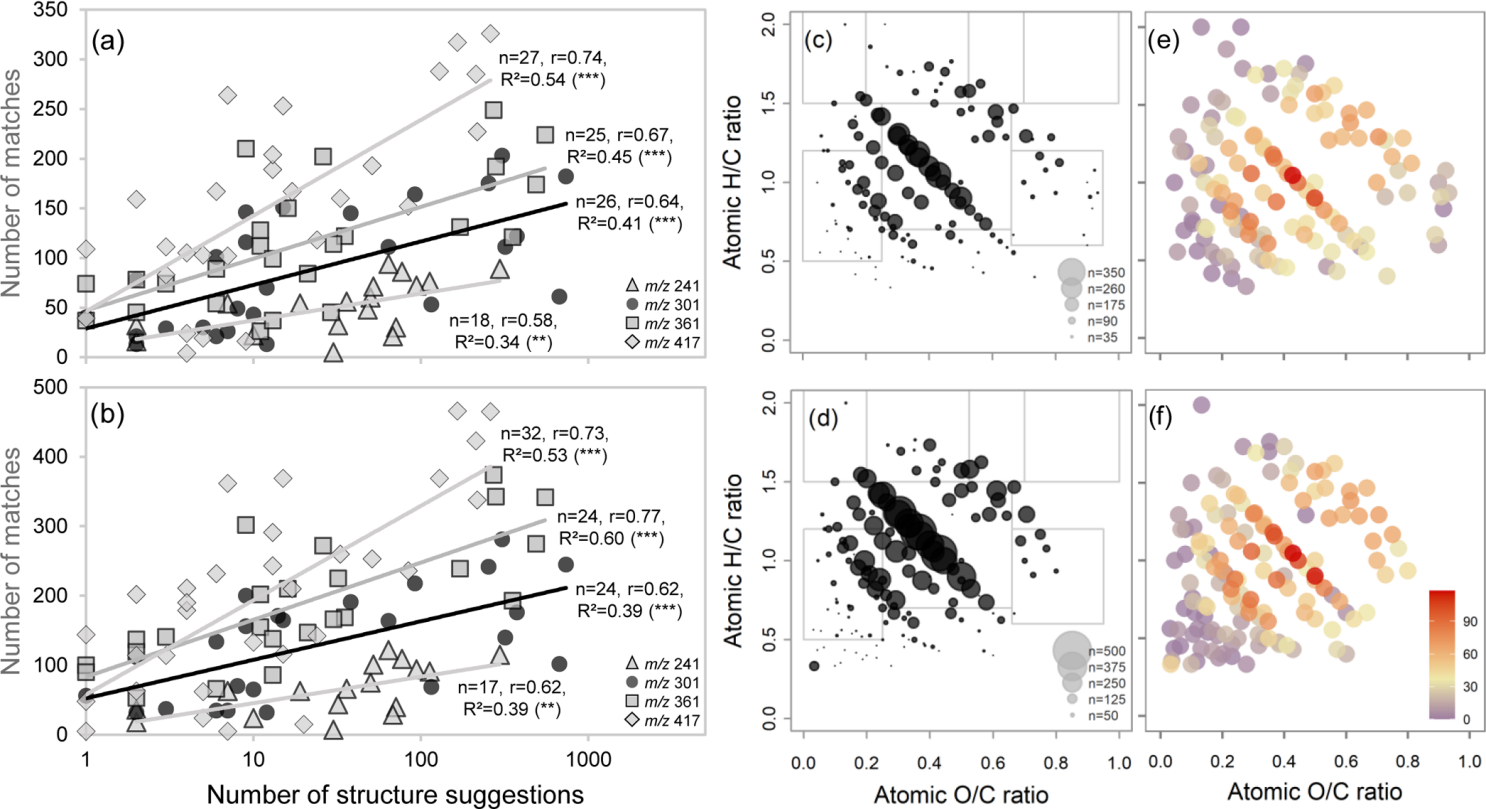
Agreement between chemodiversity estimates based on molecular formula (structure suggestions) and precursor-product ion links (Δ*m* matches). (a, b) Correlations between numbers of SIRIUS Δ*m* matches (list c) vs structure suggestions (note log scale, incl. in silico hits): (a) soil DOM, (b) SRNOM. (c, d) Number of SIRIUS Δ*m* matches in van Krevelen space (scales are similar but legends show different dot sizes): (c) soil DOM, (d) SRNOM; gray boxes refer to structural domains defined in Figure S-4. (e, f) Number of predicted classes per precursor ion based on SIRIUS Δ*m* matches (color scale similar in both panels). Structural classes are associated with SIRIUS-annotated Δ*m* features through correlation analysis of host structures and their Δ*m* features (classification based on Classyfire): (e) soil DOM, (f) SRNOM.

Despite common differences between precursor ion abundance and O/C ratio or mass defect (Figure 1a,d), we found a significant positive link between both metrics and fragmentation sensitivity independent of nominal mass, ranging from half-life NCE (i.e., the NCE level causing 50% decrease in ion abundance) of 10–35 under our instrumental settings (calculated from linear fits). Remarkably, this trend was not observed in reference compounds (Figure 1b,e). Such a discrepancy has been observed also by Zark et al. for the common CO_2_ loss and was interpreted as a result of intrinsic averaging.^31,45^ In contrast, Dit Foque et al. described the potential separation of less complex isomer mixtures by ramped fragmentation.^29^ Bearing the limitation in mind that we only analyzed four IPIMs here, our results support the intrinsic averaging hypothesis and indicate that fragmentation sensitivity may be an additional property shaped by DOM complexity.^18,20,45^ It also supports our assumption of a high number of isomers and isobars “hidden” beneath each precursor ion molecular formula, which also increases the probability to detect meaningful links between precursor and product ions. A minor group of oxygen-poor formulas was nonresponsive (Note S-3). Matching to list c showed no significant relation to O/C ratio but to mass defect (Figure 1c, f). In contrast to mass defect, initial ion abundance showed no link to fragmentation sensitivity but was significantly correlated to higher numbers of Δ*m* matches (*r* = 0.41, *R*^2^ = 0.17, *n* = 157, *p* < 0.001; see also Tables S-9, S-10, S-11, and S-12, and Figure S-7). DOM precursor ions with an average O/C ratio matched more often than low-O/C, fragmentation-resistant precursor ions (Figures 1c and S-8, Note S-3)^18,19,35^ or high-O/C, easily fragmented precursor ions (Figure 1b). These observations together show that fragmentation sensitivity and Δ*m* matching seem to be independent DOM precursor ion properties and that Δ*m* matching could be driven by ion abundance. SRNOM and the soil water sample shared most molecular formulas (*n* = 107; 84% of soil DOM and 74% of SRNOM formulas) and accounted for most of precursor ion abundance at NCE 25 (96.5% and 97.2%, respectively). Despite this high similarity, SRNOM precursor ions showed higher numbers of Δ*m* matches (Figure 1c,f), which could indicate that the same molecular formula is more chemodiverse, i.e., has more underlying structural formulas in SRNOM compared to soil DOM (Section 3.5).

### 3.3 Evaluation of the Δ*m* Matching Approach

We used the matching data of molecular formulas in DOM for a proof-of-concept evaluation of our Δ*m* matching approach. Specifically, we aimed to test the hypothesis that all precursor ions are potentially linked to all product ions in chimeric MS^2^ spectra of ultracomplex DOM. Our analysis was congruent with previous observations, showing losses of common Δ*m*’s (Table S-6) while also revealing more detail (Figure S-5c, Table S-7). Details are given in the Supporting Information (Note S-4). We found expected trends in losses of CO_2_, CO, and CH_2_ in both samples (Figure 2a–c, g–i and Table S-13). The predicted heteroatom content (O, N, S) of assigned molecular formulas and a widened tolerance window were used for further analysis of the uncovered structural information. Random Δ*m* matching would be expected if the calculated Δ*m* values were affected by low resolution, low sensitivity, or artifacts such as reactions in the instrument (e.g., between the collision and Orbitrap cell^78^). Instead, we found that (1) precursor ions with low ion abundance matched to less Δ*m* features (Figure S-7), (2) nonfragmented precursor ions matched to less or no Δ*m*’s (Figure S-8), and (3) identity of Δ*m* matches agreed with molecular formula prediction (e.g., loss of S-containing Δ*m*’s from S-containing precursor ions, etc.; Figures S-9 and S-10). Our evaluation also shows that Δ*m* matching not only helps in recalibration^79^ but also serves to check formula annotation, as it revealed unresolved precursor ion compositions interfering especially with CHOS precursor ions (related to Cl, P, and F). This means that (1) these atoms should be included for better coverage of elemental composition (i.e., prioritization) in our specific sample context and that (2) higher resolution power may be required to resolve S-, Cl-, P-, or F-containing precursor ion compositions.^1^ In summary, Δ*m* matching revealed an inherently structured biogeochemical signal of precursor ions that seem to fragment individually and was highly sensitive in detecting precursor-product ion pairs. This suggests that chimeric DOM data can be deconvoluted to reveal differences in molecular composition not visible from MS^1^ inspection.^23,80^ It should be stressed that these results will need further evaluation due to the small number of DOM precursor ions, *m*/*z* values, and samples analyzed here (159 in soil DOM, 221 in SRNOM), and that deconvolution should be further tested with better-characterized mixtures, including, e.g., structural analogues, artificial mixtures, or standard additions (spiking).^14,19,27,42,81^

### 3.4 Clustering with Reference Compound Δ*m*’s Reflects Structural Trends

DOM precursor ions from both samples were compared based on Δ*m* matching (Table S-7, see Section 3.1). We grouped DOM precursor ions, reference compounds, and Δ*m* features (list b) by two-way hierarchical clustering (Table S-14), i.e., matching of precursor ions across Δ*m* features and vice versa. In the following, precursor ion clusters will be referred to by letters (A–H) and Δ*m* clusters by numbers (1–7; Table 1). Based on the specificity of SIRIUS Δ*m* features (Table S-14), we defined five Δ*m* clusters found herein as structure-specific (Table 1, Figure 2d,e,j,k; and Table S-13).

**Table 1:**
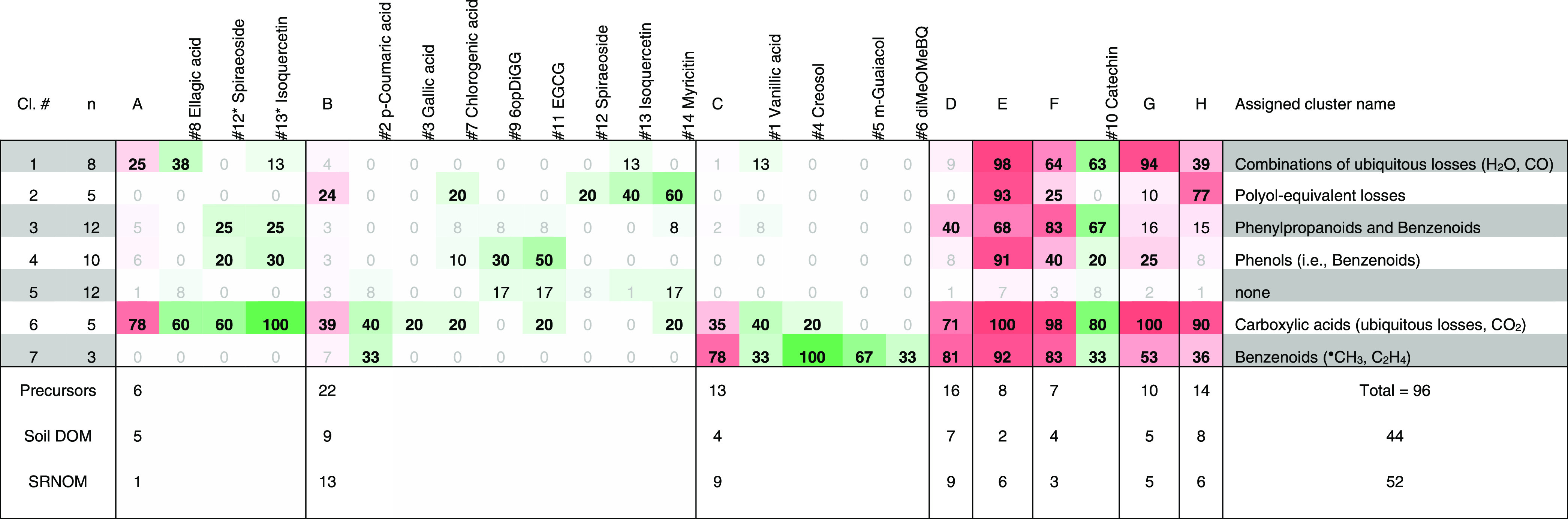
Summary of Two-Way Clustering of DOM Precursors (Highlighted in Red) and 14 Reference Compounds (Highlighted in Green)

* Numbers refer to Figure S1; #12* and #13* refer to MS3 spectra of flavonoid aglycons. Numbers are coverage in Δm matches compared to overall Δm’s per Δm cluster; values > 20% are highlighted in bold, values <10% are grayed out. Δm clusters are shown in rows (“Cl. #”, 1−7) and precursor clusters in columns (A–H, for details, see Table S14 and original clustering data in PANGAEA datasets). Additional columns show respective numbers of Δm matches (“n”) and assigned cluster name (compare Table S14). In the lower row, numbers of precursors per precursor cluster are given for both samples combined and individually. Few reference compounds clustered with precursor clusters D–H, which were dominated by DOM precursors with higher numbers of Δm matches. Compounds #7, #12, #13, and #14 contain polyol moieties; compounds #1, #4, #5, and #6 contain -methoxy and -methyl moieties (Figure S1)

Δ*m* features C_4_H_8_O_4_ (120.0423 Da, tetrose equivalent) and C_6_H_10_O_5_ (162.0528 Da, hexose equivalent), both members of cluster 2, were annotated to alcohols and polyols, carbohydrates, carbohydrate conjugates, and ether structures via SIRIUS (Table S-14). Reference compounds containing a polyol (quinic acid, #7) or a sugar (glucose, #12 and #13; mannose, #14) contributed Δ*m*’s to this cluster (Table 1).^51,52^ Cluster 2 Δ*m*’s matched to 18 soil DOM and 24 SRNOM precursor ions in the central van Krevelen plot despite the absence of “carbohydrate-like” precursor ions (lilac square, Figure 2d, j and o, q). The anticipated shift toward higher O/C and H/C ratios was nonetheless apparent (Figure 2e,f and k, l).

Cluster 3 and 4 Δ*m* features, partly specific to phenylpropanoid and benzenoid structures, were contributed by flavan-3-ols (#10, #11), flavon-3-ol aglycons (#12 and #13), and compounds containing cinnamic, coumaric, or gallic acid (#7, #9, #11).^28,33,52^ Precursor ions that matched to clusters 3 and 4 (soil DOM: *n* = 27 and *n* = 12; SRNOM: *n* = 29, *n* = 21) were found in the “lignin-like domain” (orange square in Figure 2e,k; orange circles in panels o, p, q). These C- or H-rich Δ*m*’s (e.g., C_8_H_10_O_2_ or C_7_H_4_O_4_) are likely no combinations of common O-rich losses (CO, H_2_O, or CO_2_) due to their low O/C and O/H ratios, but this requires further testing with model mixtures. Aliphatic chains could prevail as O-poor substructures in substituted cyclic core structures.^82,83^ Similar to the detection of polyol-equivalent Δ*m* matches outside the expected “carbohydrate domain”, gallate-equivalent losses were not matched to precursor ions in the anticipated “tannic domain” but to precursor ions outside of that box (red diamonds, turquoise square, Figure 2e, k; turquoise circle, panel p).

Among the most prominent features was the methyl radical loss,^35,49,50^ which matched to oxygen-poor DOM precursor ions and was one of three cluster 7 Δ*m* features (soil DOM: *n* = 18, average O/C = 0.33, SRNOM: *n* = 25, average O/C = 0.32, Figure 2f, l). The distribution of CH_3_^•^-yielding precursor ions was paralleled by CH_2_ (soil DOM: r = 0.60, *R*^2^ = 0.35, *n* = 127, *p* < 0.001; SRNOM: *r* = 0.63, *R*^2^ = 0.39, *n* = 144, *p* < 0.001) and CO losses (*r* = 0.55, *R*^2^ = 0.30, *n* = 127, *p* < 0.001; *r* = 0.58, *R*^2^ = 0.34, *n* = 144, *p* < 0.001), implying structural similarities (Figure 2f,l), e.g., condensed structures with aliphatic, lactone, or quinone moieties.^34^ CO and CH_3_^•^ were also annotated to benzenoid structures via SIRIUS (Table S-14). The methyl radical loss is an expected diagnostic Δ*m* of methoxylated aromatic rings as in lignin (orange square in Figure 2f, l; orange circles in panels m, n; see Note S-5), but was also matched to DOM precursor ions not classified as “lignin-like”.^18,31,35,49^ The Δ*m* features CH_3_^•^, CO and C_2_H_4_ were also linked to CH_4_ vs O series which describe regular 0.0364 Da increments in DOM that are formally annotated by an exchange of CH_4_ by O (Figure S-11).^37,38^ Concurrent losses of CO and C_2_H_4_ explained the presence of these increments on the product ion level and were paralleled by losses of CH_3_^•^. This finding could explain the ubiquitous presence of CH_4_ vs O series in nonfragmented DOM; for example, concurrent β-oxidation and de-carbonylation could be enzymatic MS^1^ analogues of the patterns seen in MS^2^.^26^ Alternatively, both methoxy-phenols (#4, #5) indicated an insertion of O for CH_4_ upon fragmentation (Note S-2, first section). Both observations may be relevant for the explanation of a key DOM property and require further exploration.

Taken together, matching to Δ*m* features derived from a small set of reference compounds revealed emerging clusters of precursor ion and Δ*m* feature families that may prove more indicative if constrained with further DOM and reference compound data.^14^ Anticipated structural domains were apparent but indicated clear overlap, which means that the same precursor ion was part of more than one Δ*m*-predicted structural class. For example, 27 classic lignin-like precursor ions were part of seven precursor ion clusters (B–H; Table S-15) with clear differences in potential structural composition. An extended analysis using >700 compound class-associated SIRIUS Δ*m* features (list c) substantiated these findings (Figures 3, S-12 and Note S-1).

CHNO and CHOS precursor ions matched with many of the S- and N-containing SIRIUS Δ*m* features (list c, spanning 3–78 S- and 4–251 N-containing Δ*m*’s in soil DOM and 0-154/0-350 in SRNOM; Tables S-16, S-17, S-18, and S-19). These represented on average 79 ± 19% (63 ± 31% in SRNOM) of all Δ*m* matches per CHOS precursor ion or 91 ± 7% (79 ± 28%) of all CHNO precursor ion matches (Note S-6). CHNO precursor ions were annotated with reduced forms of N (including aralkylamines, amino acids, carboximidamides, and dicarboximides/urea-containing compounds, Table S-20) but not to nitrate esters.^34,84,85^ S-containing Δ*m* matches indicated the potential presence of sulfonic, thiol, thioether, or aromatic CHOS compounds.^86^ These results show a wide potential diversity of N and S compounds in DOM that differs from earlier reports of mainly aromatic N and sulfonic S.^34,87,88^ As most of these studies analyzed marine DOM, the detection of more diverse sets of CHOS and CHNO precursor ions could relate to the terrestrial, less degraded DOM analyzed here.^16,89−91^ Further tests with N- and S-containing reference compounds and DOM samples are warranted to reveal the hidden diversity of CHNO/CHOS compounds and confirm potential structures, e.g., by NMR.

All in all, our results show that it may be possible to refine molecular structure representations in van Krevelen plots by deconvoluted MS^2^ data and that complementary precursor ion information could be used to assess false or biased Δ*m*-based class assignments (e.g., elemental composition, DBE, ionization, fragmentation sensitivity, ion mobility, polarity index, etc.).^13,55,58^ Fluorescence or NMR spectroscopy could add valuable information if DOM would be fractionated before MS^2^ data acquisition, i.e., to assess indirect (statistical) links of MS^2^ features with complementary forms of structural insight.^21,92−94^ Our findings must however be taken with caution for four reasons:

(1) SIRIUS Δ*m* features (list c) were not obtained on the same instrument and thus may include features that, although correlated with certain compound classes, may not appear in DOM under the same instrumental settings.
(2) SIRIUS Δ*m* features may be biased toward certain classes of compounds (Figure S-3), as our set of 14 aromatic compounds. Here, we only considered negative ESI mode data which is commonly employed for DOM analysis. Adding positive ESI or other ionizations would extend the range of Δm features and structural classes covered and likely decrease bias.^14,16,23,86^ The same applies to other fragmentation techniques than CID.
(3) Product ion abundance was disregarded in our analysis, but could be used to weigh probabilities of potential precursor-product ion pairs in future, potentially in combination with fragmentation energy gradients (fragmentation trees),^95^ moving *m*/*z* isolation windows, or ion accumulation time variation.^96^
(4) Despite a seemingly improved separation of extreme classes (high H/C ratios in fatty acids, high O/C ratios in carbohydrates, etc.), potential overlap in structural class boundaries remained considerable (Figures 3 and S-12).

Data-dependent and data-independent acquisition (DDA, DIA) techniques could be used to cover the whole mass range of precursor ions in DOM mass spectra in future, and are widely employed in LC-MS of complex mixtures.^16,27,97,98^ For example, Ludwig et al. presented a DIA scheme (SWATH-MS) that employs one precursor ion scan and 32 isolation windows of 25 Da width, covering 800 Da within 3.3 s; similar schemes are likely transferable to acquire full mass range data of directly injected DOM.^99^ Kurek et al. recently presented such data (*m*/*z* 392–408),^16^ Leyva et al. discerned fragmentation pathways and structural families (mass range *m*/*z* 261–477).^14^ The latter approach could be extended to include the diversity of structure-associated Δ*m* features presented here. Together, this shows that practicable tandem MS acquisition strategies are in reach and will enable deeper analyses of Δ*m* features in DOM soon.

### 3.5 Drivers of Differences in Δ*m* Matching between Soil DOM and SRNOM

Although matching among the two samples was largely consistent, slight differences were apparent in van Krevelen distributions (list b: Figure 2, list c: Figures 3 and S-12). We therefore tested the separation of precursor ion clusters by ordination (principal component analysis, Figure 4) using list b. Precursor ion clusters were clearly separated on Principal Components 1 and 2 which together held about 47% of variation. Most considered precursor ions were shared among samples (64%, 38 out of 59), only a small number was sample-specific (SRNOM = 14, Soil DOM = 7). Sample-specific precursor ions were found in clusters A (linked to carboxylic acids), B (phenols, polyols) and C (benzenoids, Table 1), the remaining clusters D–H were dominated by the shared precursor ions. Out of the 38 shared precursor ions, 30 (79%) grouped in the same precursor ion cluster despite a general trend to higher numbers of matches in SRNOM, but eight grouped differently (bold precursor ions in Figure 4). These differences in matching could be related to different chemistries, i.e., different isomeric/isobaric composition.^84^ For example, the cluster “switch” in C_11_H_14_O_6_ was largely explained by higher ion abundance and Δ*m* matches in SRNOM, while in C_23_H_22_O_4_, the effect could be partly linked to higher fragmentation resistance in SRNOM (Table S-21). Unfortunately, we only have data on initial ion abundance and fragmentation sensitivity from the soil DOM isolate; other precursor ion properties, however, showed very similar trends in both samples (Table S-21).

Similar clustering and Δ*m*-predicted structural classes (Figure S-12) in shared precursor ions could indicate a conserved structural composition. Likewise, Kurek et al. observed high similarity in photoionized (APPI) and IMPRD-fragmented DOM samples but observed clear differences in CHOS fragmentation.^16^ High similarities between DOM samples would be in line with stoichiometric principles (i.e., due to a large share in precursor ions between DOM samples) and could suggest that DOM processing diversifies, but also “randomizes” the molecular composition of each precursor ion (“universal” signal).^31,100,101^ High congruence of fragmentation patterns (and thus, Δ*m* matching) among DOM precursor ions has also been interpreted as a sign of similarly substituted but slightly differing core structures.^35,37^ The clusters devised here were small due to the relatively small number of precursor ions and *m*/*z* values analyzed, and thus may not detect significant differences between samples yet. However, even with our small set of precursor ions, the clustering by Δ*m* matching showed conserved differences in fragmentation between precursor ion clusters, and in part, even the same precursor ion in different samples. The fact that this could relate to differences in ion abundance (and therefore, possibly also ionization efficiency) or fragmentation sensitivity is intriguing and should be investigated across a wider range of DOM chemotypes using improved classification approaches as applied here (see also Section 3.4).^14^ In line with this, potential compositional differences between DOM samples became more apparent when more Δ*m* features were used for the clustering (list c instead of list b; Figures 3 and S-12).

### 3.6 Ion Abundance Is Linked to Δ*m* Matching Frequency and Structural Diversity

Ion abundance was the most important driver for Δ*m* matching in both samples and highest in the van Krevelen plot “region” usually assigned to ubiquitous lignin structures or carboxyl-rich aliphatic molecules.^59,83^ This region also parallels with a maximum in potential underlying chemodiversity,^30,102^ which could explain why these signals are ubiquitously found and especially dominant in recycled DOM.^90,103^ Δ*m* matching showed potential to reveal this underlying chemodiversity effect and was therefore compared to numbers of structure suggestions and Δ*m*-predicted compound classes per precursor ion (Figure 5). Numbers of Δ*m* matches were significantly and positively related to the number of structure suggestions in absolute terms and for specific compound classes (Table S-21). The correlation between Δ*m*-predicted and suggested compound classes was surprisingly similar in both samples and significant for almost all benzenoid-type (benzopyrans, methoxybenzenes, anisoles, phenols, etc.) and most phenylpropanoid-type structures (flavonoids, linear 1,3-diarylpropanoids). Among the organic acids, only vinylogous acids stood out (i.e., containing carboxylic acid groups with insertions of C=C bond(s)). Significant correlations were also found for pyrans, acryloyl compounds, carbohydrates, aryl ketones, and alkyl aryl ethers (fatty acids and analogues only in SRNOM).

The positive link between ion abundance and numbers of Δ*m* matches on the one hand and predicted and suggested structures on the other indicates that ion abundance may be linked to the number of structural isomers and isobars per molecular formula in FTMS spectra of DOM and explains why Δ*m***-**defined structural classes showed strong overlap in this study. It also provides additional support to our assumption that all precursor ions potentially contribute to all product ions in DOM: The patterns revealed through Δ*m* matching were largely congruent with the independent estimate of structural composition by natural product databases. The fact that only some classes of compounds (mainly benzenoids and phenylpropanoids) showed significant correlations could point to bias toward plant natural products in the databases employed here; in turn, this means that the inclusion of other structure databases and the additional assignment of Δ*m*’s not only to their host structures but also to host organisms (e.g., in GNPS^65^) could reveal further clues about the potential sources of molecular formulas in DOM.

We propose that the number of Δ*m* matches could be interpreted as a novel, relatively easily accessible measure to account for a precursor ions’ underlying potential structural diversity. Such information could help to better understand the mechanisms of DOM formation and persistence in the environment. Our results encourage further studies on the Δ*m* matching behavior of synthetic mixtures of known structures and across DOM chemotypes, and the improved bioinformatic exploitation of chimeric (LC-) FTMS^n^ data of complex organic mixtures.^14,104−106^ We acknowledge that natural product and in silico databases are far from being complete, same as the database of annotated Δ*m* matches we used here, despite its large coverage of ∼18 000 unique structures and ∼11 500 Δ*m*’s (Figure S-3). For example, precursor ions with low mass defects showed exceptionally few structural hits, indicating bias in natural product databases (Figure S-13).^18^ These structures were easily fragmented and yielded few Δ*m* matches in our analysis. CHO precursor ions were double as likely to yield a suggestion than N- and S-containing precursor ions. These observations show that DOM contains unique molecular structures to be identified in future, potentially through the application of a wider range of ionization and fragmentation techniques that reduce structural bias.^14,16,23^

## 4 Implications

Tandem MS data of complex samples such as dissolved organic matter (DOM) is impeded by the co-fragmentation of precursor ions with similar nominal mass, and further complicated by the contribution of potential isomers and isobars. We employed an approach that analyzes the pairwise Δ*m*’s between all precursor and product ions (Δ*m* matrix). Using a very limited set of precursor ion features from two samples, we found potential signs of structural imprints related to e.g., benzenoids, phenylpropanoids, carbohydrates, sulfonic acids, thiols, thioethers, and amino acids. The successful matching of indicative Δ*m* features and precursor ion clustering suggests a recognizable source imprint of primary or recycled plant remains in DOM. Tests with more DOM samples and artificial/treated mixtures (e.g., spiked DOM, or enzyme-degraded DOM) are required to test the assumptions employed here and to improve classifications by Δ*m* clustering. Our first results indicate that FTMS^2^ data may be useful to differentiate molecular composition on the molecular formula level and that ion abundance and fragmentation sensitivity are two key variables that explain differences in MS^2^ data within and among samples. This is intriguing because a shared molecular formula could harbor a completely different set of structures, and larger sets of DOM data would improve the detection of these differences. Generally, our findings support the view that regions of the van Krevelen plot are associated with indicative Δ*m*’s that relate to stoichiometric differences between compound classes. The most abundant precursor ions however showed a mixed MS^2^ signal that caused boundary overlap of these “Δ*m*-defined regions” (Figures 3 and 5e, f). While this finding is in line with known patterns of structural diversity and partly explains the ubiquitous presence of abundant DOM signals, it introduces a new paradigm to the interpretation of DOM FTMS data by assigning unknown precursor ions to *multiple* structural categories instead of just one (Figure S-12). Further evaluation of both natural and spiked/treated complex mixtures, constantly growing MS databases, and comprehensive decomplexation methods (LC-MS, IMS) will together provide fundamental insights into the deconvolution of chimeric spectra from complex samples, and ultimately show the potential to unfold the hidden molecular diversity and identity of DOM.

## Supporting Information Available

Table S-1: Information on reference compounds and solutions used in this study; Table S-2: Instrument settings for fragmentation experiments; Table S-3: Recalibration peaks used for reference compound in FTMS measurements; Table S-4: Precursor and major product ions of the 14 reference compounds; Table S-5: Results of reference compound’s tandem MS data analysis with CSI:FingerID; Table S-6: List of reported DOM Δ*m* features from MS1 and MS2 studies; Table S-7: List of all 50 + 5 Δ*m* features extracted from the reference compound dataset; Table S-8: Properties of four isolated nominal masses (IPIMs) at different NCE levels; Table S-9: Overview of correlations between key properties of the IPIM 241; Table S-10: Overview of correlations between key properties of the IPIM 301; Table S-11: Overview of correlations between key properties of the IPIM 361; Table S-12: Overview of correlations between key properties of the IPIM 417; Table S-13: Lists of Δ*m* values used for analyzing matching patterns in van Krevelen space; Table S-14: Matching behavior of precursor ion clusters against Δ*m* features (Table S-7); Table S-15: Lignin-like precursor ion formulas and their molecular properties and clustering; Table S-16: S-containing precursor ion formulas in soil porewater DOM; Table S-17: N-containing precursor ion formulas in soil porewater DOM; Table S-18: S-containing precursor ion formulas in SRNOM; Table S-19: N-containing precursor ion formulas in SRNOM; Table S-20: Structural class-correlated Δ*m* features matched to CHOS or CHNO precursor ions; Table S-21: Correlations of selected precursor ion properties with PC scores (Figure 4); Table S-22: Correlations between structure hits and specific Δ*m* features in CHO precursor ions; Figure S-1: Overview of reference compounds used in the study; Figure S-2: Mass accuracy assessment based on reference compound Δ*m*’s; Figure S-3: Distribution of compound classes in SIRIUS-annotated molecular structures; Figure S-4: Distribution of exemplary known structures in chemical space; Figure S-5: Orbitrap tandem MS of soil porewater DOM; Figure S-6: Comparison of matches to the two short Δ*m* lists in relation to *m*/*z* and NCE; Figure S-7: Δ*m* matches in relation to precursor ion abundance in soil DOM; Figure S-8: Δ*m* matches in relation to precursor ion fragmentation sensitivity in soil DOM; Figure S-9: Matching assessment with SIRIUS Δ*m*’s (molecular formula check); Figure S-10: Changes in Δ*m* matching frequency upon widening of tolerance window; Figure S-11: Link between matches to CH_3_^•^, CO and C_2_H_4_ and CH_4_ vs O exchange series; Figure S-12: Compositional variation of individual precursor ions in van Krevelen space; Figure S-13: Effect of mass defect on the number of structure suggestions; Note S-1: Supporting experimental details; Note S-2: Detailed description of reference compound fragmentation behavior; Note S-3: Behavior of nonresponsive DOM precursor ions; Note S-4: Δ*m* matching: Proof-of-concept data and key findings; Note S-5: Potential esterification of DOM by methanol during SPE and storage; and Note S-6: Structural insight into N- and S-containing DOM precursor ions (PDF)

## Notes

All tandem MS data can be found on the Mass Spectrometry Interactive Virtual Environment (MassIVE) under the following links: ftp://massive.ucsd.edu/MSV000087117/(soil DOM data), ftp://massive.ucsd.edu/MSV000088869/(SRNOM data), and ftp://massive.ucsd.edu/MSV000087133/(reference compound data) (Data Set S-1, raw peak data, *.mzML files). All other data associated with this manuscript (extensive tables, Δ*m* feature lists, Δ*m* specificity, two-way clustering table, processed data used to create figures, etc.) are available online free of charge from the PANGAEA Data Publisher under the following citation: Simon, Carsten; Dührkop, Kai; Petras, Daniel; Roth, Vanessa-Nina; Böcker, Sebastian; Dorrestein, Pieter C; Gleixner, Gerd (2022): Structural insight into conifer forest topsoil and blackwater dissolved organic matter by Orbitrap tandem MS mass difference matching. PANGAEA, https://doi.pangaea.de/10.1594/PANGAEA.944673 (Data Set S-2, processed data as *.xlsx files).
