# Supplementary material for: Mass Difference Matching Unfolds Hidden Molecular Structures of Dissolved Organic Matter
Source: Environ Sci Technol. 2022 Jul 14;56(15):11027–40. doi: 10.1021/acs.est.2c01332 (PMC9352317; doi:10.1021/acs.est.2c01332)
Supplement: Supplementary file 1 — es2c01332_si_001.pdf [file es2c01332_si_001.pdf]

Supporting Information

Mass difference matching unfolds hidden molecular structures of dissolved organic matter

Carsten Simon[1,†,#,*], Kai Dührkop[2], Daniel Petras[3,4], Vanessa-Nina Roth[1,§], Sebastian Böcker[2], Pieter C. Dorrestein[3], and Gerd Gleixner[1,*]

[1] Molecular Biogeochemistry, Department of Biogeochemical Processes, Max Planck Institute for Biogeochemistry, Hans-Knöll-Straße 10, 07745 Jena, Germany

[2] Chair for Bioinformatics, Faculty of Mathematics and Computer Science, Friedrich Schiller University Jena, Ernst-Abbe-Platz 2, 07743 Jena, Germany

[3] Collaborative Mass Spectrometry Innovation Center, Skaggs School of Pharmacy and Pharmaceutical Sciences, University of California San Diego, 9500 Gilman Drive, MC 0657, La Jolla, CA 92093-0657, United States of America

[4] CMFI Cluster of Excellence, Interfaculty Institute of Microbiology and Medicine, University of Tübingen, Auf der Morgenstelle 24, 72076 Tübingen, Germany

Present Addresses

† C.S.: Institute for Biogeochemistry and Pollutant Dynamics, ETH Zürich, Universitätsstrasse 16, 8092 Zürich, Switzerland
# C.S.: Swiss Federal Institute of Aquatic Science and Technology (Eawag), Department Water Resources and Drinking Water, Überlandstrasse 133, 8600 Dübendorf, Switzerland
§ V.-N. R.: Thüringer Landesamt für Umwelt, Bergbau und Naturschutz (TLUBN), Göschwitzer Straße 41, 07745 Jena, Germany

*Correspondence: carsten.simon@usys.ethz.ch (Carsten Simon), gerd.gleixner@bgc-jena.mpg.de (Gerd Gleixner)

Contents

Introduction……………………………………………………………………………………S4
Table S-1. Information on reference compounds and solutions used in this study……………...S5
Table S-2. Instrument settings for fragmentation experiments………………………………… S6
Table S-3. Recalibration peaks used for reference compound FTMS measurements…………...S6
Table S-4. Precursor and major product ions of the 14 reference compounds…………………..S7
Table S-5. Results of reference compound's tandem MS data analysis with CSI:FingerID……S8
Table S-6. List of reported DOM Δm features from MS^1 studies and MS^2 studies……………..S9
Table S-7. List of all 50+5 Δm features extracted from the reference compound dataset……. S11
Table S-8. Properties of isolated nominal masses (IPIMs) at four different NCE levels...........S13
Table S-9. Overview of correlations between key properties of the IPIM at m/z 241………… S14
Table S-10. Overview of correlations between key properties of the IPIM at *m/z* 301……….. S15
Table S-11. Overview of correlations between key properties of the IPIM at *m/z* 361……….. S16
Table S-12. Overview of correlations between key properties of the IPIM at *m/z* 417……….. S17
Table S-13. Lists of Δm values used for analysing matching patterns in Van Krevelen space.. S18
Table S-14. Matching behavior of precursor ion clusters against Δm features (Table S-7)……S19
Table S-15. Lignin-like precursor ion formulas and their molecular properties and clustering..S21
Table S-16. S-containing precursor ion formulas in soil porewater DOM…………………… S23
Table S-17. N-containing precursor ion formulas in soil porewater DOM……………………S24
Table S-18. S-containing precursor ion formulas in SRNOM………………………………… S25
Table S-19. N-containing precursor ion formulas in SRNOM…………………………………S27
Table S-20. Structural class-correlated Δm features matched to CHOS or CHNO precursors...S28
Table S-21. Correlations of selected precursor ion properties with PC scores (Figure 4)…….. S29
Table S-22. Correlations between structure hits and specific Δm features in CHO precursors..S30
Figure S-1. Overview of reference compounds used in the study……………………………...S32
Figure S-2. Error assessment of reference compound Δm's…………………………………... S33
Figure S-3. Distribution of compound classes in SIRIUS-annotated molecular structures…… S34
Figure S-4. Distribution of exemplary known structures in chemical space………………….. S34
Figure S-5. Orbitrap tandem MS of soil porewater DOM……………………………………...S35
Figure S-6. Comparison of matches to the two short Δm lists in relation to m/z and NCE……S36
Figure S-7. The number of Δm matches in relation to precursor ion abundance………………S37
Figure S-8. The number of matches in relation to precursor ion fragmentation sensitivity……S38

Figure S-9. Matching assessment with SIRIUS Δm's (Molecular formula check)…………….S39
Figure S-10. Changes in Δm matching frequency upon widening of tolerance window……… S41
Figure S-11. Link between matches to $CH_3^{\bullet}$, CO and C_2H_4 and CH_4 vs. O exchange series….S42
Figure S-12. Compositional variation of individual precursor ions in Van Krevelen space…...S43
Figure S-13. Effect of mass defect on the number of structure suggestions…………………... S44
Note S-1. Supplementary experimental details……………………………………………... S45
Note S-2. Detailed description of reference compound fragmentation behavior……………… S49
Note S-3. Properties of selected IPIMs and behavior of non-responsive DOM precursor ions..S51
Note S-4. Δm matching: Proof-of-concept data and key findings…………………………….. S51
Note S-5. Potential esterification of DOM by methanol during SPE and storage…………….. S51
Note S-6. Structural insight into N- and S-containing DOM precursor ions………………….. S52
Supplementary Material References………………………………………………………….S53

Introduction

This Supporting Information file contains 22 supporting tables (**Tables S-1** to **S-22**), twelve supporting figures (**Figures S-1** to **S-12**), six supporting text resources (**Notes S-1** to **S-6**), and two supporting data sets (Data Sets S-1 to S-2). This file contains 69 references.

Supporting data to reproduce our findings can be found online, free of charge.

Data Set S-1. Tandem MS raw data can be found on the Mass Spectrometry Interactive Virtual Environment (MassIVE) under the following links as *.mzML files:

- ftp://massive.ucsd.edu/MSV000087117/ (soil DOM data)
- ftp://massive.ucsd.edu/MSV000088869/ (SRNOM data)
- ftp://massive.ucsd.edu/MSV000087133/ (reference compound data)

Data Set S-2. Six Supporting Information *.xlsx files are available via PANGAEA Data Publisher under the following citation[1]:

Simon, Carsten; Dührkop, Kai; Petras, Daniel; Roth, Vanessa-Nina; Böcker, Sebastian; Dorrestein, Pieter C; Gleixner, Gerd (2022): Structural insight into conifer forest topsoil and blackwater dissolved organic matter by Orbitrap tandem MS mass difference matching. PANGAEA, https://doi.pangaea.de/10.1594/PANGAEA.944673

- "ds01", contains the processed reference compound data and fragmentation sensitivities of 14 phenolic reference compounds, and general information on the analyzed parts of the DOM mass spectrum (molecular indices, number of precursor ions, number of product ions). Contains four data sheets.
- "ds02" – "ds05" each contain the aligned DOM molecular composition data obtained at different collision energies for four mass windows ("ds02", m/z 241; "ds03", m/z 301; "ds04", m/z 361; "ds05", m/z 417) and include mass difference matching results (non-indicative Δm features, reference compound (14 phenolics) Δm features, and SIRIUS library spectra Δm features) for both DOM samples (SRNOM, only NCE25). Each file contains five data sheets.
- "ds06" contains the full Δm feature lists (including the full SIRIUS-annotated list for negative ESI mode and a TOP1000 Δm feature list for positive ESI mode) and all data tables to reproduce analyses and figures from the manuscript (e.g., aggregated matching results Ddd
- "ds07" contains the two-way clustering result of 149 CHO precursors with 725 most common (matched >=10 times across all precursor ions) SIRIUS-annotated Δm features from list c (see main text, experimental section) and the derived statistics on structural composition.

Table S-1. Information on reference compounds and solutions used in this study (structural formulas, see **Fig. S-1**).

ID	Reference compound	MW [Da]	Formula	Supplier	Weighed portion [mg]	Final concentration [ppm]
1	Vanillic acid	168.14	$C_8H_8O_4$	Sigma-Aldrich	1.98	200
2	4-Hydroxycinammic acid	164.04	$C_9H_8O_3$	Sigma-Aldrich	3.91	200
3	Gallic acid	170.12	$C_7H_6O_5$	Sigma-Aldrich	3.89	200
4	2-Methoxy-4-methylphenol	138.16	$C_8H_{10}O_2$	Sigma-Aldrich	10.9	200
5	3-Methoxyphenol	124.14	$C_7H_8O_2$	Sigma-Aldrich	13.1	200
64	2,3-Dimethoxy-5-methyl-1,4-benzoquinone	182.18	$C_9H_{10}O_4$	Alfa Aesar	2.5	200
7	Chlorogenic acid	354.31	$C_{16}H_{18}O_9$	Sigma-Aldrich	3.57	200
8	Ellagic acid	302.19	$C_{14}H_6O_8$	Sigma-Aldrich	0.99	< 124
9	6-o,p-Coumaryl-1,2-digalloylglucose	630.51	$C_{29}H_{26}O_{16}$	Sigma-Aldrich	0.35	39
10	Catechin	290.27	$C_{15}H_{14}O_6$	Sigma-Aldrich	1.35	100
11	Epigallocatechin gallate	458.37	$C_{22}H_{18}O_{11}$	Santa Cruz	0.98	100
12	Spiraeoside	464.38	$C_{21}H_{20}O_{12}$	Carl Roth	0.85	100
13	Isoquercetin	464.38	$C_{21}H_{20}O_{12}$	Santa Cruz	0.49	55
14	Myricitrin	464.38	$C_{21}H_{20}O_{12}$	Sigma-Aldrich	0.31	33

Table S-2. Instrument settings for fragmentation experiments. Settings were optimized for reference compound detection to obtain high-quality Δm data for known structures in order to allow search for structural analogs in DOM.

Method stage	Factor	Reference compounds	DOM samples
Sample	DOC [ppm]<br>Solvent<br>Flow [µl*min^{-1}]	Max. 200, see Table S-1<br>50/50 MeOH/ H_2O<br>10	100<br>50/50 MeOH/ H_2O<br>7
Electrospray ionization	Ionization mode<br>Source fragmentation [eV]<br>Needle position<br>Sheath gas [a.u.]<br>Aux gas [a.u.]<br>Sweep gas [a.u.]<br>Spray voltage [kV]<br>Capillary Temp. [°C]	Negative<br>0<br>Variable<br>Variable<br>Variable<br>0<br>Variable<br>275	Negative<br>40<br>D<br>25<br>0<br>0<br>2.65<br>275
Ion optics	S-Lens RF level [%]<br>Multipole 00 offset [V]<br>Lens 0 [V]<br>Multipole 0 offset [V]<br>Lens 1 [V]<br>Multipole 1 offset [V]<br>Multipole RF Amplitude [Vp-p]<br>Front Lens [V]	Variable<br>1.1<br>3.2<br>9.4<br>17.3<br>13.8<br>800<br>10.3	70<br>1.0<br>3.2<br>9.4<br>17.2<br>13.2<br>792<br>10.0
Tandem MS	Act Q<br>Act time [ms]<br>Isolation window [amu]<br>Normalized collision energies	0.25<br>0.1<br>1<br>NCE: 15, 20, 25	0.25<br>0.1<br>1<br>NCE: 15, 20, 25
MS Detection	Max. Inject time [ms]<br>Automatic Gain Control™<br>Scans per MS^2 experiment [n]<br>Resolution<br>Transient length [s]<br>Profile mode<br>Scan range [m/z]	5<br>5E4<br>50<br>240.000<br>0.8<br>Reduced<br>Variable	2<br>5E4<br>150<br>240.000<br>0.8<br>Reduced<br>Variable

Table S-3. Recalibration peaks used for reference compound Orbitrap tandem MS measurements. Compound **#1** – **#6** were only recalibrated by precursor ion exact *m/z*. References: [2]Ncube et al., 2014; [3]Mullen et al., 2003; [4]Fischer et al., 2011; [5]Engström et al., 2015; [6]Wyrepkowski et al., 2014; [7]Rockenbach et al., 2012; [8]Gu et al., 2003; [9]Miketova et al., 2000; [10]Yuzuak et al. 2018; [11]Fabre et al., 2001; [12]Saldanha et al., 2013.

ID	Reference compound	Precursor ion exact *m/z*	Product ions used as recal peaks, exact m/z (Formula)	Reference
7	Chlorogenic acid	353.088	191.0561 (C7H11O6), 179.035 (C9H7O4), 109.0295 (C6H5O2)	[1]
8	Ellagic acid	300.999	229.0143 (C12H5O5), 185.0244 (C11H5O3), 145.0296 (C9H5O2)	[2, 3, 4, 5]
9	6-o,p-Coumaryl-1,2-digalloylglucose	629.115	459.0933 (C22H19O11), 465.0675 (C20H17O13), 169.0142 (C7H5O5), 163.0401 (C9H7O3)	[6, 7]
10	Catechin	289.072	109.0295 (C6H5O2)	[6, 7, 8, 9]
11	Epigallocatechin gallate	457.078	169.0142 (C7H5O5)	[7, 8]
12	Spiraeoside	463.088	301.0354 (C15H9O7), 178.9986 (C8H3O5), 107.0139 (C6H3O2)	[10]
13	Isoquercetin	463.088	301.0354 (C15H9O7), 178.9986 (C8H3O5), 151.0037 (C7H3O4), 107.0139 (C6H3O2)	[4, 10]
14	Myricitrin	463.088	316.0225 (C15H8O8), 317.0303 (C15H9O8), 178.9986 (C8H3O5), 151.0037 (C7H3O4)	[10, 11]

Table S-4. Precursor and major product ions of the 14 reference compounds. The deprotonated precursor ion form was always dominant, except for compound **#6,** where the radical anion form dominated. Numbers in brackets indicate %-ion abundance relative to base peak (=100%) and the respective normalized collision energy (NCE) at which mass spectra were acquired. In some cases, further MS^3 experiments (note asterisk at ID) were conducted at NCE 20 (#6*) or NCE 20 and 25 (#12*, #13*).

**ID**	**Reference compound**	**Formula**	**Precursor ion *(m/z)***	**Product ions (*m/z*)**
**1**	Vanillic acid	C8H8O4	167.0350 (35; at NCE 25)	**152.0115** (92); **123.0452** (100); 109.0925 (<1); **108.0217** (18); 95.0503 (<1)
**2**	4-Hydroxycinammic ac.	C9H8O3	163.0401 (25; at NCE 25)	145.0296 (<1); 121.0296 (<1); **119.0295** (100); 93.0346 (<1)
**3**	Gallic acid	C7H6O5	169.0142 (16; at NCE 25)	**125.0244** (100)
**4**	Creosol	C8H10O2	137.0608 (8; at NCE 25)	**122.0374** (100); **109.0295** (2); 95.0503 (<1); 95.0139 (<1); 93.0346 (<1)
**5**	m-Guaiacol	C7H8O2	123.0452 (8; at NCE 25)	**108.0217** (100); **95.0139** (1)
**6**	2,3-Dimethoxy-5-methyl-1,4-benzoquinone	C9H10O4	182.0585 (6; at NCE 20)	**167.0350** (100); 152.0115 (<1)
**6***	MS^3 of #6 (Methyl loss) isolated at NCE 25	C8H8O4	167.03498 (1; at NCE 20)	**152.0115** (100); **139.0401** (3); **125.0245** (1); 121.0296 (<1)
**7**	Chlorogenic acid	C16H18O9	353.0878 (7; at NCE 20)	**191.0561** (100); **179.0350** (4); 161.0245 (<1); 109.0295 (<1); 99.0451 (<1)
**8**	Ellagic acid	C14H6O8	300.9990 (100; at NCE 25)	**257.0092** (5); **229.0143** (5); **201.0193** (1); **185.0244** (3); 163.0401 (<1); 161.0245 (<1); 145.0296 (<1)
**9**	6-o,p-Coumaryl-digalloyl-Glucose	C29H26O16	629.1148 (2; at NCE 25)	**477.1039** (8); **465.0675** (100); **459.0933** (48); **313.0565** (3); **271.0459** (5); 193.0142 (<1); 187.0401 (<1)
**10**	Catechin	C15H14O6	289.0718 (22; at NCE 25)	**271.0612** (3); **247.0612** (5); **245.0820** (100); **231.0299** (6); **227.0714** (2); **205.0506** (35); **203.0714** (8); 188.0479 (1); 187.0401 (1); **179.035** (15); **167.035** (2); **165.0194** (4); 163.0401 (<1); 162.0323 (<1); **161.0609** (2); 161.0245 (<1); **151.0401** (2); **125.0244** (5); 123.0452 (<1); 121.0296 (<1); **109.0295** (2); 99.0451 (<1); 93.0346 (<1)
**11**	Epigallocatechin Gallate	C22H18O11	457.0776 (8; at NCE 20)	**413.0879** (2); **331.0459** (95); **319.0458** (5); **305.0666** (33); **287.0561** (10); **275.0561** (3); **269.0455** (5); **193.0142** (12); **169.0142** (100)
**12**	Spiraeoside	C21H20O12	463.0882 (3; at NCE 20)	**301.0354** (100)
**12***	MS^3 of #12 (Aglycone) isolated at NCE 20	C15H10O7	301.03537 (35; at NCE 25)	300.0275 (<1); **273.0405** (10); **257.0455** (9); **229.0506** (2); **193.0142** (4); **178.9986** (100); **151.0037** (82); **121.0296** (1); **107.0138** (3)
**13**	Isoquercetin	C21H20O12	463.0882 (1; at NCE 25)	**343.0459** (2); **301.0354** (100); **300.0275** (22)
**13***	MS^3 of #13 (Aglycone) isolated at NCE 25	C15H10O7	301.03537 (32; at NCE 25)	300.0275 (<1); **283.0248** (3); **273.0405** (11); **257.0455** (9); **255.0299** (1); **239.0350** (2); **229.0506** (3); **211.0401** (1); **193.0142** (4); **178.9986** (100); **151.0037** (88); **121.0296** (2); **107.0138** (4)
**14**	Myricitrin	C21H20O12	463.0882 (2; at NCE 25)	**359.0408** (2); **337.0564** (1); **317.0303** (50); **316.0225** (100); **178.9986** (3)

Table S-5. Results of reference compound's tandem MS data analysis with SIRIUS[13] (for product ion annotation and fragmentation tree generation) and CSI:FingerID[14] (for structure prediction by comparison of fragmentation trees).

ID	Reference compound/ neutral molecular formula	NCE Levels	Precursor ion	SIRIUS: Peaks and assigned formulas	SIRIUS: Fragmentation tree	CSI:FingerID result
**1**	Vanillic acid ($C_8H_8O_4$)	10,15,20,25	[M-H]- 167.03498	6 peaks, 83% peaks with assigned formula, 99.87 total explained intensity, -0.01 ppm absolute error (Median)	Correct formula = tree#1, Tree score 11.97 (100%), correct tree has lowest ppm error	Score 86.31%, 1st hit
**2**	4-Hydroxy-cinnamic acid ($C_9H_8O_3$)	10,20,25	[M-H]- 163.04007	2 peaks, 100% peaks with assigned formula, 100 total explained intensity, 0 ppm absolute error (Median)	Correct formula = tree#1, Tree score 12.71 (99.94%), correct tree has lowest ppm error	no prediction possible
**3**	Gallic acid ($C_7H_6O_5$)	10,15,20,25	[M-H]- 169.01425	2 peaks, 100% peaks with assigned formula, 100 total explained intensity, -0.01 ppm absolute error (Median)	Correct formula = tree#1, Tree score 2.19 (98.63%), correct tree has lowest ppm error	no prediction possible
**4**	Creosol ($C_8H_{10}O_2$)	10,20,25	[M-H]- 137.06080	4 peaks, 100% peaks with assigned formula, 100 total explained intensity, 0.16 ppm absolute error (Median)	Correct formula = tree#1, Tree score 10.95 (99.95%), correct tree has lowest ppm error	Score 64.79%, 1st hit
**5**	m-Guaiacol ($C_7H_8O_2$)	10,20,25	[M-H]- 123.04515	3 peaks, 100% peaks with assigned formula, 100 total explained intensity, 0.23 ppm absolute error (Median)	Correct formula = tree#1, Tree score 7.4 (99.91%), correct tree has lowest ppm error	Score 58.04%, 2nd hit
**6**	2,3-Dimethoxy-5-methyl-1,4-benzoquinone ($C_9H_{10}O_4$)	10,15,20	[M]- 182.05846	3 peaks, 100% peaks with assigned formula, 100 total explained intensity, -0.01 ppm absolute error (Median)	Correct formula = tree#2, Tree score 5.95 (41.87%), correct tree has lowest ppm error	Score 57.32% (wrong isomer)
**7**	Chlorogenic acid ($C_{16}H_{18}O_9$)	10,15,20	[M-H]- 353.08781	6 peaks, 100% peaks with assigned formula, 100 total explained intensity, -0.34 ppm absolute error (Median)	Correct formula = tree#1, Tree score 7.15 (99.28%), correct tree has lowest ppm error	Score 89.60%, 1st hit
**8**	Ellagic acid ($C_{14}H_6O_8$)	10,20,25,30,35,40	[M-H]- 300.99899	55 peaks, 85% peaks with assigned formula, 99.25 total explained intensity, -0.1 ppm absolute error (Median)	Correct formula = tree#2, Tree score 54.17 (7.71%), correct tree has lowest ppm error	Score 80.83%, 1st hit
**9**	6-op-Coumaryl-digalloyl-Glucose ($C_{29}H_{26}O_{16}$)	10,15,20,25	[M-H]- 629.11481	15 peaks, 87% peaks with assigned formula, 99.6 total explained intensity, -0.19 ppm absolute error (Median)	Correct formula = tree#1, Tree score 19.53 (26.29%), correct tree has lowest ppm error	Score 73.33 %, 1st hit
**10**	Catechin ($C_{15}H_{14}O_6$)	10,15,20,25, 30	[M-H]- 289.07176	41 peaks, 98% peaks with assigned formula, 99.94 total explained intensity, -0.03 ppm absolute error (Median)	Correct formula = tree#1, Tree score 59.49 (100%), correct tree has lowest ppm error	Score 82.12% (wrong isomer)
**11**	Epigallocatechin Gallate ($C_{22}H_{18}O_{11}$)	10,15,20	[M-H]- 457.07764	18 peaks, 67% peaks with assigned formula, 98.34 total explained intensity, 0.25 ppm absolute error (Median)	Correct formula = tree#1, Tree score 27.55 (68.78%), correct tree close to lowest ppm error	Score 84.36 %, 1st hit
**12**	Spiraeoside ($C_{21}H_{20}O_{12}$)	10,15,20	[M-H]- 463.08820	5 peaks, 40% peaks with assigned formula, 98.86 total explained intensity, -0.01 ppm absolute error (Median)	Correct formula = tree#1, Tree score 4.87 (35.26%), correct tree has lowest ppm error	no prediction possible
**13**	Isoquercetin ($C_{21}H_{20}O_{12}$)	10,15,20,25	[M-H]- 463.08820	9 peaks, 78% peaks with assigned formula, 99.61 total explained intensity, 0.24 ppm absolute error (Median)	Correct formula = tree#1, Tree score 4.65 (56.76%), correct tree has lowest ppm error	Score 92.25 %, 1st hit
**14**	Myricitrin ($C_{21}H_{20}O_{12}$)	10,15,20,25	[M-H]- 463.08820	12 peaks, 83% peaks with assigned formula, 99.5 total explained intensity, 0.26 ppm absolute error (Median)	Correct formula = tree#1, Tree score 12.79 (95.61%), correct tree has lowest ppm error	Score 86.90 %, 1st hit

Table S-6. List of reported DOM Δm features from MS^1 studies (within-spectrum Δm's or "mass spacings", as in refs [14], [15], [17] and [18]) and MS^2 studies (tandem MS Δm's, as presented in refs [19]–[22]). Occurrence refers to matches across 159 precursor ions investigated. References: [15]Zhang et al. 2014; [16]Longnecker & Kujawinski 2016; [17]Cortés-Francisco & Caixach 2015; [18]Kunenkov et al. 2009; [19]Kujawinski & Behn 2006; [20]Witt et al. 2009; [21]Osterholz et al. 2015; [22]Hawkes et al. 2018; [23]Pohlabeln & Dittmar 2015.

Formula	Exact mass difference	Reference(s)	Explanation
$C_{-1}H_{-2}O$	1.979265	[14, 15]	Acetic acid/ -H_2O and -CO_2
H_2	2.01565	[14 - 18]	(De-)hydrogenation
C	12	[14 - 16]	Glyoxylic acid/ -H_2O and -CO_2
OH_{-2}	13.979265	[15]	O/H_2 exchange
CH_2	14.01565	[14 - 18]	(De-)methylation
O	15.994915	[14 - 18]	(De-)hydroxylation/ Oxygen
CH_4	16.0313	[19]	Methane
H_2O	18.010565	[16, 19 - 21, a.o.]	Water
$CH_{-2}O$	25.979265	[14]	C=O insertion
CHN	27.010899	[14]	Formimino transfer
CO	27.994915	[14 - 17]	Formyl transfer/ Carbon Monooxide
C_2H_4	28.031300	[14 - 16]	β-oxidation/ fatty acid synthesis
$H_{-1}NO$	28.990164	[14]	Nitrosylation
CHO	29.00274	[16]	Formyl-group related
CH_2O	30.010565	[14, 16, 17]	Hydroxymethyl transfer
S	31.972072	[22]	Sulfur
CH_4O	32.026215	[20, 21]	Methanol
2x H_2O	36.021130	[20]	Combination
C_2H_2O	42.010565	[14, 17]	Hydroxypyruvic acid/ -H2O
C_3H_6	42.04695	[16]	Repeated (de-)methylation
CHNO	43.005814	[14]	Carbamoyl- or isocyanide transfer
CO_2	43.989830	[16, 19 - 21, a.o.]	Carbon dioxide/ Carboxyl group
C_2H_4O	44.026215	[15, 16]	Acetaldehyde analogon
C_3H_2O	54.010565	[17]	Propynal analogon
C_2O_2	55.98983	[14]	Glyoxylic acid/ -H2O
C_4H_8	56.0626	[16]	Repeated (de-)methylation
CO_2 + H_2O	62.000395	[19 - 21]	Combination
HNO_3	62.995617	[16]	Nitrate
SO_2	63.961902	[22]	Sulfur dioxide
C_4H_4O	68.026215	[15, 21]	Vinyl Ketene
$C_3H_2O_2$	70.005480	[17]	Propiolic acid analogon
CO_2 + CO	71.984745	[19]	Combination
$C_2H_3NO_2$	73.016379	[14]	Tryptophanase
CO_2 + CH_4O	76.016045	[20]	Combination
SO_3	79.956817	[22]	Sulfur trioxide
H_2SO_3	81.972467	[22]	Sulfurous acid
2x CO_2	87.979660	[16, 19 - 21]	Combination
2x CO_2 + H_2O	105.990225	[19 - 21]	Combination

Table S-6 continued.

Formula	Exact mass difference	Reference(s)	Explanation
$CO_2 + SO_2$	107.951732	[22]	Combination
2x CO_2 + CO	115.974575	[19]	Combination
2x $CO_2 + CH_4O$	120.005875	[20]	Combination
$CO_2 + SO_3$	123.946647	[22]	Combination
2x CO_2 + 2 H_2O	124.000790	[19]	Combination
3x CO_2	131.969490	[19, 20]	Combination
2x $CO_2 + H_2O$ + CO	133.985140	[19]	Combination
3x $CO_2 + CH_4$	148.000790	[19]	Combination
3x $CO_2 + H_2O$	149.980055	[19, 20]	Combination
$C_7H_6O_4$	154.026610	[17]	Dihydroxyl-benzoic acid analogon
3x $CO_2 + CH_4O$	163.995705	[20]	Combination
3x CO_2 + 2 H_2O	167.990620	[19]	Combination
4x CO_2	175.959320	[19]	Combination
3x $CO_2 + H_2O$ + CO	177.974970	[19]	Combination
4x $CO_2 + CH_4$	191.990620	[19]	Combination
4x $CO_2 + H_2O$	193.969885	[19]	Combination

Table S-7. List of all 50+5 Δm features extracted from the reference compound dataset covering several types of aromatic structures (**Figure S-1**). Eight non-indicative Δm's often found in DOM (**Table S-6**) are marked with [DOM]. Five Δm's were added without detection in the tandem MS data of the reference compounds to enable their search in the DOM data (thus the final number of 55). They are indicated by [ADD] and included the neutral loss analogs of precursor ions of compounds #1, #4, #8 and #10, and the common product ion of compounds #12 and #13 (originating from a sugar loss: neutral molecular formula $C_6H_{10}O_5$) used for MS^3 experiments. Contribution of MS^3 data is marked with an asterisk (*) at the compound ID. Compound identifiers are put in brackets if the Δm feature was detected below 1% relative intensity (based on base peak) across three NCE levels. Δm's that contributed only below <1% were only taken into account if detected for more than one compound. Occurrence refers to matches across 159 precursor ions investigated. Eq., equivalent; Comb., combination; pred. predicted by SIRIUS.[13]

Formula	Exact Δm	Compound ID	Explanation
$CH_3^{\bullet}$	15.02347	1, 4, 5, 6, 6*	Methyl radical, loss from radical ion on (6)
H_2O	18.01056	(2), 10, 13*, (14)	Water [DOM]
CO	27.99491	(4), 6*, (8), 12*, 13*	Formyl transf./ Carbon Monooxide [DOM]
C_2H_4	28.03130	4, 5	β-oxidation/ fatty acid synthesis [DOM]
C_2H_2O	42.01056	(2), (4), 6*, 10	Hydroxypyruvic acid/ -H_2O [DOM]
CO_2	43.98983	1, 2, 3, (7), 8, 10, 11, 12*, 13*	Carbon dioxide/ Carboxyl group [DOM]
$CH_2O_2^{\bullet}$	44.99765	(2), (8)	Formic acid equivalent, radical
CH_2O_2	46.00548	(6*), 13, (13*)	Formic acid equivalent
C_3H_6O	58.04186	10	Acetone eq.; comb. C2H2O (ethenone) + CH4 (pred.)
$C_2H_4O_2^{\bullet}$	59.01330	1, (10)	Acetic acid eq., radical
CH_2O_3	62.00039	10, 13*	Comb., CO_2 + H_2O [DOM]
C_2O_3	71.98474	(1), 8, (10), 12*, 13*	Comb., CO_2 + CO [DOM], Carbon Suboxide
$C_4H_4O_2$	84.02113	10	Combination, C3O2 (carbon suboxide) + CH4 (pred.)
$C_3H_2O_3$	86.00039	(1), 10	Combination, C3O2 (carbon suboxide) + H2O (pred.)
$C_2H_2O_4$	89.99531	(10), 13*	Oxalic acid equivalent
C_3O_4	99.97966	8	Comb., CO_2 + 2x CO
$C_4H_6O_3^{\bullet}$	101.02387	10	Radical loss from ion, not matched
$C_4H_6O_3$	102.03169	10	Comb., C4H4O2 + H2O (pred.)
$C_4H_8O_3$	104.04734	14	Hydroxybutyric acid equivalent
$C_6H_4O_2$	108.02113	12*, 13*	Benzoquinone equivalent
$C_6H_6O_2$	110.03678	10	Benzenediol eq.; comb., C3O2 + CH4 + C2H2 (pred.)
$C_4H_2O_4$	113.99531	(8), (10)	Butynedioic acid equivalent
C_3O_5	115.97457	8	Comb., 2x CO_2 + CO [DOM]
$C_4H_8O_4$	120.04226	13	Tetrose equivalent
$C_7H_6O_2$	122.03678	10, 12*, 13*	Loss from flavonols; Comb. on (10): C3O2 + C4H6 (pred.)
$C_7H_8O_2$	124.05243	10, Precursor (5)	3-Methoxyphenol (m-Guaiacol) unit
$C_6H_6O_3$	126.03169	(10), 11, 14	Phloroglucinol unit
$C_5H_4O_4$	128.01096	10	Comb., C3H4O2 + C2O2 (pred.)
$C_7H_6O_3$	138.03169	10, 11, (13*)	Comb., C6H6O2 + CO (pred.)
$C_8H_{10}O_2$	138.06808	Precursor (4)	[ADD] Creosol unit
$C_6H_{10}O_4$	146.05791	14	Sugar unit
$C_6H_{12}O_4^{\bullet}$	147.06573	14	Sugar unit, radical form
$C_8H_6O_3$	150.03169	12*, 13*	Loss from flavonols

Table S-7 continued.

Formula	Exact Δm	Compound ID	Explanation
**$C_7H_4O_4$**	152.01096	9, 11	Incomplete gallic acid unit; H2O retained
**$C_9H_6O_3$**	162.03169	7	Incomplete caffeoyl unit; H2O retained
**$C_6H_{10}O_5$**	162.05282	12, 13	Sugar unit
**$C_6H_{12}O_5^{\bullet}$**	163.06065	(12), 13	Sugar unit, radical form
**$C_9H_8O_3$**	164.04734	9, 10, Precursor (2)	p-coumaric ac.; Comb. on (10): C7H6O3 + C2H2 (pred.)
**$C_8H_8O_4$**	168.04226	Precursor (1)	[ADD] Vanillic acid unit
**$C_7H_6O_5$**	170.02152	9, 11, Precursor (3)	Gallic acid unit
**$C_7H_{10}O_5$**	174.05282	7, (14)	Quinic ac. (7)
**$C_8H_4O_5$**	180.00587	12*, 13*	Loss from flavonols
**$C_9H_8O_4$**	180.04226	(7), 10	Caffeic ac.; Comb. on (10): C7H8O2 + 2x CO (pred.)
**$C_8H_6O_5$**	182.02152	11	Comb., C6H6O3 (e.g., Phloroglucinol) + C2O2 (pred.)
**$C_9H_{10}O_4$**	182.05791	(7), (9)	Comb. on (9): Coumaryl + 2x H2O (pred.)
**$C_7H_8O_6$**	188.03209	(9), 11	Comb., C7H6O5 (e.g., Gallic acid) + H2O (pred.)
**$C_9H_6O_5$**	194.02152	12*, 13*	Loss from flavonols
**$C_{13}H_{12}O_6$**	264.06339	11	Degrad. Catechin C ring after loss A or B-ring
**$C_{13}H_{16}O_7$**	284.08960	(13), 14	Not matched
**$C_{15}H_{12}O_6$**	288.06339	11	Loss of Catechin, gallic ac. remains
**$C_{15}H_{14}O_6$**	290.07904	Precursor (10)	[ADD] Catechin unit
**$C_{14}H_6O_8$**	302.00627	Precursor (8)	[ADD] Ellagic acid unit
**$C_{15}H_{10}O_7$**	302.04265	Precursor (12*, 13*)	[ADD] Flavonol subunit
**$C_{16}H_{12}O_7$**	316.05830	9	Remaining coumaryl subunit after gallic acid loss
**$C_{18}H_{14}O_8$**	358.06887	9	Remaining sugar core after coumaryl/ galloyl loss

Table S-8. Properties of IPIMs (isolated precursor ion mixtures) at four nominal masses ("m/z") and different collision energies ("NCE", first row) and statistical correlation of both factors with these properties ("p-value" two columns to the right; p-value <0.05, significant). Correlations with nominal mass only included the data from one NCE 0 level (non-fragmented) except the number of fragments (row "Products$^{(NCE\ 25)}$"; determined at NCE 25); correlations with NCE level include all NCE levels across the four IPIMs. Data shows averages from duplicate measurements except for NCE 0. Blue and red indicate positive/ negative correlation. Lighter colors (blue, red) or grey indicate significance levels > 0.05. Brackets are put around obvious correlations: the number of atoms in heavier molecules is higher, and precursor ion number sinks upon fragmentation. WA, ion-abundance weighted average.

Property	*m/z* 241				*m/z* 301				*m/z* 361				*m/z* 417				p-value *m/z*	p-value NCE
**NCE**	0	15	20	25	0	15	20	25	0	15	20	25	0	15	20	25		
**Precursor ions**	33	33	29	26	37	38	36	26	43	44	40	29	44	44	43	31	0.026	(0.000)
**Precursor ions assigned**[1]	21	21	21	20	30	31	30	26	34	35	34	26	40	40	40	30	0.043	(0.078)
**Product ions$^{(NCE\ 25)}$**	0	65	131	198	0	87	238	321	0	111	297	390	0	131	401	491	0.002	(0.000)
**H/C_{WA}**	0.91	0.90	0.85	0.81	0.94	0.93	0.90	0.80	0.98	0.97	0.96	0.80	0.99	1.00	1.01	0.97	0.032	0.003
**O/C_{WA}**	0.37	0.35	0.30	0.26	0.45	0.43	0.37	0.29	0.48	0.46	0.41	0.33	0.53	0.50	0.43	0.30	0.038	0.000
**$\#C_{WA}$**	13.1	13.3	13.8	14.3	15.2	15.5	16.1	17.3	17.6	17.8	18.6	20.2	19.6	20.0	21.1	23.1	(0.020)	0.178
**$\#H_{WA}$**	11.7	11.8	11.8	11.7	14.1	14.3	14.5	13.8	17.0	17.2	17.7	15.8	19.3	19.8	21.1	22.1	(0.000)	0.957
**$\#O_{WA}$**	4.54	4.35	3.95	3.56	6.51	6.29	5.77	4.90	8.14	7.92	7.31	6.32	10.0	9.65	8.67	6.78	(0.003)	0.077
**$AI_{mod,WA}$**	0.53	0.54	0.58	0.62	0.47	0.48	0.52	0.60	0.42	0.43	0.45	0.57	0.39	0.39	0.41	0.47	0.010	0.004
**DBE_{WA}**	8.26	8.43	8.98	9.51	9.17	9.38	9.94	11.5	10.1	10.3	10.8	13.3	11.0	11.1	11.5	13.1	0.010	0.004
**$DBE\text{-}O_{WA}$**	3.72	4.08	5.03	5.94	2.66	3.08	4.17	6.56	1.97	2.33	3.51	6.97	0.99	1.50	2.87	6.31	0.003	0.000
**$NOSC_{WA}$**	-0.07	-0.11	-0.17	-0.21	0.04	0.00	-0.08	-0.13	0.07	0.03	-0.07	-0.07	0.14	0.07	-0.08	-0.28	0.012	0.000

[1] Assigned; precursor ion with an assigned molecular formula.

Table S-9. Overview of correlations (Pearson's r; red, negative correlation; blue, positive correlation) between key properties of the IPIM (representing the bandwidth of possible isomers behind a given exact precursor ion m/z) at m/z 241 (precursor ions with molecular formula = 20). Shown are descriptors of ionization and fragmentation behavior (i.e., initial intensity (Iabs, initial), fragmentation at different NCE stages ($I_{rel,\ loss}$) and number of matches to non-indicative Δm's reported for DOM (Table S-1) and their relation to the precursor ion's m/z (here, equivalent to mass defect) and molecular formula (numbers of #C, #H and #O atoms, their atomic H/C and O/C ratios, the nominal oxidation state of carbons (NOSC)[24], number of oxygen-corrected double bond equivalents (DBE-O)[25], and the number of CO_2 (0 – 4), H_2O (0 – 2), CO (0 – 1) losses inferred from non-indicative Δm's and their combinations (**Table S-1**). Other molecular indices as double bond equivalent (DBE), aromaticity index (AImod)[26], and the number of CH_2 losses (0 – 4) were tested but showed non-significant (ns) relationships in this analysis. Explanation of p-value notation: $p > 0.05$, "ns"; $0.05 \geq p > 0.01$, "*"; $0.01 \geq p > 0.001$, "**"; $p \leq 0.001$, "***".

	$I_{rel, loss, NCE 15}$	$I_{rel, loss, NCE 20}$	$I_{rel, loss, NCE 25}$	$I_{abs, initial}$	Matches
m/z	-0.59 **	-0.64 **	-0.68 **	-0.18 ns	-0.29 ns
**# C**	-0.63 **	-0.74 ***	-0.78 ***	-0.05 ns	-0.39 ns
**# H**	-0.5 *	-0.54 *	-0.59 **	-0.22 ns	-0.29 ns
**# O**	0.63 **	0.77 ***	0.77 ***	0.28 ns	0.62 **
**H/C**	-0.33 ns	-0.32 ns	-0.35 ns	-0.2 ns	-0.15 ns
**O/C**	0.68 **	0.78 ***	0.74 ***	0.16 ns	0.53 *
**NOSC**	0.61 **	0.66 **	0.69 ***	0.14 ns	0.35 ns
**DBE-O**	-0.29 ns	-0.4 ns	-0.36 ns	-0.01 ns	-0.31 ns
**n CO_2**	0.52 *	0.65 **	0.64 **	0.53 *	0.84 ***
**n H_2O**	0.33 ns	0.51 *	0.52 *	0.54 *	0.86 ***
**n CO**	-0.03 ns	0.12 ns	0.21 ns	0.69 ***	0.74 ***
**$I_{rel, loss, NCE 15}$**		0.94 ***	0.73 ***	0.06 ns	0.32 ns
**$I_{rel, loss, NCE 20}$**			0.88 ***	0.18 ns	0.5 *
**$I_{rel, loss, NCE 25}$**				0.25 ns	0.52 *
**$I_{abs, initial}$**					0.81 ***

Table S-10. Overview of correlations (Pearson's r; red, negative correlation; blue, positive correlation) between key properties of the IPIM (representing the bandwidth of possible isomers behind a given exact precursor ion m/z) at *m/z* 301 (precursor ions with molecular formula = 27). Shown are descriptors of ionization and fragmentation behavior (i.e., initial intensity ($I_{abs, initial}$), fragmentation at different NCE stages ($I_{rel, loss}$) and number of matches to non-indicative Δm's reported for DOM (**Table S-1**) and their relation to the precursor ion's *m/z* (here, equivalent to mass defect) and molecular formula (numbers of #C, #H and #O atoms, their atomic H/C and O/C ratios, the nominal oxidation state of carbons (NOSC)[23], number of oxygen-corrected double bond equivalents (DBE-O)[24], and the number of CO_2 (0 – 4), H_2O (0 – 2), CO (0 – 1) losses inferred from non-indicative Δm's and their combinations (**Table S-1**). Other molecular indices as double bond equivalent (DBE), aromaticity index (AI_{mod})[25], and the number of CH_2 losses (0 – 4) were tested but showed non-significant (ns) relationships in this analysis. Explanation of p-value notation: $p > 0.05$, "ns"; $0.05 \geq p > 0.01$, "*"; $0.01 \geq p > 0.001$, "**"; $p \leq 0.001$, "***".

	$I_{rel, loss, NCE 15}$	$I_{rel, loss, NCE 20}$	$I_{rel, loss, NCE 25}$	$I_{abs, initial}$	**Matches**
m/z	-0.47 *	-0.66 ***	-0.43 *	-0.12 ns	-0.22 ns
**# C**	-0.59 **	-0.85 ***	-0.87 ***	-0.06 ns	-0.35 ns
**# H**	-0.35 ns	-0.53 **	-0.3 ns	-0.12 ns	-0.17 ns
**# O**	0.64 ***	0.9 ***	0.83 ***	0.24 ns	0.54 **
**H/C**	-0.12 ns	-0.18 ns	0.1 ns	-0.08 ns	0.01 ns
**O/C**	0.64 ***	0.87 ***	0.77 ***	0.11 ns	0.45 *
**NOSC**	0.48 *	0.73 ***	0.56 **	0.03 ns	0.21 ns
**DBE-O**	-0.49 **	-0.64 ***	-0.78 ***	-0.1 ns	-0.41 *
**n CO_2**	0.59 **	0.62 ***	0.46 *	0.54 **	0.85 ***
**n H_2O**	0.36 ns	0.48 *	0.49 **	0.5 **	0.71 ***
**n CO**	-0.2 ns	-0.14 ns	-0.12 ns	0.44 *	0.21 ns
$I_{rel, loss, NCE 15}$		0.83 ***	0.55 **	0.15 ns	0.52 **
$I_{rel, loss, NCE 20}$			0.84 ***	0.25 ns	0.56 **
$I_{rel, loss, NCE 25}$				0.26 ns	0.47 *
$I_{abs, initial}$					0.81 ***

Table S-11. Overview of correlations (Pearson's r; red, negative correlation; blue, positive correlation) between key properties of the IPIM (representing the bandwidth of possible isomers behind a given exact precursor ion m/z) at *m/z* 361 (precursor ions with molecular formula = 30). Shown are descriptors of ionization and fragmentation behavior (i.e., initial intensity ($I_{abs,\ initial}$), fragmentation at different NCE stages ($I_{rel,\ loss}$) and number of matches to non-indicative Δm's reported for DOM (**Table S-1**) and their relation to the precursor ion's *m/z* (here, equivalent to mass defect) and molecular formula (numbers of #C, #H and #O atoms, their atomic H/C and O/C ratios, the nominal oxidation state of carbons (NOSC)[23], number of oxygen-corrected double bond equivalents (DBE-O)[24], and the number of CO_2 (0 – 4), H_2O (0 – 2), CO (0 – 1) losses inferred from non-indicative Δm's and their combinations (**Table S-1**). Other molecular indices as double bond equivalent (DBE), aromaticity index (AI_{mod})[25], and the number of CH_2 losses (0 – 4) were tested but showed non-significant (ns) relationships in this analysis. Explanation of p-value notation: $p > 0.05$, "ns"; $0.05 \geq p > 0.01$, "*"; $0.01 \geq p > 0.001$, "**"; $p \leq 0.001$, "***".

	$I_{rel,\ loss,\ NCE\ 15}$	$I_{rel,\ loss,\ NCE\ 20}$	$I_{rel,\ loss,\ NCE\ 25}$	$I_{abs,\ initial}$	**Matches**
m/z	-0.58 ***	-0.6 ***	-0.3 ns	-0.1 ns	-0.24 ns
**# C**	-0.76 ***	-0.88 ***	-0.85 ***	-0.01 ns	-0.24 ns
**# H**	-0.49 **	-0.47 **	-0.13 ns	-0.11 ns	-0.21 ns
**# O**	0.84 ***	0.92 ***	0.81 ***	0.16 ns	0.4 *
**H/C**	-0.17 ns	-0.08 ns	0.25 ns	-0.12 ns	-0.1 ns
**O/C**	0.85 ***	0.9 ***	0.75 ***	0.03 ns	0.28 ns
**NOSC**	0.76 ***	0.74 ***	0.43 *	0.03 ns	0.2 ns
**DBE-O**	-0.51 **	-0.64 ***	-0.8 ***	-0.02 ns	-0.21 ns
**n CO_2**	0.45 *	0.51 **	0.43 *	0.71 ***	0.83 ***
**n H_2O**	0.26 ns	0.42 *	0.46 *	0.62 ***	0.79 ***
**n CO**	-0.01 ns	0.07 ns	0.09 ns	0.63 ***	0.6 ***
$I_{rel,\ loss,\ NCE\ 15}$		0.92 ***	0.66 ***	0.05 ns	0.28 ns
$I_{rel,\ loss,\ NCE\ 20}$			0.85 ***	0.21 ns	0.45 *
$I_{rel,\ loss,\ NCE\ 25}$				0.26 ns	0.44 *
$I_{abs,\ initial}$					0.92 ***

Table S-12. Overview of correlations (Pearson's r; red, negative correlation; blue, positive correlation) between key properties of the IPIM (representing the bandwidth of possible isomers behind a given exact precursor ion m/z) at *m/z* 417 (precursor ions with molecular formula = 34). Shown are descriptors of ionization and fragmentation behavior (i.e., initial intensity ($I_{abs,\ initial}$), fragmentation at different NCE stages ($I_{rel,\ loss}$) and number of matches to non-indicative Δm's reported for DOM (Table S-1) and their relation to the precursor ion's *m/z* (here, equivalent to mass defect) and molecular formula (numbers of #C, #H and #O atoms, their atomic H/C and O/C ratios, the nominal oxidation state of carbons (NOSC)[23], number of oxygen-corrected double bond equivalents (DBE-O)[24], and the number of CO_2 (0 – 4), H_2O (0 – 2), CO (0 – 1) and $C_7H_6O_4$ (0 – 1)[18] losses inferred from non-indicative Δm's and their combinations (**Table S-1**). Other molecular indices as double bond equivalent (DBE), aromaticity index (AI_{mod})[25], and the number of CH_2 losses (0 – 4) were tested but showed non-significant (ns) relationships in this analysis. Explanation of p-value notation: $p > 0.05$, "ns"; $0.05 \geq p > 0.01$, "*"; $0.01 \geq p > 0.001$, "**"; $p \leq 0.001$, "***".

	$I_{rel,\ loss,\ NCE\ 15}$	$I_{rel,\ loss,\ NCE\ 20}$	$I_{rel,\ loss,\ NCE\ 25}$	$I_{abs,\ initial}$	Matches
m/z	-0.58 ***	-0.67 ***	-0.38 *	-0.2 ns	-0.36 *
**# C**	-0.64 ***	-0.79 ***	-0.78 ***	-0.19 ns	-0.43 *
**# H**	-0.49 **	-0.56 ***	-0.27 ns	-0.18 ns	-0.31 ns
**# O**	0.79 ***	0.88 ***	0.82 ***	0.37 *	0.63 ***
**H/C**	-0.23 ns	-0.24 ns	0.05 ns	-0.1 ns	-0.13 ns
**O/C**	0.8 ***	0.86 ***	0.75 ***	0.28 ns	0.55 ***
**NOSC**	0.67 ***	0.77 ***	0.53 **	0.19 ns	0.4 *
**DBE-O**	-0.42 *	-0.49 **	-0.65 ***	-0.18 ns	-0.35 *
**n CO_2**	0.72 ***	0.74 ***	0.61 ***	0.74 ***	0.89 ***
**n H_2O**	0.51 **	0.57 ***	0.56 ***	0.54 **	0.67 ***
**n CO**	0.13 ns	0.21 ns	0.18 ns	0.57 ***	0.53 **
**n $C_7H_6O_4$**	0.22 ns	0.26 ns	0.2 ns	0.84 ***	0.7 ***
$I_{rel,\ loss,\ NCE\ 15}$		0.89 ***	0.54 ***	0.44 *	0.69 ***
$I_{rel,\ loss,\ NCE\ 20}$			0.76 ***	0.46 **	0.69 ***
$I_{rel,\ loss,\ NCE\ 25}$				0.32 ns	0.52 **
$I_{abs,\ initial}$					0.92 ***

Table S-13. Lists of Δm values used for analysing matching patterns in Van Krevelen space.

**List of Δm's**	**Proposed specificity in DOM (Tables S-14, S-15)**	**Δm members and counting rule**	**Δm cluster (Table S-14) or Δm list (Tables S-6 or S-7)**
CO_2 units (up to four)	General, carboxylic acids and derivatives	If-rule: 4 if matched to $4CO_2$, $4CO_2+CH_4$, or $4CO_2+H_2O$; 3 if matched to $3CO_2$, $3CO_2+CH_4$, $3CO_2+H2O$, $3CO_2+H_2O+CO$, $3CO_2+CH_4O$ or $3CO_2+2H_2O$; 2 if matched to $2CO_2$, $2CO_2+H_2O$, $2CO_2+CO$, $2CO_2+CH_4O$, $2CO_2+2H_2O$ or $2CO_2+H_2O+CO$, 1 if matched to CO_2, CO_2+H_2O, CO_2+CO, CO_2+CH_4O, CO_2+SO_2 or CO_2+SO_3; 0 if matched to none of these	Some in clusters 1 and 6; all of them in Table S-6
CH_2 units (up to four)	General	If-rule: 4 if matched to C_4H_8; 3 if matched to C_3H_6; 2 if matched to C_2H_4; 1 if matched to CH_2; 0 if matched to none of these	C_2H_4 in cluster 7; all of them in Table S-6
CO units (up to 2)	General, benzenoids and derivatives	If-rule: 2 if matched to 2CO; 1 if matched to CO, CO_2+CO, $2CO_2+CO$, $2CO_2+H_2O+CO$ or $3CO_2+H_2O+CO$; 0 if matched to none of these	Cluster 6
$^{\bullet}CH_3$ unit	Benzenoids and ethers	Match to $^{\bullet}CH_3$ loss	Cluster 7; only Table S-7
Polyol eqs.	Organooxygen compounds, especially polyols and glycosides	Sum of matches to Δm's in cluster 2	Cluster 2
Phenylpropanoids and Benzenoids	Shared between phenylpropanoids and polyketides but also benzenoids, but also vinylogous acids in general	Sum of matches to Δm's in clusters 3 and 4	Clusters 3 and 4
Gallate eqs.	Not specific for gallate-containing species (Table S-14) but equivalent to its loss in compounds #9 and and #11 (Figure S-1, Table S-4)	Sum of matches to $C_7H_4O_4$ (gallate removal with water remaining) $C_7H_6O_5$ (gallate removal) and $C_7H_8O_6$ (gallate removal with additional water abstraction)	Part of cluster 4

Table S-14. Matching behavior of precursor ion clusters A – H (color-scaled, see **Table 1** as well) against Δm features derived from reference compounds measured on the same instrument (**Table S-7**). Colors and abbreviations, see below table. "Count" shows the number of compounds showing this feature in the SIRIUS list. Cluster number ("#") indicates groups of Δm's that matched similarly with DOM precursor ions. Δm cluster 5 was omitted here because it did not match with DOM precursor ions. The right side of the table (colored column heads) shows the specificity of each Δm feature for compound classes defined by Classyfire. Numbers indicate the percentage of compounds showing the Δm feature in SIRIUS, associations between clusters and compound classes are highlighted in bold.

											PP+PK			OA+					FA	C6H6+					OOx+						OrgHCy				
															CA+											COx									
Δm	Count[1]	#	A	B	C	D	E	F	G	H	G	FLAV*	L2arP*	VA	G	CA*	MCA	AA*	FA *	G	PH*	PHE*	C6H6*	BPy*	OOx	ROH*	CARB*	ROR*	C=O*	ACR	G	Ox	OxCy	LCT	Py*
C3O5	79	1	70	0	6	0	100	38	100	36	-	-	-	-	-	-	-	-	-	-	-	-	-	-	-	-	-	-	-	-	-	-	-	-	-
C3O4	142	1	70	0	0	13	100	50	100	0	-	45	-	-	-	-	-	-	-	-	-	-	-	55	-	-	-	-	-	-	-	-	-	-	-
C5H4O4	152	1	0	0	0	0	100	50	100	0	-	-	-	-	-	-	-	-	-	-	-	-	-	-	-	-	-	-	-	-	-	-	-	-	-
C4H2O4	142	1	20	0	0	0	88	50	100	0	-	35	-	-	-	-	-	-	-	-	-	-	-	52	-	-	-	-	-	-	-	-	-	-	48
C3H2O3	416	1	0	7	6	25	100	88	100	36	-	-	-	-	-	68	66	-	-	-	-	-	-	-	-	-	-	-	-	-	-	-	-	-	-
CH2O2	1289	1	10	7	0	31	100	88	100	86	-	-	-	-	-	-	-	-	-	-	-	-	-	-	-	-	-	-	-	-	-	-	-	-	-
C2H2O4	331	1	30	17	0	0	100	63	100	79	-	-	-	-	-	-	-	-	-	-	-	-	-	-	-	-	-	-	-	-	-	-	-	-	-
C4H6O3	465	1	0	3	0	0	100	88	50	79	-	-	13	-	-	-	-	-	-	-	-	-	-	-	-	-	-	-	-	-	-	-	-	-	-
C4H8O4	317	2	0	37	0	0	100	25	20	100	-	-	-	-	-	-	-	-	-	-	-	-	-	-	-	**76**	**48**	**16**	-	-	-	42	-	-	-
C4H8O3	243	2	0	33	0	0	100	63	20	93	-	-	-	-	-	-	-	-	-	-	-	48	-	-	-	-	-	-	-	-	-	-	-	-	-
C7H10O5	110	2	0	7	0	0	88	13	0	64	-	-	-	-	-	-	-	-	-	-	-	-	-	-	-	-	-	-	-	-	-	-	-	-	-
C6H10O5	620	2	0	23	0	0	88	13	0	64	-	17	-	-	-	-	-	-	-	-	-	-	-	-	-	**92**	**76**	**80**	-	-	92	77	90	-	-
C6H10O4	289	2	0	20	0	0	88	13	10	64	-	-	-	-	-	-	-	-	-	-	-	-	-	-	-	-	-	-	-	-	-	-	-	-	-
C8H6O3	246	3	20	13	0	75	100	88	20	14	**63**	-	**25**	53	-	-	-	-	-	-	**69**	-	**94**	-	-	-	-	-	25	25	-	-	-	-	-
C9H6O3	155	3	0	3	0	75	50	63	10	0	**81**	**43**	-	-	-	-	-	-	-	-	**76**	-	-	**67**	-	-	-	-	-	-	-	-	-	-	57
C9H8O3	229	3	0	3	0	50	63	100	0	0	**87**	-	**19**	48	-	-	-	-	-	-	**84**	-	**94**	**39**	-	-	-	-	-	-	-	-	-	-	-
C7H6O2	254	3	20	0	0	50	38	75	0	0	-	-	-	-	-	-	-	-	-	-	-	-	-	-	-	-	-	-	-	-	-	-	-	-	-
C6H4O2	63	3	20	0	0	75	63	63	20	0	-	-	-	-	-	-	-	-	-	-	-	-	-	-	-	-	-	-	-	-	-	-	-	-	-
C2H4O2•	621	3	0	0	6	44	63	75	60	0	-	-	-	-	-	-	-	-	-	-	-	**37**	-	-	-	-	-	44	-	-	-	-	-	29	-
C6H6O2	114	3	0	0	0	6	38	75	10	0	-	-	-	-	-	-	-	-	-	-	**53**	-	-	-	-	-	-	-	-	-	-	-	-	-	-
C4H4O2	252	3	0	0	0	25	88	75	20	0	-	-	-	-	-	-	-	-	-	-	-	-	-	-	-	-	-	-	-	-	-	-	-	-	-
C8H10O2	178	3	0	0	6	25	63	88	0	21	-	-	-	-	-	-	-	-	-	-	-	-	-	-	-	-	-	-	-	-	-	-	-	-	-
C7H8O2	179	3	0	3	6	44	63	100	0	43	-	-	-	-	-	-	-	-	-	-	-	-	-	-	-	-	-	-	-	-	-	-	-	-	-
C6H6O3	166	3	0	7	0	0	100	100	30	43	-	-	-	50	-	-	-	-	-	-	-	-	-	-	-	-	-	-	-	-	-	-	-	-	-
C3H6O	539	3	0	0	6	13	88	100	20	64	-	-	**18**	-	-	-	-	-	-	-	-	**58**	**45**	-	-	-	-	70	18	18	-	-	-	-	-

Table S-14. Continued.

Δm	Count[1]	#	A	B	C	D	E	F	G	H	PP+PK			OA+					FA *	C6H6+					OOx+						OrgHCy				
															CA+											COx									
											G	FLAV*	L2arP*	VA	G	CA*	MCA	AA*		G	PH*	PHE*	C6H6*	BPy*	OOx	ROH*	CARB*	ROR*	C=O*	ACR	G	Ox	OxCy	LCT	Py*
C9H10O4	154	4	0	0	0	0	100	25	0	29	-	-	-	-	-	-	-	-	-	-	-	-	-	-	-	-	-	-	-	-	-	-	-	-	-
C8H8O4	129	4	0	0	0	0	100	50	10	21	-	-	-	-	-	-	-	-	-	-	-	-	-	-	-	-	-	-	-	-	-	-	-	-	-
C9H8O4	184	4	0	3	0	6	100	75	10	0	**67**	**26**	-	-	-	-	-	-	-	-	-	-	-	**48**	-	-	-	-	-	-	-	-	-	-	48
C8H6O5	56	4	0	3	0	0	100	25	30	0	-	-	-	-	-	-	-	-	-	-	**90**	-	-	-	-	-	-	-	-	-	-	-	-	-	-
C7H6O5	79	4	0	7	0	0	100	13	30	14	-	-	-	-	-	-	-	-	-	-	**73**	-	-	-	-	-	-	-	-	-	-	-	-	-	-
C7H8O6	39	4	10	10	0	0	88	13	20	14	-	-	-	-	-	-	-	-	-	-	-	-	-	-	-	-	-	-	-	-	-	-	-	-	-
C7H4O4	102	4	0	7	0	13	88	50	50	0	-	-	-	67	-	-	-	-	-	-	**87**	-	-	-	-	-	-	-	-	-	-	-	-	-	-
C7H6O3	142	4	10	3	0	25	100	88	30	0	-	-	-	-	-	-	-	-	-	-	-	-	-	-	-	-	-	-	-	-	-	-	-	-	-
C9H6O5	68	4	20	0	0	19	75	38	20	0	-	-	-	82	-	-	-	-	-	-	**100**	-	-	-	-	-	-	-	-	-	-	-	-	-	-
C8H4O5	35	4	20	0	0	19	63	25	50	0	-	-	-	-	-	-	-	-	-	-	-	-	-	-	-	-	-	-	-	-	-	-	-	-	-
CO2	3368	6	100	73	53	100	100	100	100	100	-	-	-	-	**90**	**78**	-	**19**	-	-	-	-	20	-	**98**	-	-	-	-	-	-	-	-	-	-
H2O	2574	6	70	63	41	81	100	100	100	100	-	-	-	-	-	**49**	-	**25**	20	-	-	-	-	-	-	**45**	-	-	**75**	-	-	-	-	-	-
CH2O3	1282	6	40	50	6	38	100	100	100	100	-	-	-	-	**88**	**78**	**58**	**20**	22	-	-	-	-	-	**98**	-	-	-	**81**	-	-	-	-	-	-
C2O3	592	6	100	3	24	56	100	100	100	86	-	-	-	-	-	-	-	-	-	-	-	-	-	-	-	-	-	-	-	-	-	-	-	26	-
CO	958	6	80	3	53	81	100	88	100	64	-	-	8	-	-	-	-	-	-	86	48	-	-	-	-	-	-	-	-	-	-	-	-	-	-
C2H2O	610	7	0	3	47	63	88	88	40	21	-	-	-	-	18	-	-	-	-	-	**53**	-	-	-	-	-	-	-	-	-	-	-	-	-	-
CH3•	1383	7	0	0	100	81	88	75	50	7	-	-	-	-	-	-	-	-	-	**93**	**53**	**57**	**40**	-	-	-	-	59	-	-	-	-	-	-	-
C2H4	367	7	0	17	88	100	100	88	70	79	-	-	-	-	-	-	-	-	-	-	-	-	-	-	-	-	-	-	-	-	-	-	-	-	-

*classes have been aggregated for visualization, full data can be found in the PANGAEA datasets, see introduction of this document. Classes and abbreviations (G marks general specificity to the class): Dark orange = Phenylpropanoids and polyketides (PP+PK), flavonoids (FLAV), Linear 1,3-diarylpropanoids (L2arP); yellow = Organic acids and derivatives (OA+), Vinylogous acids (VA), Carboxylic acids and derivatives (CA+), Carboxylic acids (CA), Monocarboxylic acids and derivatives (MCA), Amino acids, peptides and analogues (AA); lilac = Lipids and lipid-like molecules, here only encompassing the subclass of Fatty acyls (FA); light blue = Benzenoids (C6H6+), Phenols (PH), Phenol ethers (PHE), Benzene and substituted derivatives (C6H6), Benzopyrans (BPy); green = Organic oxygen compounds (OOx+), Organic oxides (OOx), Organooxygen compounds (COx), Alcohols and polyols (ROH), Carbohydrates and carbohydrate conjugates (CARB), Ethers (ROR), Carbonyl compounds (C=O), Acryloyl compounds (ACR); dark blue = Organoheterocyclic compounds (OrgHCy), Oxanes (Ox), Oxacyclic compounds (OxCy), Lactones (LCT), Pyrans (Py).

Table S-15. Lignin-like precursor ion formulas (after Minor et al., 2014)[27] and their molecular properties and clustering (column "precursor ion cluster") based on Δm matching with tandem MS data of reference compounds (**Table 1 and Table S-14)**. Color coding is given only for visual guidance (yellow – green = min – max). Molecular properties are: m/z, mass to charge ratio; I_{init}, initial ion abundance; HL NCE, Half-life NCE; H/C, Hydrogen-to-Carbon ratio; O/C, Oxygen-to-Carbon-ratio; structural grouping based on Minor et al., 2014 (A; all are "L" = Lignin)[26] and Hawkes et al., 2020 (B; "AR" = Aromatics, "LO" = Low-oxygen unsaturated, "HO" = High-oxygen unsaturated, "AL" = Aliphatics, C = Condensed aromatics).[28] Δm matching vs. 14 reference compounds ("Refs.") and SIRIUS Δm list. Precursor ion clusters (B - H) denote the clusters in **Table 1** and **Table S-14** (color only for visual guidance). Δm clusters refer to the same tables (coverage given in % of Δm's in that cluster). *only detected in SRNOM.

Formula	Sample	m/z	I_{init}	HL NCE	H/C	O/C	Domains		Δm's		Precursor ion Cluster	Association to Δm cluster, % coverage						
							A	B	Refs.	SIRIUS		Cl1	Cl2	Cl3	Cl4	Cl5	Cl6	Cl7
$C_{14}H_{18}O_9S$*	SRNOM	361.0599	-	-	1.29	0.64	L	HO	5	119	B	13	0	0	0	0	80	0
$C_{15}H_{22}O_8S$*	SRNOM	361.0962	-	-	1.47	0.53	L	HO	4	141		13	0	0	0	0	60	0
$C_{17}H_{18}N_2O_7$*	SRNOM	361.1041	-	-	1.06	0.41	L	LO	5	272		13	0	0	0	0	80	0
$C_{20}H_{22}N_2O_8$*	SRNOM	417.1302	-	-	1.10	0.40	L	LO	4	362		0	20	0	0	0	60	0
$C_{18}H_{26}O_{11}$	Soil DOM	417.1401	3086	17.9	1.44	0.61	L	HO	8	193		13	80	0	0	0	60	0
	SRNOM		-	-					7	253		0	80	0	0	0	60	0
$C_{16}H_{14}N_2O_8$*	SRNOM	361.0675	-	-	0.88	0.50	L	AR	5	93	C	0	0	0	0	0	80	67
$C_{14}H_{10}O_4$	SRNOM	241.0506	-	-	0.71	0.29	L	C	14	91	D	25	0	25	0	8	100	100
	Soil DOM		24390	22.8					15	76		38	0	25	0	8	100	100
$C_{24}H_{18}O_7$	Soil DOM	417.0979	1940	23.4	0.75	0.29	L	AR	10	189		0	0	33	10	0	60	67
	SRNOM		-	-	0.75	0.29			19	243		0	0	58	40	0	100	100
$C_{17}H_{14}O_9$	SRNOM	361.0565	-	-	0.82	0.53	L	AR	31	239	E	100	40	33	90	0	100	100
$C_{18}H_{18}O_8$	SRNOM	361.0929	-	-	1.00	0.44	L	LO	44	343		100	100	100	100	8	100	100
	Soil DOM		15177	18.4					35	192		100	100	58	90	0	100	100
$C_{19}H_{22}O_7$	SRNOM	361.1293	-	-	1.16	0.37	L	LO	40	374		100	100	83	80	8	100	100
$C_{20}H_{18}O_{10}$	SRNOM	417.0827	-	-	0.90	0.50	L	LO	42	369		100	100	75	100	17	100	100
	Soil DOM		12407	17.9					35	288		100	100	33	100	8	100	67
$C_{21}H_{22}O_9$	SRNOM	417.1191	-	-	1.05	0.43	L	LO	43	465		100	100	92	100	8	100	100
$C_{22}H_{26}O_8$	SRNOM	417.1555	-	-	1.18	0.36	L	LO	35	466		88	100	67	70	8	100	67
$C_{16}H_{14}O_6$	Soil DOM	301.0717	15815	20.0	0.88	0.38	L	AR	33	182	F	100	0	100	50	0	100	100
	SRNOM		-	-					37	245		100	20	100	70	8	100	100
$C_{17}H_{18}O_5$	SRNOM	301.1081	-	-	1.06	0.29	L	LO	34	281		88	40	100	50	0	100	100
	Soil DOM		7470	20.7					28	203		38	20	100	40	0	100	100
$C_{21}H_{22}O_9$	Soil DOM	417.1191	7774	18.6	1.05	0.43	L	LO	33	326		75	100	58	80	8	100	33
$C_{11}H_{14}O_6$	SRNOM	241.0718	-	-	1.27	0.55	L	HO	20	109	G	100	60	17	0	0	100	67
$C_{17}H_{14}O_9$	Soil DOM	361.0566	20202	17.8	0.82	0.53	L	AR	23	131		100	40	25	40	0	100	100
$C_{19}H_{14}O_{11}$	SRNOM	417.0463	-	-	0.74	0.58	L	AR	21	202		100	0	8	60	0	100	33
	Soil DOM		14002	16.7					20	159		88	0	8	60	0	100	33
$C_{11}H_{14}O_6$	Soil DOM	241.0719	10803	17.5	1.27	0.55	L	HO	14	86	H	63	40	8	0	0	100	33
$C_{12}H_{18}O_5$	SRNOM	241.1081	-	-	1.50	0.42	L	AL	13	122		38	60	8	0	0	100	33
	Soil DOM		5181	19.0					11	94		38	40	8	0	0	80	33

Table S-15. Continued.

Formula	Sample	m/z	I_{init}	HL NCE	H/C	O/C	Domains		Δm's		Precursor ion Cluster	Association to Δm cluster, % coverage						
							A	B	Refs.	SIRIUS		Cl1	Cl2	Cl3	Cl4	Cl5	Cl6	Cl7
**$C_{13}H_{18}O_8$**	Soil DOM	301.0928	9086	16.9	1.38	0.62	L	HO	12	145	H	38	100	0	0	0	80	0
	SRNOM		-	-					15	191		38	100	0	10	0	100	33
**$C_{19}H_{22}O_7$**	Soil DOM	361.1292	11254	18.9	1.16	0.37	L	LO	26	249		63	100	42	30	8	100	67
**$C_{20}H_{26}O_6$**	Soil DOM	361.1656	5695	19.8	1.30	0.30	L	LO	14	224		25	60	25	0	0	100	33
	SRNOM		-	-					26	342		63	100	33	30	8	100	100
**$C_{22}H_{26}O_8$**	Soil DOM	417.1554	5746	19.5	1.18	0.36	L	LO	20	317		38	80	33	20	0	100	33
**$C_{23}H_{30}O_7$**	Soil DOM	417.1918	2396	21.4	1.30	0.30	L	LO	9	285		0	40	17	0	0	80	33
	SRNOM		-	-					21	423		50	100	33	10	0	100	67

Table S-16. S-containing precursor ion formulas in soil porewater DOM. Molecular properties given are: m/z, mass to charge ratio; I_{init}, initial ion abundance; HL NCE, Half-life NCE, collision energy required to decrease ion abundance by 50%; H/C, Hydrogen-to-Carbon ratio; O/C, Oxygen-to-Carbon-ratio; structural grouping based on Minor et al., 2014 (A; "L" = Lignin or carboxyl-rich alicyclic molecules, "T" = Tannin, "CH", Condensed hydrocarbons, "P", Protein-like, "NA", part of no group)[26] and Hawkes et al., 2020 (B; "AR" = Aromatics, "LO" = Low-oxygen unsaturated, "HO" = High-oxygen unsaturated, "AL" = Aliphatics, C = Condensed aromatics).[27] Δm matching is given for reference compound ("Refs.") and SIRIUS-derived Δm lists. The last columns show Δm matching with SIRIUS data: "Δm's with S", percentage of Δm features that contain an S atom; "Δm's mass", percentage of Δm features with mass <100 Da or >100 Da (based on all Δm matches); "Range of loss with S Δm", values indicate the range (min – max) percent of C, H or O (of a precursor ion's molecular formula) lost in a Δm feature containing S. Color coding: yellow – green = min – max.

Formula	m/z	I_{init}	HL NCE	H/C	O/C	Structural gr.		Δm's		Δm's with S [%]	Δm's mass [%]		Range of loss with S Δm [%]		
						A	B	Refs.	SIRIUS		<100Da	>100Da	C	H	O
**C9H6O6S**	240.9813	212	19.1	0.67	0.67	T	AR	0	16	100	63	38	0 - 44	0 - 33	0 - 67
**C13H6O3S**	240.9965	40	22.1	0.46	0.23	NA	C	0	6	50	50	50	0 - 15	0 - 0	0 - 33
**C10H10O5S**	241.0176	200	19.8	1.00	0.50	L	LO	1	54	98	59	41	0 - 70	0 - 60	0 - 80
**C14H10O2S**	241.0328	628	11.3	0.71	0.14	CH	C	0	32	56	63	38	0 - 43	0 - 40	0 - 100
**C10H6O9S**	300.9660	108	17.1	0.60	0.90	NA	AR	1	8	88	25	75	0 - 30	0 - 33	33 - 67
**C11H10O8S**	301.0023	204	18.1	0.91	0.73	T	HO	1	49	90	37	63	0 - 55	0 - 60	0 - 75
**C15H10O5S**	301.0176	336	13.1	0.67	0.33	NA	AR	0	40	100	55	45	0 - 60	0 - 60	0 - 60
**C14H22O5S**	301.1114	372	23.0	1.57	0.36	P	AL	0	78	85	29	71	0 - 57	0 - 82	0 - 80
**C15H26O4S**	301.1479	70		1.73	0.27	P	AL	0	26	96	27	73	13 - 53	23 - 69	0 - 75
**C12H10O11S**	360.9872	89	15.4	0.83	0.92	T	HO	0	9	100	22	78	0 - 17	0 - 40	36 - 55
**C16H10O8S**	361.0023	119	19.6	0.63	0.50	NA	AR	1	45	98	44	56	0 - 50	0 - 60	0 - 63
**C13H14O10S**	361.0234	322	16.8	1.08	0.77	T	HO	2	54	72	30	70	0 - 31	0 - 57	0 - 60
**C14H18O9S**	361.0598	2048	17.2	1.29	0.64	L	HO	3	78	71	41	59	0 - 43	0 - 67	0 - 67
**C15H22O8S**	361.0962	4500	19.4	1.47	0.53	L	HO	3	74	66	50	50	0 - 40	0 - 64	0 - 75
**C16H26O7S**	361.1326	692	22.7	1.63	0.44	P	AL	1	37	59	57	43	0 - 50	8 - 69	0 - 71
**C18H10O10S**	416.9922	76	17.5	0.56	0.56	NA	C	1	24	96	54	46	0 - 39	0 - 40	0 - 50
**C15H14O12S**	417.0134	318	16.6	0.93	0.80	T	HO	2	42	74	36	64	0 - 20	0 - 43	0 - 50
**C19H14O9S**	417.0285	298	17.0	0.74	0.47	L	AR	1	83	94	37	63	0 - 53	0 - 71	0 - 56
**C17H22O10S**	417.0859	1672	19.0	1.29	0.59	L	HO	3	152	51	28	72	0 - 41	0 - 55	0 - 80
**C18H26O9S**	417.1224	1974	20.8	1.44	0.50	L	LO	3	99	61	38	62	0 - 44	0 - 54	0 - 56
**C19H30O8S**	417.1588	944	23.1	1.58	0.42	P	AL	2	43	60	58	42	0 - 42	0 - 60	0 - 50
**C20H34O7S**	417.1951	167	24.5	1.70	0.35	P	AL	0	19	53	63	37	5 - 40	12 - 53	0 - 29
**C24H34O4S**	417.2104	1465		1.42	0.17	NA	LO	0	16	100	50	50	4 - 50	0 - 53	0 - 50

Table S-17. N-containing precursor ion formulas in soil porwater DOM. Molecular properties given are: m/z, mass to charge ratio; I_{init}, initial ion abundance; HL NCE, Half-life NCE, collision energy required to decrease ion abundance by 50%; H/C, Hydrogen-to-Carbon ratio; O/C, Oxygen-to-Carbon-ratio; structural grouping based on Minor et al., 2014 (A; "L" = Lignin or carboxyl-rich alicyclic molecules, "T" = Tannin, "CH", Condensed hydrocarbons, "P", Protein-like, "NA", part of no group)[26] and Hawkes et al., 2020 (B; "AR" = Aromatics, "LO" = Low-oxygen unsaturated, "HO" = High-oxygen unsaturated, "AL" = Aliphatics, C = Condensed aromatics).[27] Δm matching is given for reference compound ("Refs.") and SIRIUS-derived Δm lists. The last columns show Δm matching with SIRIUS data: "Δm's with N", percentage of Δm features that contain N atoms; "Δm's mass", percentage of Δm features with mass <100 Da or >100 Da (based on all Δm matches); "Range of loss with N Δm", values indicate the range (min – max) percent of C, H or O (of a precursor ion's molecular formula) lost in a Δm feature containing N. Color coding: yellow – green = min – max.

Formula	m/z	I_{init}	HL NCE	H/C	O/C	Structural gr.		Δm's		Δm's with N [%]	Δm's mass [%]		Range of loss with N Δm [%]		
						A	B	Refs.	SIRIUS		<100Da	>100Da	C	H	O
**C12H6N2O4**	241.0255	207	22.6	0.50	0.33	NA	C	2	11	73	100	0	0 - 25	0 - 0	0 - 75
**C13H10N2O3**	241.0619	897	23.7	0.77	0.23	CH	C	3	54	91	76	24	0 - 77	0 - 60	0 - 100
**C14H14N2O2**	241.0982	526	24.8	1.00	0.14	CH	AR	2	60	92	68	32	0 - 64	0 - 57	0 - 100
**C15H18N2O**	241.1346	39	25.9	1.20	0.07	CH	LO	0	30	97	60	40	0 - 60	0 - 67	0 - 100
**C13H6N2O7**	301.0102	54	18.8	0.46	0.54	NA	C	1	13	92	85	15	0 - 38	0 - 33	0 - 57
**C10H10N2O9**	301.0311	132	18.1	1.00	0.90	T	HO	2	6	67	67	33	0 - 30	0 - 20	33 - 67
**C14H10N2O6**	301.0464	510	20.7	0.71	0.43	L	C	3	70	91	54	46	0 - 71	0 - 60	0 - 83
**C18H10N2O3**	301.0618	111		0.56	0.17	CH	C	0	11	82	64	36	0 - 50	0 - 40	0 - 33
**C11H14N2O8**	301.0675	128	18.5	1.27	0.73	NA	HO	1	30	90	43	57	0 - 64	0 - 71	13 - 75
**C15H14N2O5**	301.0828	1186	19.4	0.93	0.33	L	AR	4	151	92	48	52	0 - 73	0 - 71	0 - 80
**C19H14N2O2**	301.0981	82		0.74	0.11	CH	C	0	21	90	33	67	0 - 63	0 - 57	0 - 50
**C16H18N2O4**	301.1194	409	22.4	1.13	0.25	CH	LO	2	164	94	40	60	0 - 75	0 - 78	0 - 75
**C17H22N2O3**	301.1559	38	23.4	1.29	0.18	NA	LO	0	111	95	35	65	0 - 71	0 - 82	0 - 67
**C15H10N2O9**	361.0312	352	18.3	0.67	0.60	NA	AR	1	60	95	38	62	0 - 53	0 - 60	0 - 67
**C19H10N2O6**	361.0466	197	17.9	0.53	0.32	NA	C	1	25	92	52	48	0 - 63	0 - 60	0 - 50
**C16H14N2O8**	361.0676	1423	17.4	0.88	0.50	L	AR	4	139	92	35	65	0 - 69	0 - 71	0 - 75
**C20H14N2O5**	361.0829	164		0.70	0.25	CH	C	0	74	93	30	70	0 - 75	0 - 71	0 - 60
**C17H18N2O7**	361.1040	1602	18.1	1.06	0.41	L	LO	4	202	93	29	71	0 - 71	0 - 78	0 - 86
**C21H18N2O4**	361.1193	75		0.86	0.19	CH	AR	0	99	95	15	85	0 - 76	0 - 78	0 - 75
**C18H22N2O6**	361.1404	300	19.4	1.22	0.33	L	LO	1	210	94	26	74	0 - 72	0 - 82	0 - 83
**C17H10N2O11**	417.0210	72	19.4	0.59	0.65	NA	C	1	22	95	64	36	0 - 47	0 - 60	0 - 55
**C18H14N2O10**	417.0575	563	18.3	0.78	0.56	L	AR	4	102	92	37	63	0 - 56	0 - 71	0 - 60
**C22H14N2O7**	417.0726	140		0.64	0.32	NA	C	0	55	96	35	65	0 - 59	0 - 71	0 - 57
**C19H18N2O9**	417.0938	992	18.4	0.95	0.47	L	LO	4	200	94	28	72	0 - 68	0 - 78	0 - 78
**C23H18N2O6**	417.1090	100		0.78	0.26	L	AR	0	118	97	15	85	0 - 74	0 - 78	0 - 67
**C20H22N2O8**	417.1302	535	19.8	1.10	0.40	L	LO	3	264	95	22	78	0 - 70	0 - 82	0 - 88
**C21H26N2O7**	417.1666	103	22.3	1.24	0.33	L	LO	1	253	96	19	81	0 - 71	0 - 85	0 - 86

Table S-18. S-containing precursor ion formulas in SRNOM. Structural grouping based on Minor et al., 2014 (A; "L" = Lignin or carboxyl-rich alicyclic molecules, "T" = Tannin, "CH", Condensed hydrocarbons, "P", Protein-like, "NA", part of no group)[26] and Hawkes et al., 2020 (B; "AR" = Aromatics, "LO" = Low-oxygen unsaturated, "HO" = High-oxygen unsaturated, "AL" = Aliphatics, C = Condensed aromatics).[27] Δm matching is given for reference compound ("Refs.") and SIRIUS-derived Δm lists. "Δm's with S", percentage of SIRIUS Δm features that contain an S atom; "Δm's mass", percentage of Δm features with mass <100 Da or >100 Da (based on all SIRIUS Δm matches); "Range of loss with S Δm", values indicate the range (min – max) percent of C, H or O (of a precursor ion's molecular formula) lost in a SIRIUS Δm feature containing S.

Formula	m/z	H/C	O/C	Structural gr.		Δm's		Δm's with S [%]	Δm's mass [%]		Range of loss with S Δm [%]		
				A	B	Refs.	SIRIUS		<100Da	>100Da	C	H	O
C9H6O6S	240.9813	0.67	0.67	T	AR	1	18	94	61	39	0 - 44	0 - 33	0 - 67
C13H6O3S	240.9965	0.46	0.23	NA	C	0	6	50	50	50	0 - 15	0 - 0	0 - 33
C10H10O5S	241.0176	1.00	0.50	L	LO	1	63	97	57	43	0 - 70	0 - 60	0 - 80
C14H10O2S	241.0329	0.71	0.14	CH	C	0	36	44	53	47	0 - 43	0 - 40	0 - 100
C14H6O6S	300.9811	0.43	0.43	NA	C	2	9	78	89	11	0 - 14	0 - 0	0 - 50
C11H10O8S	301.0023	0.91	0.73	T	HO	2	70	87	40	60	0 - 64	0 - 60	0 - 75
C15H10O5S	301.0176	0.67	0.33	NA	AR	3	56	89	55	45	0 - 67	0 - 60	0 - 80
C19H10O2S	301.0330	0.53	0.11	CH	C	0	34	6	26	74	5 - 11	0 - 0	0 - 50
C16H14O4S	301.0539	0.88	0.25	CH	AR	2	107	84	40	60	0 - 69	0 - 71	0 - 75
C13H18O6S	301.0750	1.38	0.46	L	LO	2	171	78	33	67	0 - 69	0 - 78	0 - 83
C14H22O5S	301.1114	1.57	0.36	P	AL	0	112	79	32	68	0 - 57	0 - 82	0 - 80
C15H26O4S	301.1477	1.73	0.27	P	AL	0	35	94	26	74	13 - 53	23 - 69	0 - 75
C15H6O9S	360.9661	0.40	0.60	NA	C	0	8	100	75	25	0 - 13	0 - 0	0 - 44
C16H10O8S	361.0024	0.63	0.50	NA	AR	2	53	94	45	55	0 - 50	0 - 60	0 - 63
C20H10O5S	361.0176	0.50	0.25	NA	C	1	26	23	50	50	0 - 15	0 - 40	0 - 40
C13H14O10S	361.0233	1.08	0.77	T	HO	3	91	57	32	68	0 - 31	0 - 71	0 - 60
C17H14O7S	361.0388	0.82	0.41	L	AR	3	137	93	39	61	0 - 59	0 - 71	0 - 71
C14H18O9S	361.0599	1.29	0.64	L	HO	5	119	71	44	56	0 - 43	0 - 67	0 - 89
C15H22O8S	361.0962	1.47	0.53	L	HO	4	141	56	39	61	0 - 47	0 - 64	0 - 75
C12H26O10S	361.1177	2.17	0.83	CA	AL	0	3	0	100	0	0 - 0	0 - 0	0 - 0
C16H26O7S	361.1326	1.63	0.44	P	AL	1	90	48	36	64	0 - 50	8 - 69	0 - 86
C17H30O6S	361.1689	1.76	0.35	P	AL	0	33	27	42	58	6 - 47	20 - 60	17 - 50
C18H10O10S	416.9922	0.56	0.56	NA	C	1	33	94	45	55	0 - 44	0 - 60	0 - 60
C22H10O7S	417.0074	0.45	0.32	NA	C	1	11	45	64	36	0 - 14	0 - 40	0 - 29
C15H14O12S	417.0134	0.93	0.80	T	HO	3	53	77	40	60	0 - 27	0 - 43	0 - 50
C19H14O9S	417.0286	0.74	0.47	L	AR	1	114	89	39	61	0 - 53	0 - 71	0 - 67
C30H10OS	417.0382	0.33	0.03	NA	C	1	120	3	26	74	0 - 3	0 - 20	0 - 0
C23H14O6S	417.0440	0.61	0.26	NA	C	0	56	38	32	68	0 - 43	0 - 50	0 - 50
C16H18O11S	417.0495	1.13	0.69	T	HO	4	133	52	33	67	0 - 38	0 - 67	0 - 73
C20H18O8S	417.0646	0.90	0.40	L	LO	0	15	0	53	47	0 - 0	0 - 0	0 - 0
C17H22O8S2	417.0680	1.29	0.47	L	LO	0	11	91	36	64	6 - 71	0 - 41	0 - 25
C17H22O10S	417.0861	1.29	0.59	L	HO	4	179	62	32	68	0 - 47	0 - 64	0 - 80
C21H22O7S	417.1012	1.05	0.33	L	LO	0	179	86	26	74	0 - 57	0 - 73	0 - 71
C18H26O9S	417.1224	1.44	0.50	L	LO	3	184	46	29	71	0 - 44	0 - 62	0 - 89

Table S-18. Continued.

Formula	m/z	H/C	O/C	Structural gr.		Δm's		Δm's with S [%]	Δm's mass [%]		Range of loss with S Δm [%]		
				A	B	Refs.	SIRIUS		<100Da	>100Da	C	H	O
**C22H26O6S**	417.1380	1.18	0.27	L	LO	0	114	96	18	82	0 - 55	0 - 69	0 - 83
**C19H30O8S**	417.1588	1.58	0.42	P	AL	1	95	42	34	66	0 - 42	0 - 60	0 - 63
**C23H30O5S**	417.1744	1.30	0.22	NA	LO	0	63	27	35	65	28 - 56	15 - 62	0 - 50
**C20H34O7S**	417.1952	1.70	0.35	P	AL	0	24	92	22	78	0 - 52	0 - 60	0 - 80

Table S-19. N-containing precursor ion formulas in SRNOM. Structural grouping based on Minor et al., 2014 (A; "L" = Lignin or carboxyl-rich alicyclic molecules, "T" = Tannin, "CH", Condensed hydrocarbons, "P", Protein-like, "NA", part of no group)[26] and Hawkes et al., 2020 (B; "AR" = Aromatics, "LO" = Low-oxygen unsaturated, "HO" = High-oxygen unsaturated, "AL" = Aliphatics, C = Condensed aromatics).[27] Δm matching is given for reference compound ("Refs.") and SIRIUS-derived Δm lists. "Δm's with N", percentage of SIRIUS Δm features that contain N atoms; "Δm's mass", percentage of Δm features with mass <100 Da or >100 Da (based on all SIRIUS Δm matches); "Range of loss with N Δm", values indicate the range (min – max) percent of C, H or O (of a precursor ion's molecular formula) lost in a SIRIUS Δm feature containing N.

Formula	m/z	H/C	O/C	Structural gr.		Δm's		Δm's with N [%]	Δm's mass [%]		Range of loss with N Δm [%]		
				A	B	Refs.	SIRIUS		<100Da	>100Da	C	H	O
C12H6N2O4	241.0255	0.50	0.33	NA	C	2	8	75	100	0	0 - 25	0 - 0	0 - 50
C13H10N2O3	241.0619	0.77	0.23	CH	C	6	63	89	75	25	0 - 77	0 - 60	0 - 100
C14H14N2O2	241.0982	1.00	0.14	CH	AR	2	75	92	68	32	0 - 71	0 - 71	0 - 100
C15H18N2O	241.1346	1.20	0.07	CH	LO	0	40	98	60	40	0 - 60	0 - 67	0 - 100
C16H6N4O3	301.0370	0.38	0.19	NA	C	0	2	0	100	0	0 - 0	0 - 0	0 - 0
C18H10N2O3	301.0618	0.56	0.17	CH	C	1	15	80	67	33	0 - 50	0 - 40	0 - 67
C15H14N2O5	301.0828	0.93	0.33	L	AR	5	165	93	42	58	0 - 73	0 - 71	0 - 100
C19H14N2O2	301.0982	0.74	0.11	CH	C	1	35	83	40	60	0 - 74	0 - 71	0 - 100
C16H18N2O4	301.1193	1.13	0.25	CH	LO	3	218	94	38	62	0 - 75	0 - 78	0 - 100
C9H22N2O9	301.1250	2.44	1.00	NA	AL	0	1	0	100	0	0 - 0	0 - 0	0 - 0
C17H22N2O3	301.1557	1.29	0.18	NA	LO	0	164	95	35	65	0 - 76	0 - 82	0 - 100
C15H10N2O9	361.0310	0.67	0.60	NA	AR	2	16	75	19	81	7 - 60	0 - 60	33 - 67
C19H10N2O6	361.0466	0.53	0.32	NA	C	1	33	91	52	48	0 - 63	0 - 60	0 - 67
C23H10N2O3	361.0620	0.43	0.13	NA	C	0	7	86	100	0	0 - 4	0 - 40	0 - 67
C16H14N2O8	361.0675	0.88	0.50	L	AR	5	93	88	26	74	0 - 69	0 - 71	0 - 75
C20H14N2O5	361.0829	0.70	0.25	CH	C	2	100	92	34	66	0 - 75	0 - 71	0 - 80
C17H18N2O7	361.1041	1.06	0.41	L	LO	5	272	92	34	66	0 - 71	0 - 78	0 - 86
C21H18N2O4	361.1193	0.86	0.19	CH	AR	1	138	96	20	80	0 - 76	0 - 78	0 - 75
C18H22N2O6	361.1405	1.22	0.33	L	LO	3	302	94	26	74	0 - 72	0 - 82	0 - 83
C19H26N2O5	361.1767	1.37	0.26	L	LO	0	225	96	22	78	0 - 74	0 - 85	0 - 100
C21H10N2O8	417.0363	0.48	0.38	NA	C	1	20	75	60	40	0 - 43	0 - 40	0 - 50
C25H10N2O5	417.0521	0.40	0.20	NA	C	0	8	88	88	13	0 - 12	10 - 60	0 - 60
C18H14N2O10	417.0572	0.78	0.56	L	AR	2	5	20	80	20	11 - 11	50 - 50	0 - 0
C22H14N2O7	417.0726	0.64	0.32	NA	C	0	57	93	37	63	0 - 73	0 - 71	0 - 57
C26H14N2O4	417.0881	0.54	0.15	CH	C	0	5	80	60	40	0 - 27	0 - 36	0 - 0
C19H18N2O9	417.0937	0.95	0.47	L	LO	2	151	93	30	70	0 - 74	0 - 78	0 - 78
C23H18N2O6	417.1090	0.78	0.26	L	AR	0	142	96	19	81	0 - 74	0 - 78	0 - 67
C27H18N2O3	417.1247	0.67	0.11	CH	C	0	10	20	20	80	4 - 44	28 - 33	0 - 33
C20H22N2O8	417.1302	1.10	0.40	L	LO	4	362	94	22	78	0 - 75	0 - 82	0 - 88
C24H22N2O5	417.1456	0.92	0.21	CH	AR	0	211	97	11	89	0 - 75	0 - 82	0 - 80
C21H26N2O7	417.1666	1.24	0.33	L	LO	1	369	95	19	81	0 - 76	0 - 85	0 - 86
C25H26N2O4	417.1824	1.04	0.16	CH	LO	0	5	80	40	60	8 - 52	31 - 73	25 - 50

Table S-20. Structural class-correlated Δm features that were matched to CHOS or CHNO precursor ions in DOM. "Count" refers to the number of individual structures available for the correlation; the number shows decimals because individual structure count was divided by the number of MS^2 spectra available. Correlated classes given are the top ones out of maximum fifteen (the original table is available via PANGAEA, see introduction). Structural class names are inherited from the Classyfire ontology and partly shortened (ac., acids; cl., class/ classes; derivs., derivatives; comps., compounds; Met, Methionine; Cys, Cysteine; dip. org. comps., dipolar organic compounds; analg., analogues). Asterisks on class names indicate that this potential precursor ion structure can be excluded based on the molecular formula (for example, intact Guanidines would contain at least three N atoms but most precursor ions analyzed here had only 2 atoms predicted by molecular formula, as in e.g., $C_{20}H_{22}N_2O_8$). Matches in DOM are given as absolute and percent (in brackets, based on number of all CHOS/ CHNO precursor ions per sample).

Δm	Da	Count	Top correlated structural classes	Soil DOM	SR NOM
Δm features correlated with sulfonic acids or sulfonyls				Matched CHOS precursors	
O2S	63.9619	214.16	Sulfonyls; Organosulfonic ac. & derivs.; Organic sulfonic ac. & derivs.; +9 other classes	1 (4.3)	13 (33.3)
H2O2S	65.9775	55.32	Organosulfonic ac. & derivs.; Organic sulfonic ac. & derivs.; Sulfonyls, +1 other class	0 (0)	1 (2.6)
O3S	79.9568	88.86	Organosulfonic ac. & derivs.; Organic sulfonic ac. & derivs.	14 (60.9)	17 (43.6)
H2O3S	81.9724	51.53	Organosulfonic ac. & derivs.; Organic sulfonic ac. & derivs.; Sulfonyls	8 (34.8)	12 (30.8)
CO3S	91.9568	32.81	Organic sulfonic ac. & derivs.; Organosulfonic ac. & derivs.; Sulfonyls	6 (26.1)	8 (20.5)
C2H2O3S	105.9724	32.35	Sulfonyls	13 (56.5)	17 (43.6)
CO4S	107.9517	51.77	Sulfonyls; Organosulfonic ac. & derivs.; Organic sulfonic ac. & derivs.	6 (26.1)	13 (33.3)
C2H4O3S	107.9881	41.58	Sulfonyls	3 (13)	12 (30.8)
Δm features correlated with thiols				Matched CHOS precursors	
CH2S	45.9877	67.53	Alkylthiols; Thiols; *Cys & derivs.	5 (21.7)	11 (28.2)
CH2O2S	77.9775	135.41	Alkylthiols; Thiols; *Cys & derivs.	0 (0)	5 (12.8)
C2H2O2S	89.9775	89.76	Alkylthiols; Thiols; *Cys & derivs.	8 (34.8)	14 (35.9)
Δm features correlated with thioethers, thia fatty acids, and sulfenyl compounds				Matched CHOS precursors	
C2H2OS	73.9826	160.84	Alkylarylthioethers; Aryl thioethers; Thioethers; Sulfenyl comps.; +10 other classes	9 (39.1)	16 (41)
C2H4O2S	91.9932	83.82	Thioethers, Sulfenyl comps.; *Dipeptides	1 (4.3)	6 (15.4)
C2H6OS	78.0139	29.63	Thia fatty ac.; *Met & derivs.; Dialkylthioethers	0 (0)	3 (7.7)
C3H6O2S	106.0088	48.4	Thia fatty ac.; Thioethers; Dialkylthioethers; *Met & derivs.; Sulfenyl comps.	10 (43.5)	13 (33.3)
C4H6O2S	118.0088	36.84	*Met & derivs.; Dialkylthioethers; *Dipeptides	2 (8.7)	0 (0)
C5H8O2S	132.0245	24.82	Thia fatty ac.; Dialkylthioethers	4 (17.4)	9 (23.1)
Δm features correlated with dicarboximides and ureides				Matched CHNO precursors	
CHNO	43.0058	480.31	Organic carbonic ac. & derivs.; N-acyl ureas; Dicarboximides	3 (11.1)	4 (12.5)
C2H2N2O2	86.0116	104.12	N-acyl ureas; Ureides; Dicarboximides	11 (40.7)	11 (34.4)
C2HNO3	86.9956	128.43	Dicarboximides; Barbituric ac. derivs.; Carboxylic ac. imides	0 (0)	4 (12.5)
Δm features correlated with carboximidamides (but also amino acids)				Matched CHNO precursors	
CH2N2	42.0217	181.1	*Guanidines; Carboximidamides; Propargyl-type 1,3-dip. org. comps.; +12 other cl.	13 (48.1)	19 (59.4)
CH4N2O	60.0323	103.67	*Guanidines; Carboximidamides; Propargyl-type 1,3-dip. org. comps.; +11 other cl.	14 (51.9)	11 (34.4)
CH6N2O2	78.0429	30.09	*Guanidines; Carboximidamides	0 (0)	3 (9.4)
Δm features correlated with aralkylamines				Matched CHNO precursors	
CH3N	29.0265	107.2	2-arylethylamines	1 (3.7)	0 (0)
C2H6N	44.0500	58.03	Aralkylamines	3 (11.1)	2 (6.3)
Δm features correlated with amino acids, primary amines and peptides				Matched CHNO precursors	
C2H7NO2	77.0476	43.25	Amino ac. & derivs.; Amino ac., peptides & analg.; Alpha amino ac. & derivs.	0 (0)	1 (3.1)
C3H5NO2	87.0320	337.9	Amino ac.; Alpha amino ac. & derivs.; Amino ac. & derivs.; + 8 other classes	0 (0)	5 (15.6)
C5H10N2O	114.0793	37.09	Pyrrolidinecarboxamides; Proline & derivs.	6 (22.2)	6 (18.8)
C4H8N2O3	132.0534	86.09	Primary amines; Dipeptides; Peptides; Alpha amino ac. amides, + 5 other classes	7 (25.9)	9 (28.1)
C5H12N2O2	132.0898	37.92	Proline & derivs.	5 (18.5)	6 (18.8)
C3H6N2O4	134.0327	7.85	Serine & derivs.	8 (29.6)	7 (21.9)
C5H8N2O3	144.0534	68.76	Dipeptides; N-acyl-alpha amino ac. & derivs.; Peptides; Alpha amino ac. amides; +1 cl.	7 (25.9)	8 (25)
C5H10N2O3	146.0691	70.74	Peptides	4 (14.8)	5 (15.6)
C6H14N2O2	146.1055	51.92	Peptides; Alpha amino ac. amides; N-acyl-alpha amino ac. & derivs.; + 1 other cl.	3 (11.1)	4 (12.5)
C7H14N2O3	174.1004	62.72	Peptides; Alpha amino ac. & derivs.; Amino ac.; Alpha amino ac. amides; + 2 other cl.	3 (11.1)	4 (12.5)
C7H16N2O3	176.1160	19.03	N-acyl-L-alpha-amino ac.	1 (3.7)	3 (9.4)
C8H14N2O3	186.1004	51.1	Proline & derivs.; Pyrrolidine carboxylic ac. & derivs.; Peptides; + 4 other classes	3 (11.1)	4 (12.5)
C10H12N2O3	208.0847	36.38	Dipeptides; Peptides; Alpha amino ac. amides; Phenylalanine & derivs.; + 4 other cl.	4 (14.8)	6 (18.8)
C12H14N2O3	234.1004	51.49	Dipeptides	2 (7.4)	3 (9.4)

Table S-21. Correlations of selected precursor ion properties with scores of PC axes (**Figure 4**; only DOM precursor ions with assigned molecular formula included in the correlation). PC axes 3 and 4 are shown in addition. Correlations are indicated for all precursor ions (n=94) and those detected in each sample (Column "Sets"). For each combination (PC = x, property = y), Pearson's r and significance are given (0.05 ≥ p > 0.01, "*"; 0.01 ≥ p > 0.001, "**"; p ≤ 0.001, "***"). Negative/ positive correlation is indicated also by color (blue, red); non-significant correlations are shown in lighter color or no color if no direction dominated. Matches, matches against the global list of Δm features; Structures, number of hits in natural product and in-silico databases. Correlations (Pearson) between structure hits and specific Δm features across CHO precursor ions in soil porewater DOM and Suwannee River NOM for selected structural classes. "Δm's" and "Hits" show the maximum number of each across precursor ions. "n" indicates number of CHO precursor ions included (>0 hits OR >0 matches).

PC	Var. expl. [%]	Set	IPIM	Mass defect	H/C	O/C	DBE	AI_{MOD}	NOSC	Matches	Structures	Half-life NCE	Ion abund. (NCE0)
#1	34,5	All (n=94)	0.11	-0.11	-0.12	0.08	0.08	0.11	0.09	0.56***	0.56***	-	-
		SRNOM (n=50)	0.13	-0.18	-0.18	0.17	0.11	0.15	0.16	0.57***	0.54***	-	-
		Soil porewater DOM (n=44)	0.10	-0.06	-0.08	0.02	0.05	0.08	0.04	0.53***	0.59***	-0.20	0.70***
#2	12,9	All (n=94)	-0.11	-0.18	-0.57***	-0.52***	0.61***	0.67***	-0.02	-0.18	0.11	-	-
		SRNOM (n=50)	-0.07	-0.20	-0.63***	-0.55***	0.66***	0.72***	0.02	-0.19	0.07	-	-
		Soil porewater DOM (n=44)	-0.17	-0.19	-0.54***	-0.48**	0.55***	0.64***	-0.02	-0.27	0.17	0.53***	0.06
#3	8,8	All (n=94)	0.31**	0.75***	0.42***	-0.52***	0.02	-0.28**	-0.67***	0.59**	0.19	-	-
		SRNOM (n=50)	0.35*	0.72***	0.35*	-0.51***	0.07	-0.22	-0.65***	0.57***	0.26	-	-
		Soil porewater DOM (n=44)	0.27	0.77***	0.47**	-0.52***	-0.03	-0.34*	-0.68***	0.64***	0.22	0.47**	-0.41**
#4	6,2	All (n=94)	0.41***	-0.06	-0.17	0.19	0.25*	0.09	0.26*	0.21*	-0.29**	-	-
		SRNOM (n=50)	0.47***	-0.03	-0.16	0.18	0.27	0.08	0.24	0.27	-0.24	-	-
		Soil porewater DOM (n=44)	0.35*	-0.11	-0.18	0.22	0.23	0.10	0.30*	0.12	-0.35*	-0.16	-0.16

Table S-22. Correlations (Pearson) between structure hits and specific Δm features across CHO precursor ions in soil porewater DOM and Suwannee River NOM for selected structural classes. "Δm's" and "Hits" show the maximum number of each across precursor ions. "n" indicates number of CHO precursor ions included (>0 hits OR >0 matches).

	Soil porewater DOM						Suwannee River NOM					
**Class**	Δm's	Hits	n	R^2	r	p	Δm's	Hits	n	R^2	r	p
**Benzenoids**												
Benzenoids (gen.)	4	727	56	0.34	0.58	**0.000**	4	727	55	0.40	0.63	**0.000**
Benzoic acids	2	32	53	0.03	-0.18	*0.190*	2	32	47	0.02	-0.15	*0.300*
Methoxybenzenes	8	191	43	0.58	0.76	**0.000**	12	191	44	0.62	0.79	**0.000**
Dimethoxybenzenes	6	54	50	0.32	0.57	**0.000**	6	54	46	0.42	0.64	**0.000**
Phenoxy compounds	7	202	42	0.53	0.73	**0.000**	9	202	41	0.52	0.72	**0.000**
Styrenes	4	64	29	0.15	0.38	**0.041**	4	64	30	0.08	0.28	*0.139*
Benzopyrans	9	354	42	0.42	0.65	**0.000**	12	354	39	0.55	0.74	**0.000**
Chromones	6	246	23	0.21	0.46	**0.026**	6	246	24	0.27	0.52	**0.009**
Anisoles	17	399	57	0.54	0.73	**0.000**	21	399	56	0.54	0.74	**0.000**
Phenols	12	621	56	0.48	0.69	**0.000**	14	621	54	0.47	0.69	**0.000**
1-hydroxy-2-unsubstituted benzenoids	15	604	55	0.46	0.68	**0.000**	19	604	53	0.45	0.67	**0.000**
1-hydroxy-4-unsubstituted benzenoids	13	422	46	0.31	0.56	**0.000**	13	422	43	0.30	0.55	**0.000**
Resorcinols	2	66	28	0.19	0.44	**0.020**	2	66	30	0.22	0.47	**0.009**
Methoxyphenols	4	139	41	0.43	0.66	**0.000**	5	139	42	0.39	0.62	**0.000**
**Lipids and lipid-like molecules**												
Eicosanoids	18	5	30	0.00	-0.02	*0.896*	22	5	27	0.05	0.23	*0.259*
Fatty acids and conjugates	13	36	57	0.00	0.04	*0.783*	18	36	50	0.11	0.34	**0.017**
Hydroxy fatty acids	24	21	41	0.00	0.07	*0.662*	35	16	36	0.06	0.24	*0.159*
Long-chain fatty acids	21	23	39	0.00	-0.07	*0.676*	30	10	34	0.00	0.05	*0.794*
**Organic acids and derivatives**												
Carboxylic acids and derivatives	2	474	65	0.02	0.14	*0.278*	2	474	55	0.04	0.19	*0.155*
Methyl esters	2	35	44	0.00	-0.02	*0.884*	2	35	42	0.05	0.21	*0.175*
Carboxylic acids	10	133	62	0.01	0.10	*0.427*	11	133	54	0.05	0.22	*0.107*
Dicarboxylic acids and derivatives	3	357	47	0.00	0.06	*0.668*	3	357	36	0.02	0.15	*0.375*
Monocarboxylic acids and derivatives	3	230	64	0.01	0.10	*0.449*	3	230	55	0.02	0.13	*0.357*
Hydroxy acids and derivatives	1	57	42	0.03	-0.18	*0.254*	1	57	34	0.00	0.05	*0.770*
Vinylogous acids	12	324	48	0.44	0.66	**0.000**	15	324	48	0.41	0.64	**0.000**
**Organoheterocyclic compounds**												
Lactones	4	401	51	0.00	0.04	0.773	4	401	45	0.04	0.20	0.185
Oxanes	28	133	37	0.03	0.18	0.291	29	133	31	0.06	0.25	0.182
Pyrans	4	237	46	0.29	0.54	**0.000**	4	237	44	0.37	0.61	**0.000**

Table S-22. Continued.

	Soil porewater DOM						Suwannee River NOM					
**Class**	Δm's	Hits	n	R^2	r	p	Δm's	Hits	n	R^2	r	p
**Organooxygen compounds**												
Acryloyl compounds	11	48	39	0.17	0.41	**0.009**	13	48	40	0.19	0.44	**0.005**
Alcohols and polyols	18	452	68	0.00	0.06	*0.644*	19	452	58	0.02	0.14	*0.292*
Secondary alcohols	24	303	53	0.00	-0.07	*0.639*	25	303	45	0.00	0.00	*0.984*
Polyols	24	201	50	0.00	-0.03	*0.829*	24	201	44	0.00	-0.02	*0.905*
Carbohydrates and carbohydrate conjugates	28	130	35	0.12	0.34	**0.045**	29	130	30	0.21	0.46	**0.010**
Glycosyl compounds	23	123	26	0.09	0.30	*0.136*	25	123	23	0.13	0.36	*0.094*
Hexoses	14	104	19	0.01	0.10	*0.678*	13	104	16	0.07	0.26	*0.326*
Carbonyl compounds	4	512	65	0.00	-0.04	*0.779*	4	512	56	0.02	0.13	*0.358*
Aryl ketones	4	312	30	0.15	0.38	**0.037**	4	312	33	0.12	0.34	**0.051**
Ethers	7	595	57	0.22	0.47	**0.000**	7	595	52	0.26	0.51	**0.000**
Alkyl aryl ethers	15	508	56	0.58	0.76	**0.000**	18	508	55	0.54	0.73	**0.000**
**Phenylpropanoids and polyketides**												
Phenylpropanoids and polyketides (gen.)	12	308	42	0.39	0.62	**0.000**	13	308	40	0.33	0.57	**0.000**
Cinnamic acids and derivatives	1	37	22	0.07	0.26	*0.242*	1	37	26	0.01	0.12	*0.552*
Linear 1,3-diarylpropanoids	13	51	39	0.55	0.74	**0.000**	15	51	42	0.46	0.68	**0.000**
Flavonoids	2	96	25	0.26	0.51	**0.009**	2	96	24	0.37	0.60	**0.002**
Flavans	1	75	13	0.03	0.17	*0.580*	1	75	13	0.00	0.04	*0.895*
Flavones	2	52	28	0.13	0.36	*0.062*	2	52	30	0.13	0.36	**0.048**
Hydroxyflavonoids	2	79	24	0.25	0.50	**0.014**	2	79	24	0.40	0.64	**0.001**

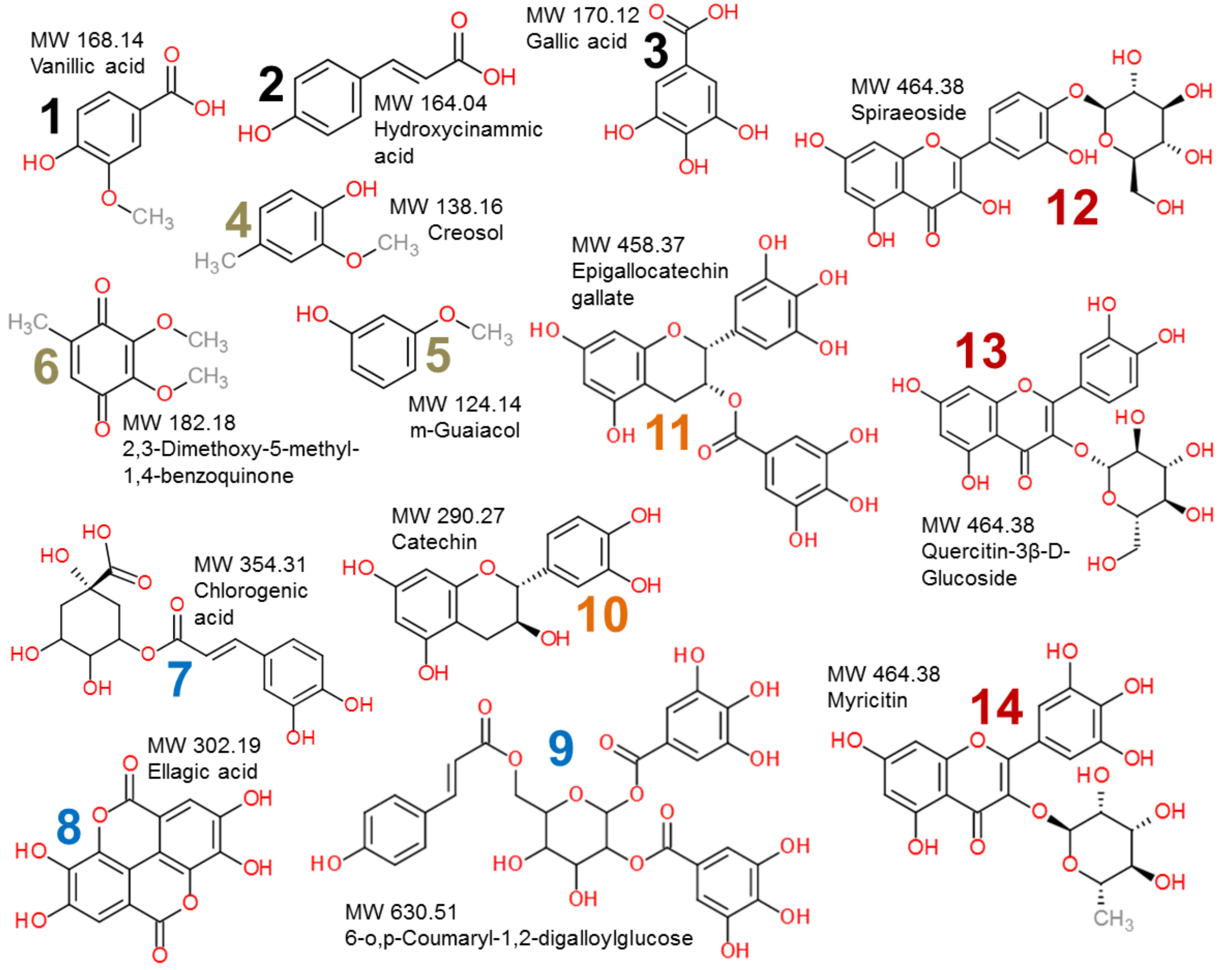

Figure S-1. Overview of reference compounds used in the study (more information in **Table S-2**). Colors of the compound IDs refer to the five groups of compound structures analyzed: Group A (black, #1 – #3), Group B (olive, **#4** – **#6**), Group C (blue, **#7** – **#9**), Group D (orange, **#10**, **#11**), and Group E (red, **#12** – **#14**). Groups A and B contain only one aromatic ring and differ in the presence of functional groups (A: mainly carboxyl, B: mainly methoxy). Group C contains larger structures containing at least two ring structures from fused subunits (**#7**, quinic acid, and caffeic acid; **#8**, two gallic acid monomers; **#9**, coumaric acid, two gallic acid units, and glucose). Group D contains two flavan-3-ol structures, and group E contains three flavonoids with structurally similar but slightly differing flavon-3-ol structures linked to sugars (glycosides).

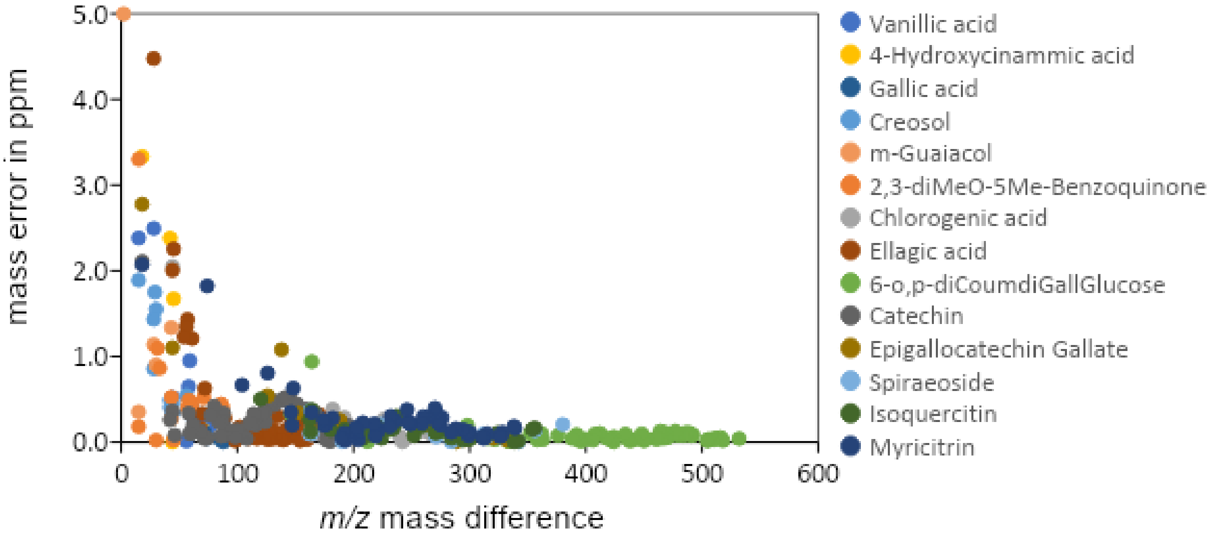

Figure S-2. Error assessment of reference compound Δm's (deviation between measured Δm and exact Δm), as predicted by the precursor ion's molecular formula and its respective product ions. Relative errors become large when the mass difference is small.[19]

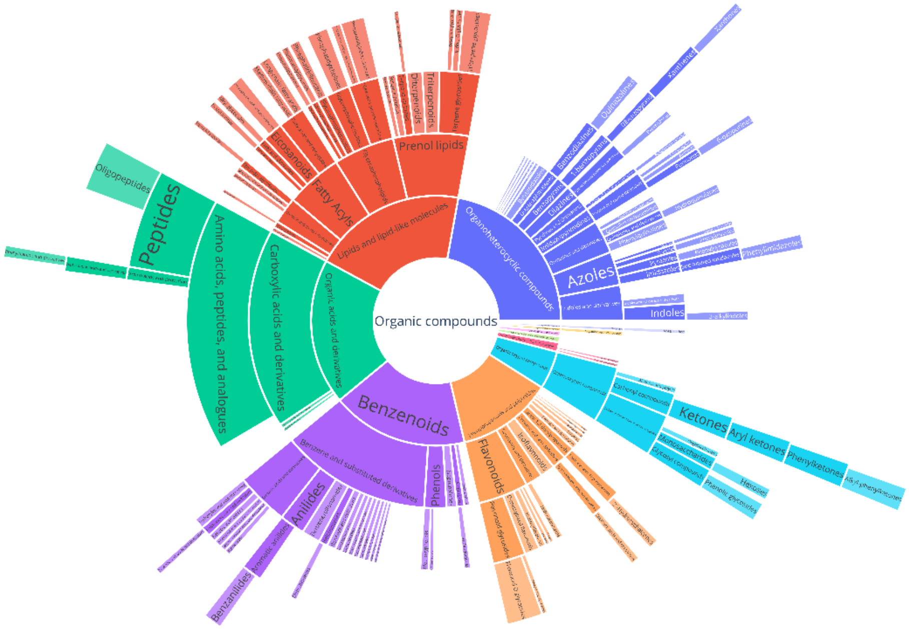

Figure S-3. Distribution of compound classes (classified by Classyfire[29]) in 17994 unique molecular structures annotated by SIRIUS.[13] Reference spectra were collected from from GNPS, MassBank, MoNA, and NIST.[30,31] Only compound classes with at least 50 members were considered for this visualization.

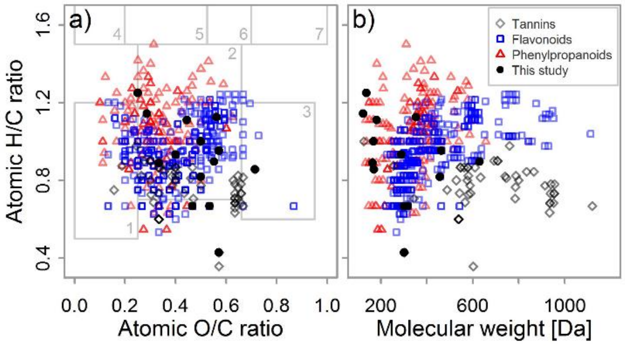

Figure S-4. Distribution of exemplary known structures in chemical space of a) atomic ratios of H and C vs. O and C (Van Krevelen plot) and b) H/C ratio vs. molecular weight. Note that the ordinate is the same in both panels. Three groups of structurally different compound classes from the KEGG database (grey diamonds, tannins, n = 55; blue squares, flavonoids, n=452; and red triangles, phenylpropanoids, n = 185) are depicted for comparison with reference compounds used in this study (black dots, n=14). Grey boxes in panel a indicate structural domains reprinted from Minor et al. (2014)[27]: 1 – Condensed hydrocarbons, 2 – Lignin or carboxyl-rich alicyclic molecules (CRAM), 3 – Tannins, 4 – Lipids, 5 – Protein-like, 6 – Aminosugars, 7 – Carbohydrates.

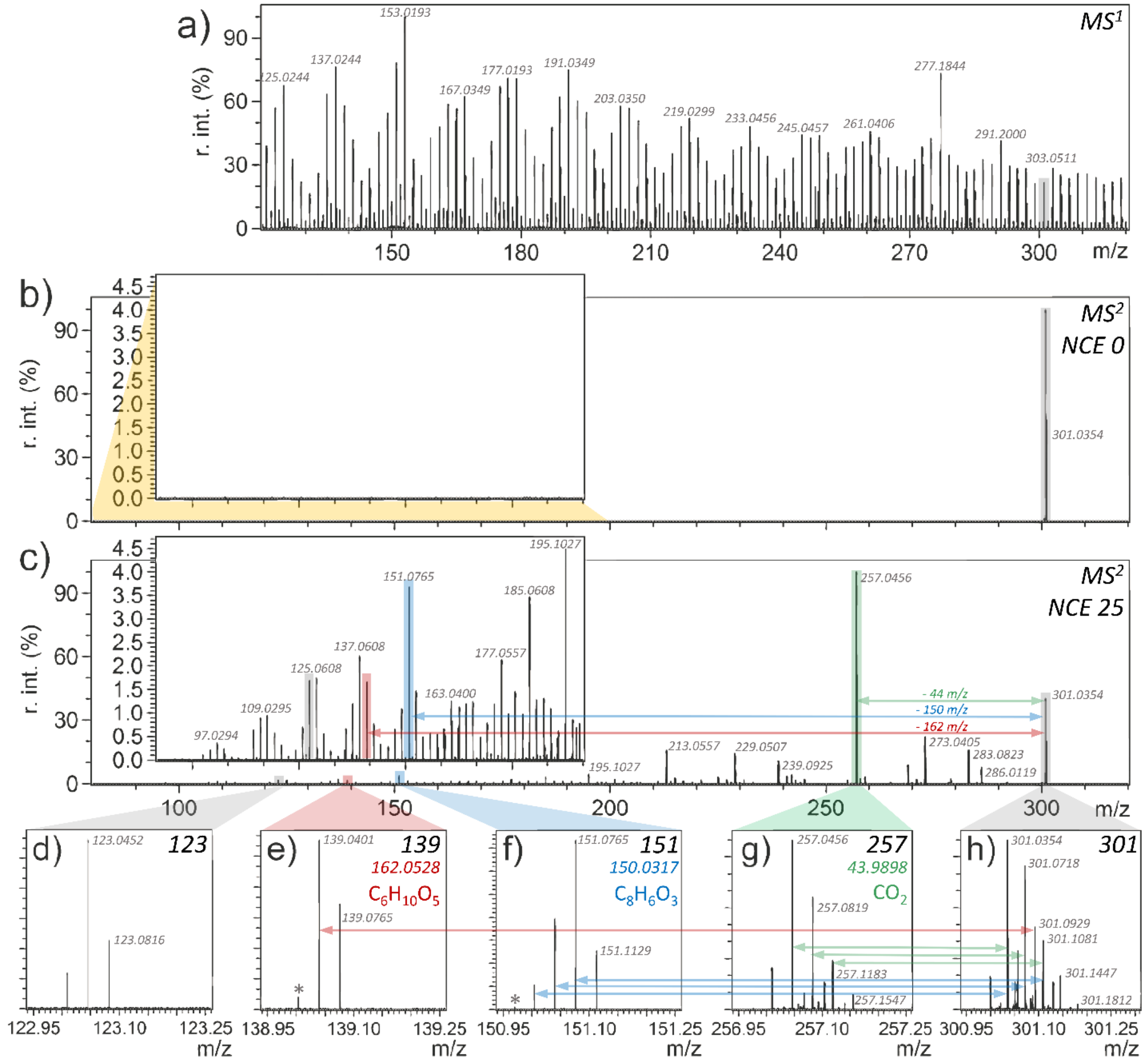

Figure S-5. Orbitrap tandem MS of soil porewater DOM. a) Detail of the initial MS^1 DOM spectrum. The scan range was m/z 120 – 1000. b) Non-fragmented isolated precursor ion mixture (IPIM) @ *m/z* 301 (NCE 0; see detail in panel h). No ions at other m/z values were detected (inset; lower mass range < *m/z* 200, ~20fold enlarged). c) Tandem mass spectrum (MS^2) of IPIM @ *m/z* 301 obtained at NCE 25 and similar inset as in b). Panels d – h) Isobaric detail (exact mass) of four product ion clusters at NCE 25 (d – g) and the initial IPIM @ *m/z* 301 (NCE 0, 44 precursor ions). Four peaks in h) were assigned the molecular formulas $C_{15}H_{10}O_7$, $C_{16}H_{14}O_6$, $C_{13}H_{18}O_8$, and $C_{17}H_{18}O_5$ (in order of increasing exact *m/z*). For those ions, neutral losses are indicated by arrows between isobars (301/ 257, green; 301/ 151, blue, and 301/ 139, red). The respective nominal Δm of 44 (green, panel g), 150 (blue, f) and 162 (red, e) can be assigned to exact Δm's of product ions, such as neutral losses of CO_2 (a common, non-indicative Δm, 3 out of 27 matches to IPIM at *m/z* 301 shown), $C_8H_6O_3$ (an indicative Δm equivalent to a retro-cyclization loss from flavonol-type-molecules, 3/ 4 matches shown) and $C_6H_{10}O_5$ (indicative Δm equivalent to neutral loss of glucose unit, 1/ 2 matches shown). Product ions at m/z 123 (d) had absolute intensities (ion abundances) of 20, 40, and 90, equivalent to signal-to-noise ratios of ~ 7, 13, and 30; the signals were stable in time and detected in repeated measurements. Exemplary peaks that were considered noise are marked with an asterisk (*) in panels e and f.

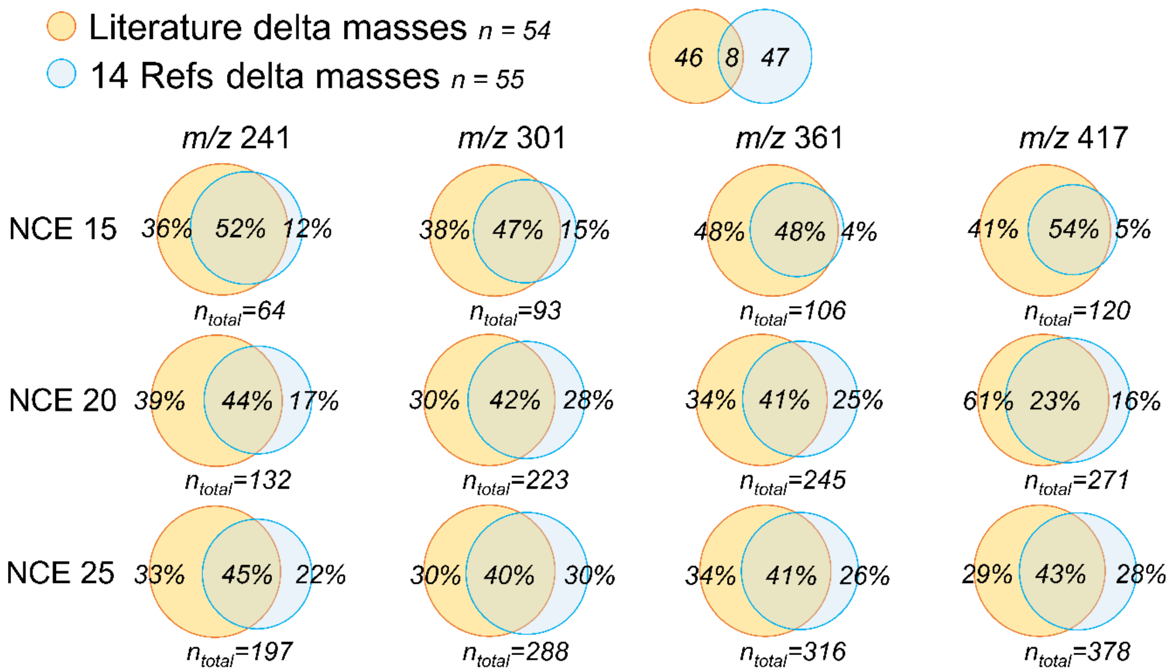

Figure S-6. Comparison of matches to the two short Δm lists (**Table S-6**, **Table S-7**) in relation to nominal mass (m/z) and normalized collision energy (NCE 15 – 25) in soil porewater DOM, shown as Venn diagrams. n_{total} designates the total number of Δm matches at each NCE stage for each IPIM (isolated precursor ion mixture). Percentages indicate the relative amount of unique or shared (overlap) matches between both lists. Note that Venn circles on top designate overlap in terms of the absolute number of Δm's between lists. Not all Δm features were found in DOM.

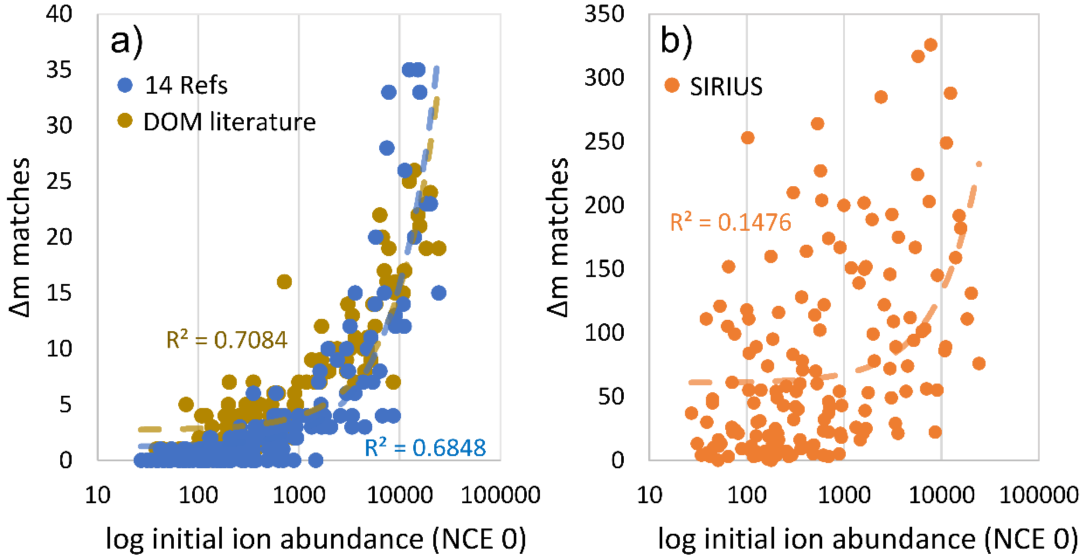

Figure S-7. The number of Δm matches in relation to the log initial ion abundance of the precursor ion in soil porewater DOM; matching against **a)** lists of literature-known DOM Δm features (brown, **Table S-6**), reference compound-derived Δm's ("14 Refs", blue, **Table S-7**, including eight shared Δm features present also in **Table S-6**), and **b)** SIRIUS list of Δm features. Note the different scale in matching between panels a and b. All precursor ions across the four IPIMs (n=159) are shown. Regression curves are linear fits (note log scale). In contrast, measures of fragmentation sensitivity were a poor predictor of the number of matches (**Figure S-8**).

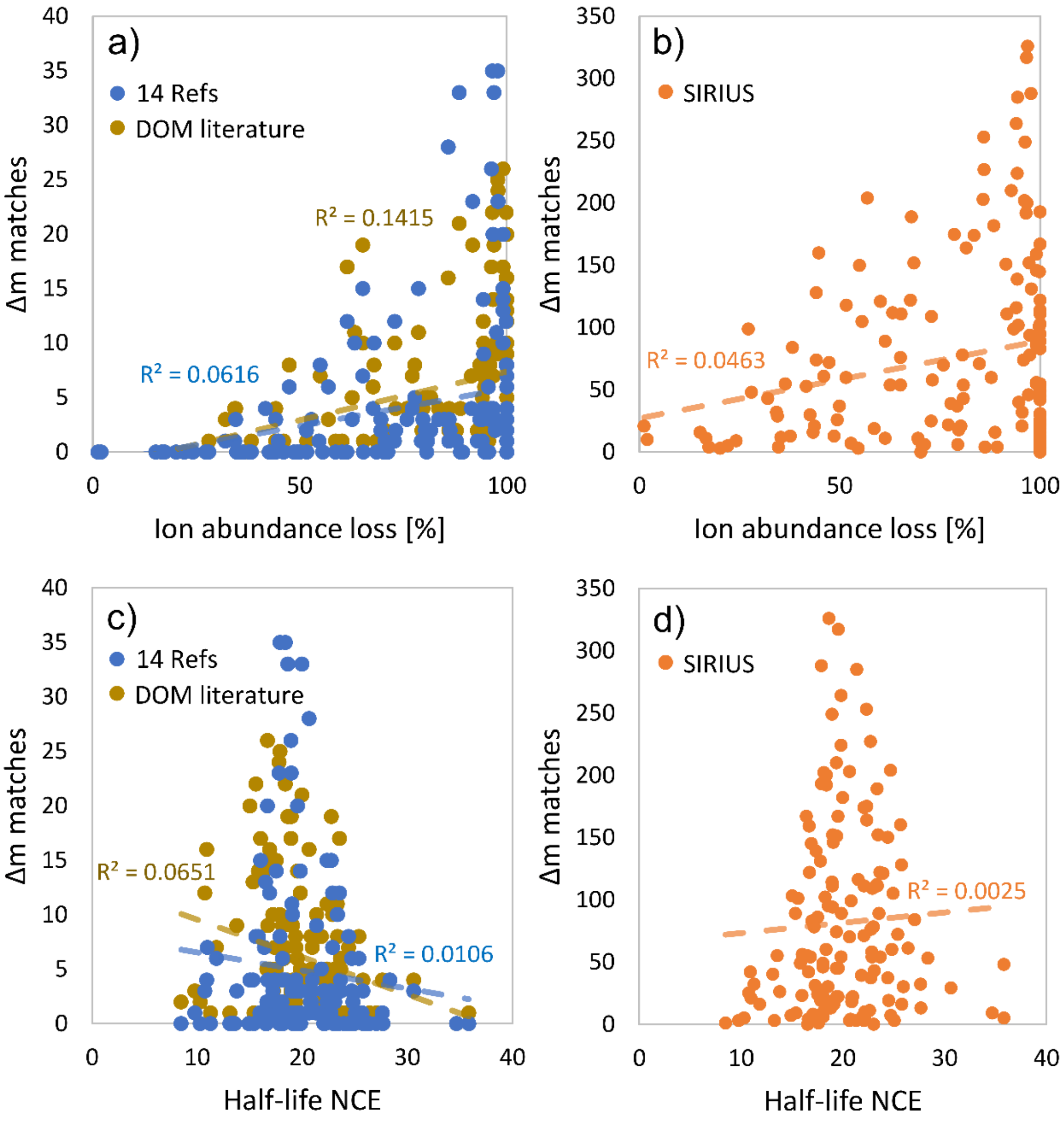

Figure S-8. The number of matches in relation to soil porewater DOM precursor ion fragmentation sensitivity, expressed as ion abundance loss (upper panels a and b, % change in ion abundance between NCE 0 (non-fragmented) and NCE 25) and half-life NCE, i.e., the NCE level at which initial ion abundance has decreased by 50% (obtained by linear fits; lower panels c and d). Panels a and c show matches against literature-known DOM Δm's (brown, **Table S-6**) and against Δm's observed in reference compound data ("14 Refs", blue, **Table S-7**) and panels b and d show matching against a larger list of SIRIUS Δm features (available in the openly available datasets, see introduction of this document). Fragmentation sensitivity is a poor predictor of match number, but obviously, a precursor ion needs to fragment to some degree in order to indicate positive matches. Best fit-curves are linear regressions.

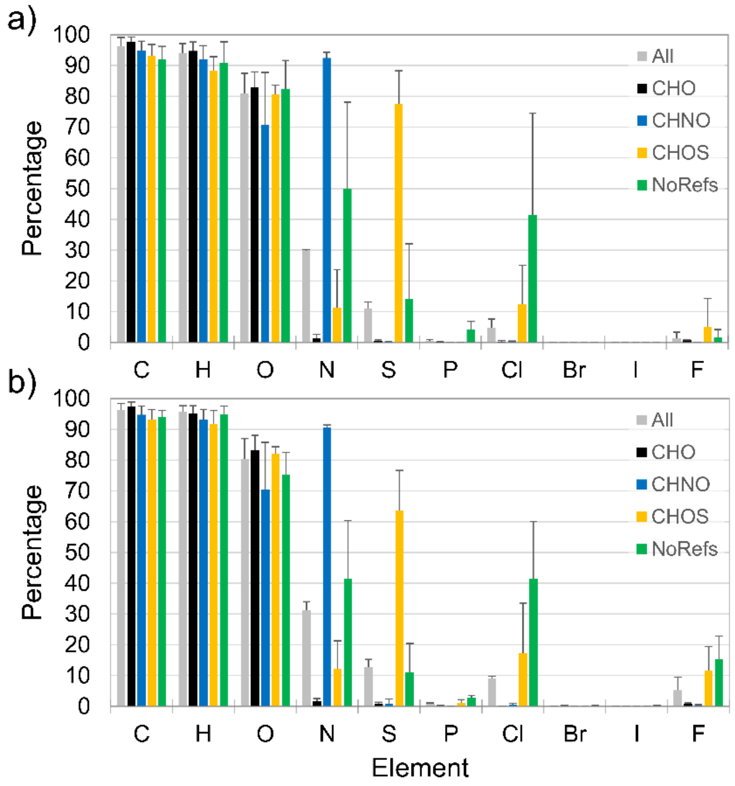

Figure S-9. Matching against the SIRIUS list of Δm's for **a)** soil porewater DOM and **b)** SRNOM. Results show the average +/- standard deviation of the precursor ions (159/ 221 peaks in total, 127/ 144 with an assigned molecular formula, respectively). The figure shows the relative number of Δm's containing ten elements, divided into five sets of features (colored bars): All precursor ions („All", grey), precursor ions only assigned with a formula containing elements C, H and O („CHO", black), precursor ions with an assigned formula also containing nitrogen („CHNO", blue) or a sulfur atom („CHOS", orange), and precursor ions with no molecular formula ("NoRefs", green). As expected, elements C, H, and O were part of nearly all matched Δm's, reaching > 80% coverage. N-and S-containing Δm's, although only present in ~ 30% and ~10% of all matches (grey bars), showed highly consistent matching to CHNO (blue bars, >90% coverage) and CHOS formulas (yellow bars, ~ 60-80% coverage); likewise, CHO-only formulas indicated no matching to N- and S-containing Δm's (black bars in "N" and "S" columns). Elements Cl, F and P and were the main additional elements found to match (but not Br, I). As expected from the literature, less than two percent of peaks indicated matching to P-containing Δm's, but Cl- (5-10% of all precursor ions) and F-containing (2-5% of all precursor ions) peaks were predicted especially for non-assigned peaks (green bars in "Cl" and "F" columns). The detection of these Δm's offers a way to evaluate the reliability of the formula assignment procedure (which did not include elements Cl, F and P). The matching of P- and Cl-containing Δm's can be explained in two ways: 1) by the presence of precursor ions without an assigned formula (green bars). For example, the three non-assigned features *m/z* 301.0485/ 301.0120/ 240.9910 matched to 18/ 22/ 7 P-containing and 38/ 22/ 11 Cl-containing Δm's. Mass 241.0249 matched to two Δm's containing both Cl and F (fluorine; CH2ClF3 = 105.979712 Da, and C2H2ClF3O = 133.974627 Da), which may indicate the presence of a Cl- and F-containing precursor ion. All in all, the matching revealed that most non-annotated peaks were combinations of N- and Cl- and to a lower degree also S- and F-containing formulae. 2) Unresolved elemental compositions (e.g., Cl- and S-containing formulas, i.e., yellow bars in "Cl" and "F" columns) can also contribute to ambiguity: For example, many CHOS-assigned precursor ions at IPIMs 417 and 361 matched to the Δm of C2HClN2 (87.982826 Da). A closer look at the potential molecular formulas at their exact *m/z* with MIDAS Formula Calculator (v.1.2.6, National High Magnetic Field Laboratory, Tallahassee, United States) revealed the presence of a series of N- and Cl-containing formulas within ± 0.5 ppm distance. The series overlapped with the CHOS formulas by a common exchange (C4O7 vs. H5N4S2Cl, 0.08 ppm distance, nominal mass 160), which is hard to resolve even by FTICR-MS instruments. All in all, we found that CHOS

assignments were most affected by this (up to 15% of matches), indicating potential unresolved formulas containing mainly the elements N, Cl and F.

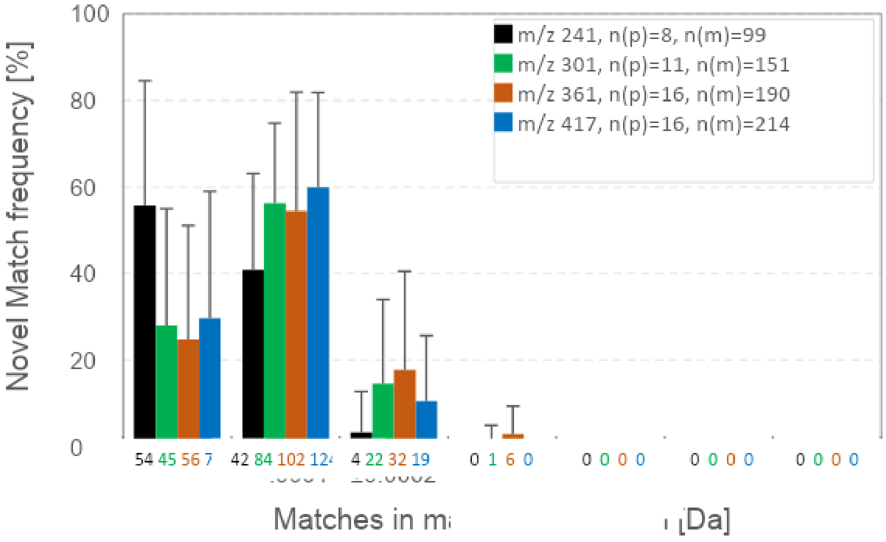

Figure S-10. Changes in Δm matching frequency upon widening of tolerance window. Match frequency of non-indicative Δm's (**Table S-6**) spanning a mass range from 2 – 193 *m/z*. Data for each of the four IPIMs is presented (colors, see legend) along with the total number of precursor ions "n(p)" and the total number of matches across these precursor ions "n(m)". Small numbers below bars indicate absolute numbers of matches (average across all precursors of the respective IPIM). Error bars are +1SD across all precursor ions. Match frequency was then plotted vs. mass tolerance bin (x-axis), indicating how many percent of matches were found in each bin, starting from the exact Δm (exact mass to four digits). The tolerance bin was increasingly widened, and the number of additional ("novel") matches – i.e., those not detected at narrower bin size – was monitored. The plot shows that the majority of matches to non-indicative Δm's were found within the applied tolerance window (± 0.0002 Da). It also shows that outside of this window, the matching frequency drops close to zero, indicating a low match rate in terms of detecting false positives, even when widening the tolerance bin to ±0.001 Da. Note, the analysis of each precursor ion also included a number of Δm's showing no matches within the ± 0.0002 Da tolerance window (often the majority; however, we only used precursor ions here that showed at least seven Δm matches, which translates into a maximum of 47 negative "hits", number of Δm's in the non-indicative list = 54). Also for those Δm's not matched within the applied tolerance window of ± 0.0002 Da, we found no novel (additional) matches in the widened tolerance bins (data included in the figure), indicating that the Δm approach is selective to losses that make chemical sense: We would expect random matches if the calculated Δm's were derived from noise and not from an inherently structured biogeochemical signal. It also indicates that the peaks of interest are adequately resolved.

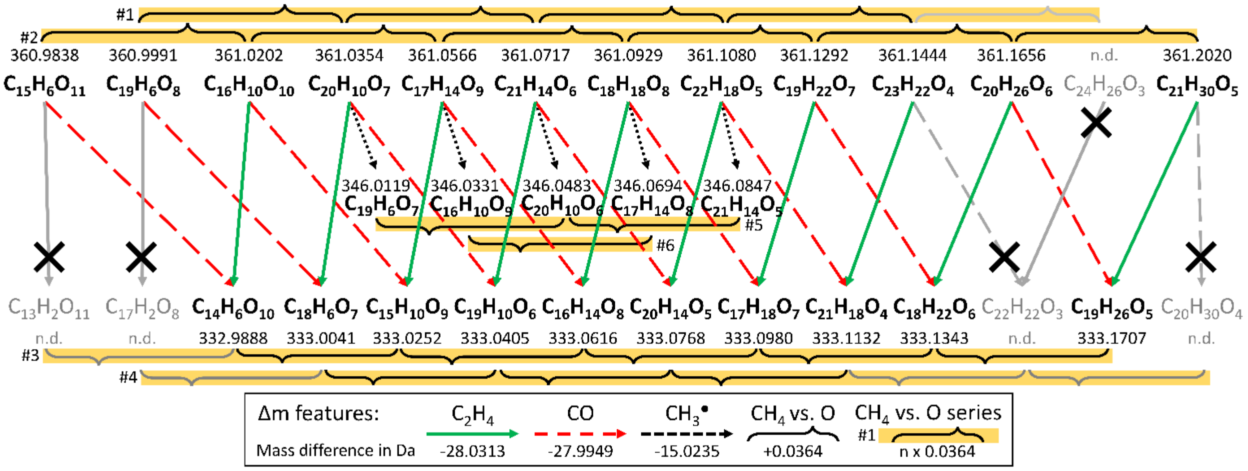

Figure S-11. Link between matches to Δm features $CH_3^•$, CO and C_2H_4 and the occurrence of CH_4 vs. O exchange series on the precursor ion (upper row of molecular formulas, here shown for precursor ions at m/z 361 in SRNOM) and product ion level (mid and lower row of molecular formulas). Two CH_4 vs. O product ion series (#1 and #2, yellow bands) are linked by concurrent losses of CO (red dashed arrows) and C_2H_4 (green arrows) to two product ion series at m/z 333 (#3 and #4) and by parallel losses of $CH_3^•$ (black dotted arrows) to two smaller product ion series at m/z 346 (#5 and #6). Undetected members of the CH_4 vs. O exchange series are shown additionally in grey (black crosses indicate missing Δm match due to undetected precursor or product ion).

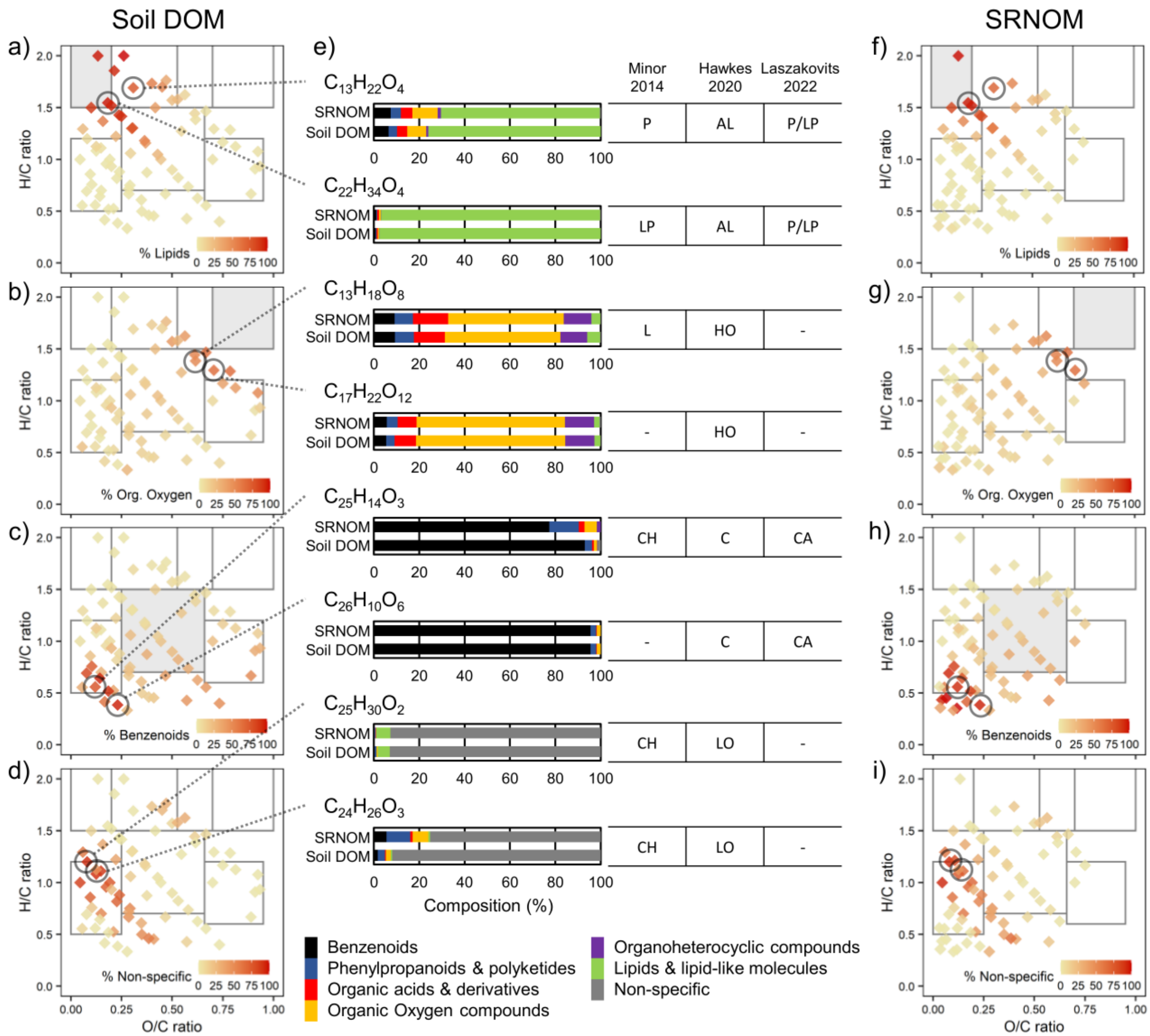

Figure S-12. Compositional variation of individual precursor ions in Van Krevelen space. Composition values were calculated from clustered Δm matching profiles and their structural association as annotated by SIRIUS (see details in the last section of **Note S-1**). Panels a – d and f – i show fractions of lipids and lipid-like molecules, organic oxygen compounds, benzenoids and non-specific information, for soil porewater DOM and SRNOM, respectively. Boxes indicate structural domains as defined in Minor et al. (2014) (**Figure S-4**), and those boxes related to the major structural class are filled grey.[27] Two formulas (precursor ions) are highlighted per plot and shared between soil DOM and SRNOM. For these eight exemplary formulas, structural composition is shown by bar plots in panel e. Structural classes, see legend in panel e. Next to the bar charts, usual a-priori classifications by molecular formula are presented.[27,28,32] Abbreviations: (Minor 2014) P, Protein-like; LP, Lipids; L, Lignin or carboxyl-rich alicyclic molecules (CRAM); CH, Condensed hydrocarbons; (Hawkes 2020) AL, Aliphatic; HO, High O unsaturated; C, Condensed aromatics; LO, Low O unsaturated; (Laszakovits 2022) P, Peptide; LP, Lipid; CA, Condensed aromatic.

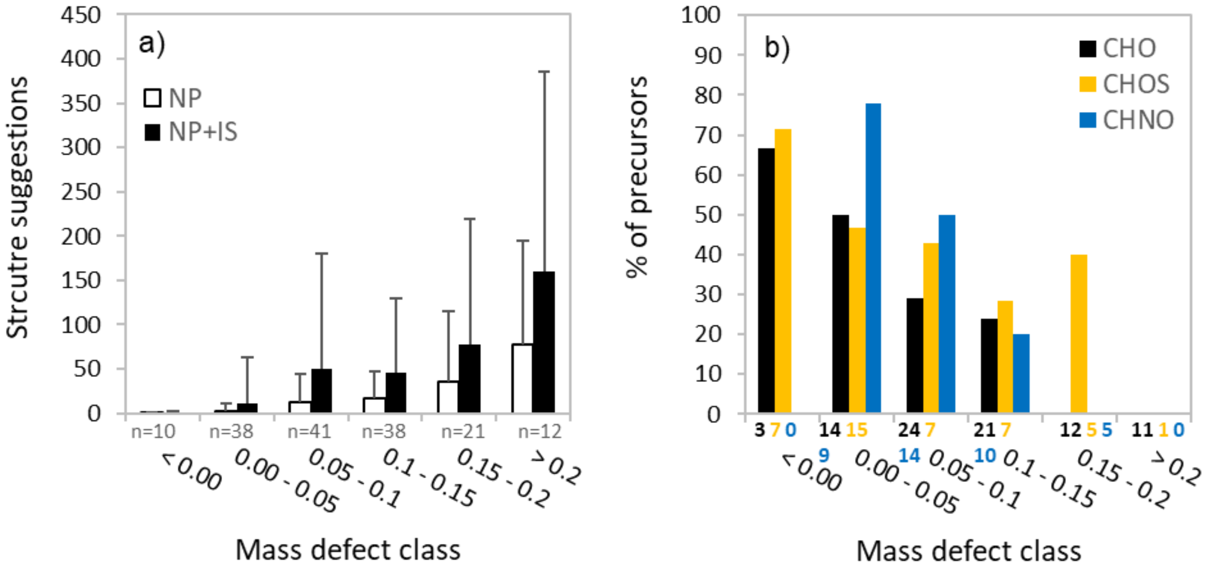

Figure S-13. Effect of mass defect on the number of structure suggestions across both samples. **a)** Average number of structure suggestions from natural product databases ("NP", including including DNP[33], KNApSAcK[34], Metacyc[35], KEGG[36], and HMDB[37]) and in-silico databases using predicted enzymatic transformation products of NP structures from the MINEs database ("NP+IS").[38] The numbers of molecular formulas in each mass defect class are given below bars; error bars represent one standard deviation (negative standard deviation not shown). In-silico querying helped to increase numbers in potential structure suggestions. Formulas with low mass defects showed little hits in all databases considered, in agreement with earlier reports.[39] **b)** Percentage of precursor ions in CHO, CHOS and CHNO formula classes without structure suggestions, depending on their mass defect class. Only the NP+IS set (see panel a) is shown. Absolute numbers of members of each formula class (i.e., representing 100% of that bar) are given below each bar in the corresponding color. The higher the mass defect, the lower the proportion of molecular formulas not covered by structural suggestions. However, especially S- and N-containing precursor ions stand out with an absolute total of 45% (CHOS, n=19) and 42% (CHNO, n=16) of precursor ions with no structural suggestion even after in-silico extension of NP database suggestions by known enzymatic transformations, compared to only 25% (n=21) of CHO precursor ions.

Note S-1. Supplementary experimental details

Reference compounds and reagents. We chose a set of 14 aromatic reference compounds as representative plant metabolites in DOM (Figure S-1). All compounds (**Figure S-1**) were first dissolved in one ml of ultrapure MeOH (BioSolve BV, Valkenswaard, the Netherlands; amounts given in mg in **Table S-1**) and kept at -18 °C upon further use. One ml ultrapure water (MQ, 18.2 MΩ*cm @ 25°C, Merck Millipore, Burlington, MS, USA) was added to each stock and thoroughly mixed. In the case of Ellagic acid (**#8**), 100µl DMSO (Dimethylsulfoxide) were added to the stock to aid in dissolution and vortexed for 15 min at 45°C. Afterward, the stock solution was centrifuged for 1 minute at 17500 rcf (Hermle Z233 MK-2, Hermle Labortechnik GmbH, Wehingen, Germany). Stocks were diluted (50% MeOH in ultrapure water) to a final concentration of 20 - 200 mg-C/L and kept at 4°C before analysis. The reference compounds can be grouped according to their structural properties: Groups A and B contain only one aromatic ring and differ in the presence of functional groups (A: mainly carboxyl, B: mainly methoxy). Group C contains larger structures containing at least two ring structures from fused subunits (#**7**, quinic acid, and caffeic acid; #**8**, two gallic acid monomers; #**9**, coumaric acid, two gallic acid units, and glucose). Group D contains two flavan-3-ol structures, and group E contains three flavonoids with structurally similar but slightly differing flavon-3-ol structures that were also linked to sugars (glycosides).

Orbitrap tandem MS analysis of reference compounds. We infused the reference compound solutions directly into the ESI (electrospray) source of an Orbitrap Elite (Thermo Fisher Scientific, Bremen).[40] The ESI was operated in negative mode, and solutions were infused at a flow rate of 10 µl/ min. We optimized the Orbitrap response for each substance by tuning sheath and aux gas flows (N_2), spray voltage, S-Lens RF, and the ESI needle position to obtain high-quality Δm features. The scan range was chosen depending on the precursor ion *m/z*. The remaining instrument settings were left unchanged for all compounds (**Table S-2**). We performed collision-induced dissociation (CID) experiments at three normalized collision energy levels (NCE 15, 20, and 25%). MS^3 spectra of selected key product ions were acquired in some cases (aglycons of flavonoids #12 and #13). After recalibration with known product ions (**Table S-3**), all major product ion peaks were annotated with a molecular formula. We annotated molecular formulas by a Matlab routine recently incorporated into an openly available FTMS data processing pipeline.[41] We removed peaks that occurred only once across the normalized collision energy (NCE) gradient or showed a maximum absolute intensity below 1E3 across all tandem mass spectra. Higher-energy collision induced dissociation (HCD) MS^2 spectra were included to confirm low-*m/z* CID product ions. Ion abundance was normalized to the intensity of the base peak of each mass spectrum (fragment spectra described in **Table S-4**). Fragmentation spectra were evaluated with SIRIUS 4.0[13] and CSI:FingerID[14] for quality control and interpretation (**Table S-5**). We calculated Δm's between the precursor ion (always [M-H]- ions, except compound **#6**, M-•) and all product ions. Separate lists were created for each NCE level. To exclude unique but less important Δm's from our analysis, we derived a list of those features (n=55) that were 1) either related to a fragment with a minimum relative intensity (base peak) of 1% or 2) detected more than once across the 14 reference compounds (**Table S-7**). Eight of these Δm's also belonged to the list of literature-known features found ubiquitously in DOM, e.g., the losses of CO_2 and H_2O.[18,20,22,42] These were kept as part of the reference-compound derived Δm feature list for completeness. The comparison of measured to predicted Δm features by molecular formula allowed us to assess assignment errors in our dataset. Above Δm values of 75 m/z, the error between them was below one ppm (**Figure S-2**). As expected, the error peaked at ~5 ppm at a very small Δm range of 15 – 30 m/z.

Processing of tandem MS data: Reference compounds. Raw data were acquired in LTQ Tune Plus 2.7 and processed and exported as an average mass spectrum from Xcalibur (both Thermo Fisher Scientific, Waltham, MA, USA). They were then transformed into mzML format with MSconvert from the Proteo Wizard software package[43] and further processed by the software tool mmass.[44] Peaks were picked at a minimum absolute intensity of 100 and 80% peak height in mmass. We recalibrated the mass spectra by the known exact mass of the precursor ion and plausible product ions at lower *m/z* to improve mass accuracy of unknown product ion peaks and the derived Δm values. Calibrant ion identity was checked for plausibility by a threefold confirmatory approach: 1) Suggested molecular formula in MIDAS (Formula Calculator v.1.2.6, National High Magnetic Field Laboratory (NHMFL), Tallahassee, USA) based on exact mass and wide elemental constraints; 2) Predicted fragmentation products in Mass Frontier 7.0 (Thermo Fisher Scientific); and 3) Reports of fragment identity (molecular formula and structure) from the literature (see references and calibrant ions specified in **Table S-3**). Alignment of fragment mass spectra and molecular formula annotation was achieved via a Matlab routine that is now openly available.[41] The settings for formula annotation were as follows: Minimum allowed H/C ratio, 0.3; maximum allowed O/C ratio, 1; minimum allowed double bond equivalent (DBE), -0.5; charge, -1; min #C, 1; min #H, 1, min #O, 1. The error of most annotated

formulas was within ± 0.5 ppm; the maximum tolerance allowed was ±1 ppm. The upper elemental boundaries for fragment annotation were determined by the reference compounds' neutral molecular formula. Assignments were rechecked with MIDAS, especially the presence of radical anions.

DOM samples. We chose a forest topsoil pore water isolate[45] (**Figure S-5**) and Suwannee River Natural Organic Matter[46] from the International Humic Substances Society (IHSS) as exemplary DOM samples for our analysis. The porewater sample was initially taken in early November 2005 from a sintered glass suction plate system installed in 5 cm soil depth at a long-term monitoring site in a ~50-year old spruce (Picea abies) forest site at Wetzstein, Germany (50° 27' 13" N, 11° 27' 27" E)[47,48], and immediately freeze-dried for storage. The DOM sample was reconstituted in acidified ultrapure water (pH 2, hydrochloric acid, p.a.) to a final concentration of ~ 3 mg-C /L and solid phase-extracted (PPL cartridges, modified styrene-divinylbenzene polymer, BondElut, Agilent, CA, USA) according to a published protocol[49] at a PPL/ DOC ratio of ~ 1400. SPE-DOM was eluted in MS grade methanol and stored at -20°C until further analysis. The extraction efficiency was 86.9 ± 1.4% on a carbon basis (arithmetic mean ± standard deviation, n=3). SRNOM was obtained as a powder from IHSS and reconstituted in ultrapure water to obtain a stock solution of 35.8 ± 3.3 mg-C/L (arithmetic mean ± standard deviation, n=3) that was then extracted like the soil porewater isolate. Extraction efficiency of the SRNOM sample was 79.3 ± 5.3% on a carbon basis (arithmetic mean ± standard deviation, n=2).

Orbitrap tandem MS analysis of DOM. DOM precursor ions group naturally into precursor ion mixtures (herein called isolated precursor ion mixtures, "IPIM", plural "IPIMs") within -0.05 and +0.35 Da of an integer *m/z*.[50] We chose four IPIMs that span the range of maximum ion abundance typically observed in terrestrial DOM samples for fragmentation (*m/z* 241, 301, 361, and 417).[40] Each IPIM of the soil porewater isolate contained a potential tannic forest marker described earlier[45], as based on the H/C and O/C atomic ratios of the respective molecular formulas (monoisotopic masses of $[M\text{-}H]^-$ ions are given in brackets): $C_9H_6O_8$ (*m/z* 240.9990), $C_{11}H_{10}O_{10}$ (*m/z* 301.0201), $C_{13}H_{14}O_{12}$ (*m/z* 361.0413) and $C_{15}H_{14}O_{14}$ (*m/z* 417.0311). The isolation window of the front-end linear ion trap in the Orbitrap Elite was set to 1 Da to isolate a single IPIM. We collected 150 scans per fragmentation experiment (50 in SRNOM data) and ran every experiment twice. We only considered precursor and product ions detected in both replicate fragmentation experiments to exclude potential false-positive signals.[50] We did not observe product ions in the mass defect region between +0.35 and +0.95 *m/z*. The ultrahigh resolution and mass accuracy allowed us to link individual molecular formulas of precursor and product ions, i.e., to deconvolute the data and obtain each individual precursor ions' Δm matching "profile".

Processing of tandem MS data: Unknown DOM precursor ions. The DOM samples were injected at a concentration of 100 mg-C/L into the above described Orbitrap Elite system. The DOM sample was injected at a five-fold higher carbon concentration than in preliminary studies[40,45] to compensate for the low concentration of individual compounds and increase sensitivity in tandem MS experiments.[51] The instrumental settings to create MS^1 data for precursor ion isolation were similar to the method described before and yielded a similar response. All tannic marker signals from the previous study[45] were also found by the Orbitrap in soil porewater DOM, in line with results reported elsewhere.[40] The parameters for the MS^2 experiments were the same as for the reference compounds if not noted differently (**Table S-2**). The scan range was adapted to the precursor ion mass. All other parameters were left as chosen in the initial method.[40] The raw data processing followed the same steps as described for reference compounds. Recalibration lists were constructed from known molecular formulas of precursor ions and ubiquitous non-indicative neutral losses (i.e., multiples of CO_2, H_2O, and CO losses, **Table S-6**)[18,20,22,42] and applied to improve the mass accuracy of the derived Δm data.[52] The final exported peak lists were picked at an absolute signal intensity threshold of 10, equivalent to an S/N > 3. Alignment of fragment mass spectra and molecular formula annotation followed the same routines and with similar settings as described for reference compounds except that the elemental boundaries for fragment annotation were: C, 1-40; H, 1-200; N, 0-4; O, 1-40; S, 0-2. For data cleanup, we first removed peaks that were only detected once across all tandem mass spectra as they are prone to be noise. Molecular formulas with unlikely combinations of heteroatoms ($N_{2\text{-}4}S$ and $N_{2\text{-}4}S_2$) were classified as unassigned peaks, and if multiple formulas were proposed, preference was given to the CHO formula.

Assessment of precursor ion properties. The fragmentation sensitivity (change in precursor ion intensity upon fragmentation) and the number of matches to common mass differences (**Table S-6**) were checked on the single precursor ion level in soil porewater DOM to assess differences between molecular formulas (*m/z* 241, **Table S-9**; *m/z* 301, **Table S-10**; *m/z* 361, **Table S-11**; *m/z* 417, **Table S-12**). We determined the fragmentation sensitivity in two ways, as the relative (%) change in ion abundance at different NCE levels based on the initial values (non-fragmented)

and as a "half-life NCE", denoting a 50% decrease in initial ion abundance (derived from linear regression of ion abundance data). This allowed us to relate properties such as the number of CO_2 losses to the initial ion abundance or fragmentation sensitivity of the precursor ion and its molecular formula. We calculated commonly used molecular indices from the molecular formula data, such as ion-abundance weighted averages of the number of atoms per formula (C, H, O), the number of double bond equivalents (DBE), the aromaticity (AI_{MOD}), or the nominal oxidation state (NOSC) of the IPIMs.[24,26,53]

Collecting Δm from reference data. We collected 249916 negative ESI reference spectra of 17994 unique molecular structures from the GNPS[31] (https://gnps.ucsd.edu/), MassBank[30] (https://massbank.eu/), MoNA (https://mona.fiehnlab.ucdavis.edu), and NIST (https://www.nist.gov/srd/) spectral libraries. Spectra were measured on Orbitrap or Q-ToF instruments. While the molecular formula of the precursor ions was known, we putatively annotated all product ions with SIRIUS (version 4.9).[13] All molecular formula differences between the precursor ion and the annotated product ions were collected. We report 11477 molecular differences and Δm's that occur in at least three different compounds. For some compounds there were multiple measurements; for normalization, we divided the number of occurrences of each Δm in each compound by the number of measurements for this compound. In a next step, we annotated all reference compounds with compound classes using the ClassyFire webservice.[29] For each pair of compound class and Δm, we performed a Fisher's exact test[54] to check if the Δm is specific for the compound class. The p-values are multiplied with the number of compound classes (Bonferroni correction). For each Δm we then reported the top 15 compound classes with p-value above 0.001. We excluded compound classes which are non-informative, namely, "Organic nitrogen compounds", "Organonitrogen compounds", "Organosulfur compounds", "Organic oxygen compounds", "Organooxygen compounds", "Organic acids and derivatives","Lipids and lipid-like molecules", "Chemical entities", and "Organic compounds".

Δm matching and data analysis. We obtained the Δm's of every combination of precursor ions and product ions, yielding a Δm matrix for each of the four IPIMs at three NCE levels (15, 20, 25) for the soil porewater isolate and one NCE level (25) for SRNOM. To match sets of known Δm's and DOM Δm matrices, exact Δm's were cut behind the fourth digit. We matched DOM against three lists of known Δm features: a) features ubiquitously found in DOM as reported in the literature (**Table S-6**), b) features from a set of 14 selected aromatic reference compounds (**Table S-7**, **Figure S-1**) that could represent structural features of plant-derived DOM molecules, and c) 11477 Δm features from the 249916 reference compound spectra annotated by SIRIUS as described in the previous section. The tolerance for a positive match with the DOM Δm matrix was set to ± 0.0002 Da (2 ppm at 200 Da), thereby roughly accounting for the mass error of two *m/z* measurements (precursor and product ion). We assessed the probability of a false positive match and accounted for molecular formula constraints to evaluate our approach's validity. To analyze patterns of matching frequency, we visualized precursor ion formulas in Van Krevelen space.[55] We compared individual matching profiles of reference compounds and DOM precursor ions to evaluate the potential identity of underlying unknown structures by two-way hierarchical clustering using Ward's method and Euclidean distance in PAST (v3.10).[56] Clustering was also visualized by ordination (PCA) in the same software environment. Precursor ions that only matched to literature-known (ubiquitous) Δm's were disregarded from the multivariate analysis, but were considered in separate analyses focusing on N- and S-containing precursor ions and those containing only carbon, hydrogen and oxygen (CHO). The matching data was then combined for each NCE level and transformed into presence/ absence format. To evaluate the predicted potential structures of DOM precursor ions based on their matching with compound class-associated Δm features, we assessed structure suggestions as an independent source of structural information. We assessed structure suggestions from different natural product databases, including Dictionary of Natural Products[33], KNApSAcK[34], Metacyc[35], KEGG[36], and HMDB.[37] Additionally, we also included in-silico suggestions based on known natural product structures and their potential enzymatic transformation products based on the MINEs database.[38] The InChi-Key of structures was used to exclude stereoisomers and classify structures into major scaffold types by ClassyFire.[29]

Calculation of structural composition based on SIRIUS-annotated Δm features. The MS^2 data allow to determine approximate percentages of substance groups per molecular formula. This is possible for two reasons: 1) structural information can be derived from the Δm matching of individual DOM precursors (called deconvolution herein); and 2) association of Δm features with dedicated molecular structures (SIRIUS annotation via Classyfire ontology). We excluded all N- or S-containing precursor ions (only CHO) and Δm features (mass differences) that were matched less than 10 times across all precursor MS2's (number of Δm's remaining = 725). The analysis was based on list c (see experimental section in the main text) consisting of 11477 Δm features from a negative ESI MS2 library with 249916 reference spectra of 17994 unique molecular structures that were annotated by SIRIUS (**Figure S-3**). As described for

list b in the prior section, the Δm matching matrix was then clustered by two-way hierarchical clustering using Ward's method and Euclidean distance in PAST (v3.10). The cluster containing the common neutral losses of CO, CO_2 and H_2O was chosen as the reference cluster (all Δm features contained in that cluster were the following: O2, C2O4, C2O3, CO, C3H4O2, C2H4, CH2O3, CH4O, C3H4O3, H2O, CO2, C4H8O2, C2H6O2, C2H8, C3H8O2, CH6, C2H4O, C3H8 and C4H8O). In total, 26 Δm clusters were differentiated that way. Using the SIRIUS-annotated substance groups specific to the mass differences, we then derived the association of the mass difference clusters with structures. For that, we counted the Classyfire classes associated with each Δm feature and summed them up per Δm cluster. Classifications at levels >3 of the Classyfire ontology were aggregated based on level 3 classification, yielding a count sum per Δm cluster for the classes “Benzenoids”, “Phenylpropanoids and polyketides”, “Organic acids and derivatives”, “Organic oxygen compounds”, “Organoheterocyclic compounds” and “Lipids and lipid-like molecules”. Associated classes of structures that were wrongly assigned because we only analyzed CHO formulas here (e.g., amino acid classes), encompassed the following classes: Phosphocholines, Quarternary ammonium compounds, Acetamides, Alkylindoles, Tosyl compounds, S-containing compounds, Serine and derivatives and Aspartic acid derivatives. These were in total 24 structure associations compared to a total of 578. Based on the total count of structure specificities per cluster, we calculated the association of the cluster per structural group as (Formula 1):

$$F_{Class} = \frac{A_{Class}}{A_{Cluster}} \qquad (1)$$

where F_{Class} denotes the fraction of structural association of a cluster, $A_{Cluster}$ denotes the sum of all associations per cluster, and A_{Class} denotes the sum of all associations of a specific structural class per cluster. F-values were calculated for all combinations of 26 Δm clusters and six structural classes. If a cluster contained no Δm features associated with structural features, we counted is as “non-specific” and defined this as a seventh structural class with F = 1 (and all other classes F = 0). Such Δm features were not considered in clusters with at least one Δm feature with a structure specificity; instead, we regarded them as co-clustered and therefore likely similar. Next, we calculated the coverage of each Δm cluster per precursor matching profile as (Formula 2):

$$C_{Cluster} = \frac{\Delta m_{Matches}}{\Delta m_{Cluster}} \qquad (2)$$

where $C_{Cluster}$ denotes the fraction of matched Δm features of a Δm cluster, $\Delta m_{Matches}$ denotes the number of matched Δm features of a Δm cluster, and $\Delta m_{Cluster}$ denotes the sum of all Δm features of a Δm cluster. The dominance of a cluster was then calculated as

$$D_{Cluster} = \frac{(n_{Matches,Cluster} * C_{Cluster})}{n_{Matches,Precursor}} \qquad (3)$$

where $D_{Cluster}$ denotes the dominance of a Δm cluster compared to all other Δm clusters per precursor ion, $n_{Matches,Cluster}$ denotes the number of a Δm cluster per precursor ion, and $n_{Matches,Precursor}$ denotes the number of all Δm matches per precursor ion. Precursor ion $C_{32}H_{18}O$ was excluded because it had no matches. We then multiplied C and D-values for all combinations of 26 Δm clusters and 149 precursor ions to obtain a measure of each Δm cluster’s importance for a precursor ion called I. This metric therefore condenses the Δm matching profile of each precursor to 26 dimensionless numbers. Using the F and I values of each cluster, we calculated their sum product (dot product) across all 26 clusters for each of the seven structural classes (and for every of the 149 precursor ions) (Formula 4):

$$CP_{Class,absolute} = \sum_{i=1}^{26} F_i * I_i \qquad (4)$$

where $CP_{Class,absolute}$ is the sum product of a structural class across all 26 clusters for an individual precursor ion. This translates the compositional information contained in a precursor ion’s Δm matching profile and its structure-associated Δm features into a metric. In a last step, we calculated the relative CP value ($CP_{Class,relative}$) for each structural class per precursor ion by dividing the classes’ sum product by the summed sum product of all seven structural classes and multiplying it by 100 to yield percentage values (Formula 5):

$$CP_{Class,relative}\,[\%] = \frac{CP_{Class,absolute}}{\sum_{i=1}^{7} CP_{Class,absolute,i}} * 100 \qquad (5)$$

This compositional information was used to visualize trends for each structural class in Van Krevelen space, showing individual precursor ions based on H/C and O/C ratios of their assigned molecular formula (**Figure 3**, **Figure S-12**).

Note S-2. Detailed description of reference compound fragmentation behavior

General note on CO_2 loss and CH_4 vs. O exchange. We observed CO_2 losses in nine reference compounds but this was not limited to the presence of carboxyl functionalities (as in substances **#1-3**).[57] Ring cleavage and rearrangement reactions from neighboring hydroxyl or carbonyl/ keto functionalities also produced a neutral loss of CO_2 and did so at similarly low collision energies as observed for carboxyl functions. For example, we observed CO_2 losses in flavonoid aglycons (spiraeoside **12***, and quercetin **13***, but not in myricetin **14***, MS^3 data not shown) or catechin (**10**), and to a lower degree also in ellagic acid (**8**, originating from lactone functionalities).[3,6,9,11,20,58] Regarding the CH_4 vs. O exchange that is commonly observed in DOM[59], it is notable to report the observation that both methoxy-phenols indicated a formal O vs. CH_4 insertion. Ion abundance of the oxidized product was below 1% at NCE 0 and increased to 2% (#5, m-Guaiacol) and 17% (#4, Creosol) at NCE 15. Δm values were only calculated for the non-oxidized product ion. A significant link between losses of CO and C_2H_4 units also explained the appearance of regular spacings of CH_4 vs. O series in product ions (see section 3.4 in the main text, and **Figure S-11**).

Group A: Small carboxy-phenols (#1, #2, #3, black numbers in Figure S-1). A dominant CO_2 loss characterized the three small carboxy-phenols (**#1** Vanillic acid, **#2** Hydroxy-cinnamic acid, **#3** Gallic acid). Vanillic acid (**1**) showed two major loss patterns. The precursor ion initially lost either the methyl radical from its 3-methoxy group (loss of 15.0235 Da) or its carboxyl group (-CO_2, -43.9989 Da), leading to product ions *m/z* 152 or 123. The subsequent loss of the methyl radical from product ion 123 produced a minor signal at *m/z* 108. The 4-hydroxy-function was not affected by fragmentation. Another minor fragment at *m/z* 81 indicated a ring rearrangement reaction after the loss of a C_2H_2O group from *m/z* 123. Hydroxycinammic acid (**2**) and gallic acid (**3**) behaved similarly to (**1**) in that they lost the attached carboxyl group and that attached 4-hydroxy (**2**) or 3,4 and 5-hydroxy groups (**3**) were not affected by fragmentation. The absence of a methoxy group (OCH_3) in these structures seemed to limit possible fragmentation reactions to the CO_2 loss as compared to substances **4**, **5,** and **6**.

Group B: Small methoxy-phenols and methoxy-quinones (#4, #5, and #6, greyish numbers in Figure S-1). Vanillic acid (**#1**) shared with members of group B the presence of a methoxy group, which gave rise to the loss of a methyl radical ($CH_3^{\bullet}$). The methoxy-phenols Creosol (#**4**) and m-Guaiacol (#**5**) both showed a major loss of a CH_3 radical and a minor one with a mass difference of 28.0313 Da, being indicative of a C_2H_4 loss. As m-Guaiacol only contains one methoxy group, the mechanism leading to their common C_2H_4 loss is probably related to a ring-opening reaction involving the loss of a dien ($H_2C{=}CH_2$). Similar to **1**, also a C_2H_2O loss was observed directly from the precursor ion of **4** (but not **5**), leading to a minor fragment at *m/z* 95; this could indicate that the proximity between attached hydroxyl and methoxy groups governs the formation of this fragment as they were in neighboring positions in structures **1** and **4** but not in **5**. Structure #**6** (2,3-Dimethoxy-5-methyl-1,4-benzoquinone) showed two subsequent losses of methyl radicals from its neighboring 2- and 3-methoxy groups but no loss of a CO unit as expected from the literature.[60] As the precursor itself formed a radical anion, the first product ion at m/z 167 was a regular ion. A subsequent abstraction of a methyl radical then led to the formation of a new radical anion at *m/z* 152. MS^3 experiments with product ion 167 also showed the formation of the *m/z* 152 ion but also showed a competing (minor) loss of CO (product ion m/z 139), possibly from the "free" oxygen of the former methoxy group.

Group C: Linked carboxy-phenols (#7, #8, and #9, blue numbers in Figure S-1). Group C was mainly characterized by cleavage of ester bonds (e.g., loss of quinoyl or caffeoyl moieties from #**7**). The intramolecular lactone bonds in ellagic acid (#**8**) were, in contrast, exceptionally stable upon fragmentation and yielded rich product spectra only at higher relative NCE (> 25), featuring indicative CO losses[60], but also losses of CO_2 as noted above. Chlorogenic acid (#**7**) is the ester of caffeic and quinic acid (here, cis-3-O-caffeoylquinic acid). It was relatively unstable upon collision with N_2 and nearly fragmented to completeness at NCE 20, yielding one major product ion at *m/z* 191. Two initial losses occurred, with the balance being shifted to the loss of the caffeoyl moiety (quinic acid ion, $[M\text{-}162\text{-}H]^-$, producing the major product ion at *m/z* 191) and a subsequent H_2O loss (minor product ion at m/z 173). In line with previous observations[2,5], the initial loss of quinic acid from the precursor was not as dominant (caffeic acid ion, $[M\text{-}174\text{-}H]^-$, at *m/z* 179) and showed subsequent minor losses of CO_2 (*m/z* 135) or H_2O (*m/z* 161). Ellagic acid (#**8**), the dilactone of gallic acid (**3**), showed remarkable stability and only yielded minor product ions at NCE 20. Rich product ion spectra were only obtained at higher energies (NCE 30–40), which were not applied to DOM in this study. The structure fragmented in a diverse set of consecutive "CO-loss series", starting with, for example, a direct abstraction of CO from the precursor ion (product ion *m/z* 273), or the loss of a CO_2 group (*m/z* 257), all being somewhat related to the internal lactone structure. In total, seven of those series were predicted by SIRIUS 4.0 through several combinations of CO, H, OH, CO_2, or H_2 losses, all leading to the opening of the four-ring structure. Water

losses were predicted to stabilize fragments and competed with CO losses (Figure S-13). The main neutral losses of two CO units and a CO_2 unit yielded the known major product ions detected (besides *m/z* 257) at *m/z* 229, *m/z* 201, and *m/z* 185.[3,61]The minor ion at *m/z* 145 was predicted to originate from a chain of one initial CO_2 loss and four consecutive CO losses. The major losses from structure #**9** (6-o,p-Coumaryl-Di-galloyl-glucose) were the complete abstractions of the coumaryl subunit (-164 Da, $C_9H_8O_3$) and galloyl unit (-170 Da, $C_7H_6O_5$), leading to both major product ions at *m/z* 465 and 459. Incomplete loss of the coumaryl (-146 Da, $C_9H_6O_2$) or galloyl unit (-152 Da, $C_7H_4O_4$) were also observed (retention of oxygen at the core structure), the former only in HCD mode. This incomplete loss was also observed at product ions *m/z* 459 (losing an incomplete galloyl moiety) and 465 (losing the incomplete coumaryl moiety), yielding the same product ion at *m/z* 313.

Group D: Flavanol-related structures (#10 and #11, orange numbers in Figure S-1). Compounds #**10** and #**11** (group **D**) shared a $C_6H_6O_3$ loss (unmodified A ring in #**10**, abstraction of trihydroxy-benzene from gallate unit in #**11**).[9,62]Catechin (#**10**) had the most diverse product spectrum among all compounds investigated, including some indicative Δm's of retro-cyclization reactions (fragments at *m/z* 205, 203, 179, 151, 125, and 109, **Table S-5**).[7,10,63] The CID mass spectra of Catechin (#**10**) were composed of a high number of product ions already at rather low normalized collision energies of NCE 15. The fragmentation began with an initial loss of H_2O leading to a product ion at *m/z* 271[62] or, the more dominant reaction, with an initial CO_2 loss to yield the product ion *m/z* 245.[7] The exact mechanism of the CO_2 loss is debated[64] but seems to involve the rearrangement of the structure which contains no peripheral carboxyl functionalities. Further main product ions were found at *m/z* 205 and 203, 179, and additional ones at *m/z* 151 and 125. The product ions 205 and 203 have been reported as products of cleavage of the A ring of the Catechin structure.[7] Fragmentation tree prediction by SIRIUS 4.0 indicated an initial C_3O_2 loss as the starting point of this reaction. The product ions at *m/z* 179, 151, and 125 are predicted downstream fragments from *m/z* 205 after further losses of C_2H_2, CO, and C_2H_2 units. The remaining product ion at m/z 125 is likely a phloroglucinol unit ($C_6H_6O_3$). Compound #**11** (Epigallocatechin Gallate, EGCG), containing a flavan-3-ol subunit, resembled especially #**9** through the presence of a gallate subunit that produced similar Δm's: An incomplete galloyl loss with retention of H_2O ($C_7H_4O_4$), a galloyl loss ($C_7H_6O_5$), or a combined galloyl and H_2O loss ($C_7H_8O_6$); these Δm's were thus chosen as markers of a (potential) gallate loss in DOM. Much similar to **10**, also EGCG was characterized by initial losses of H_2O or CO_2. The SIRIUS 4.0 fragmentation tree predicted that the CO_2 loss is the one that leads to further downstream fragments, with a further dominant loss of a C_5H_6O unit leading to the first dominant product ion at m/z 331, being indicative of a $C_6H_6O_3$ loss (benzene-triol originating from ring A, B or the gallic acid substituent, GAL).[9] Due to the proximity of phenolic hydroxyl groups at ring B and the GAL unit, it is likely that the initial CO_2 loss starts there. Another branch of the tree connects the initial CO_2 loss to subsequent $C_6H_4O_2$ and H_2O losses (a cumulative loss of the GAL unit, -170.0215 Da), yielding product ions at *m/z* 305 and 287. This indicates the stepwise abstraction of the linking CO_2 ester from the flavan-3-ol.[9] The lost gallic acid unit also forms a diagnostic fragment at m/z 169, similar to the benzene-triol unit at m/z 125 (the latter only visible in HCD fragmentation mode).

Group E: Flavonol glycosides and aglycones (#12, #13, and #14, red numbers in Figure S-1). The flavonoids (#**12** Spiraeoside**, #13** Isoquercetin**, and #14** Myricitrin) under study indicated the initial abstraction of their attached sugar, as a neutral loss of 162 (**12**, **13**, both glucose) or 146 (**14**, mannose), yielding the remaining aglycon flavonol structure as the main fragment.[12] The sugar moieties did not produce a compatible fragment ion. The sugar loss led to either an anion or a radical anion aglycon. The ratios of both product ions differed among the three substances.[5] Substance **12** did only yield the anion form while substances **13** and **14** also produced the radical anion forms, with **14** producing dominantly the radical anion (**12**, even-electron ion form of aglycon dominated; **13**, equal; **14**, radical anion (odd-electron ion) form dominated). This effect has been attributed to the exact location of the glycosylation site.[5] This effect also influenced the further fragmentation of the aglycon, which proceeded in **14** (less so in **13**) but not in **12**. A further collision of the flavonol aglycon ion (m/z 301 of **12** and **13**) led to the detection of diagnostic fragments at m/z 178.9986 and 151.0037 (and others at *m/z* 121 and 107), originating from a retro-cyclization reaction at ring C upon loss of the B ring.[11] This opens up a way to differentiate the flavonol structure from the flavanol structure (**#10**, present also in substance **#11**), which yielded major product ions at close *m/z* locations (179.035 and 151.0401). The flavonol aglycone structure also showed initial losses of CO_2 and CO from the C ring involving the carbonyl-O (position 4) and hydroxyl-O (position 3) at the C ring.[11,65]

Note S-3. Properties of selected IPIMs and behavior of non-responsive DOM precursor ions

The four chosen IPIMs differed in molecular composition (monotonic, significant, Pearson, $p<0.05$): Heavier IPIMs were less aromatic (AI_{MOD}) but more olefinic (DBE) and oxidized (NOSC) and more diverse in terms of precursor and product ions (n_{max} = 44 and 491, respectively; **Table S-8**). Fragmentation was selective in terms of mass defect across all IPIMs. With increasing NCE, the remaining mixture of precursor ions significantly decreased in mass defect, O/C and NOSC, and increased in average DBE, DBE-O, and AImod (ion abundance-weighted averages; **Table S-8**), which translates to a selective fragmentation of C=O and C-C bonds vs. C=C bonds or ring structures. IPIMs also became more similar in molecular composition upon fragmentation (i.e., average H/C, O/C, etc.; not shown), suggesting common properties among precursor ions resisting fragmentation. This finding supports the view that DOM's structure is based on a limited set of regular backbone structures with similar properties[58,59,66,67] but could also point to similar rearrangements of remaining precursor ion structures upon NCE increase.

Single precursor ions showed zero or slightly positive changes in ion abundance with increasing collision energy in the soil porewater sample. The respective formulas had an average O/C ratio of 0.19 and were of low initial ion abundance (average, 100 a.i.), which at maximum doubled until the highest applied energies. The fraction of ion abundance of these minor signals was equivalent to 0.5% of total initial ion abundance and thus negligible. Such effects are not unexpected, as ion detection might be hampered by space-charge effects in the Orbitrap cell.[68] However, the small change in abundance of single signals documents that those effects were negligible in our analysis and affected only a group of minor signals that were insensitive to fragmentation.

Note S-4. Δm matching: Proof-of-concept data and key findings

In line with continua reported in **section 3.2** of the main text, we found distinct trends in the Van Krevelen distributions of Δm losses in both DOM samples, namely serial losses of CO_2, CO, and CH_2 units (**Figure 2a – c, g – i**, **Table S-13**). Precursor ions with high O/C ratios expelled up to four CO_2 units (soil porewater DOM, $r = 0.52$, $R^2 = 0.27$, $n = 127$, $p < 0.001$; SRNOM, $r = 0.63$, $R^2 = 0.39$, $n = 144$, $p < 0.001$) whereas precursor ions with low O/C ratios showed subsequent losses of up to four CH_2 units ($r = -0.26$, $R^2 = 0.07$, $n = 127$, $p = 0.003$; $r = -0.16$, $R^2 = 0.03$, $n = 144$, $p = 0.056$). Precursor ions with low H/C ratios tended to expel up to two CO units ($r = -0.33$, $R^2 = 0.11$, $n = 127$, $p < 0.001$; $r = -0.23$, $R^2 = 0.05$, $n = 144$, $p = 0.005$).

We used two approaches to check the Δm matching procedure: 1) through the constraint that is imposed by the annotated molecular formula of a precursor ion (which determines the stoichiometry of potential losses), and 2) by widening the tolerance window used to detect a positive match (which should indicate randomness, i.e., an increase in the number of matches if the data was affected by low resolution or low sensitivity). As expected, precursor ions did not lose more atoms as predicted by their molecular formula: Precursor ions rich in oxygen were predicted to expel more oxygen-containing Δm's than oxygen-poor precursor ions that tended to lose CH_2 or $CH_3^{\bullet}$ (and CO) units instead. Most notably, no precursor ions matched to a Δm that would have exceeded the number of atoms present in their assigned molecular formula, a condition that has not always been met in earlier studies.[58] Sulfur- and Nitrogen-containing precursor ions – and only those – dominated the release of S- and N-containing Δm's, respectively (such as SO_3 or CH_2N_2).[15,23,51]A second matching exercise against a library of 11477 Δm's substantiated this finding (**Figure S-9**). We furthermore did not observe an increase in the number of false-positive matches upon widening of the tolerance window applied during the Δm matching process (**Figure S-10**, increase up to +/- 5 ppm at a mass difference of 200 Da). Lastly, precursor ions resisting fragmentation did not match any Δm, whereas "labile" precursor ions fragmented to relative completeness showed a wide range of matches (**Figure S-8**).

Most precursor ions in our study were successfully annotated with a molecular formula containing the major elements C, H, N, O and S, and as indicated above, this was substantiated by matching to respective Δm's of correct mass and elemental composition. However, a minor number of unannotated and sulfur-containing (CHOS) precursor ions did indicate the presence especially of Cl, but also P and F (but not Br or I, which were also part of the SIRIUS Δm list, **Figure S-9**). The presence of Cl and F could also point to common adduct ions (Cl) or contaminants from Teflon filters (F) that may be artifacts of sample preparation or ionization conditions. Despite this uncertainty, which was not the focus of the present study, our results demonstrate the general usefulness of MS^2 information for those studying disinfection byproducts or organic nutrients by FTMS.[69–71]

Note S-5. Potential esterification of DOM by methanol during SPE and storage

We observed indicative losses of methyl radicals that may originate from methoxy functionalities of aromatic ring systems[42,72], such as lignin, which contains methoxylated monolignol building blocks (coniferyl, sinapyl alcohol). We also found 13 matches to the Δm equivalent to a CH_2O loss in the soil porewater isolate and 19 in SRNOM, which is thought to be indicative of methoxy functionalities.[72] However, none of the methoxylated reference compounds showed a CH_2O loss. The presence of methoxyl groups could, in principle, also relate to the potential methyl ester formation between carboxyl functionalities and methanol used for solid-phase extraction (SPE).[73] However, the soil porewater DOM sample used herein was freshly extracted (as opposed to the SRNOM extract which was stored for >2 yrs at -20°C) and thus not stored for a long time (< 2 weeks at -20°C). We showed recently that the ^{14}C signal of the same sample was not diluted by radiocarbon-dead methanol during a dedicated SPE procedure and similar storage conditions.[74] Given that methoxylated structures yielded no CH_2O losses, we argue that the slightly higher number of matches in SRNOM (19 vs. 13) is no sign of longer storage but sample-specific differences in molecular composition. In fact, the higher number in part could be explained by the higher number of precursor ions fragmented (221 vs. 159).

Note S-6. Structural insight into N- and S-containing DOM precursor ions.

Negative-mode ESI CHNO precursor ions generally show few neutral N losses in aquatic DOM and thus have been interpreted as alicyclic or aromatic heterocyclic N such as in imide, pyridinic or pyrrolic moieties that are substituted with carboxyl and hydroxyl groups.[51,75] In line with these earlier reports, we found no evidence of nitrate esters (HNO_3 loss, Δm = 62.9956) in soil DOM. However, most N-containing precursor ions (here, all within ranges $C_{10-23}H_{6-26}N_2O_{1-11}$, n=27 in soil DOM and $C_{9-27}H_{6-26}N_{2,4}O_{1-10}$, n=32) showed a link to N_2 (Δm = 28.0061 Da, 93% in soil DOM, 69% in SRNOM), N_2O (44.0011 Da, 93%/ 63%), and CH_4N_2 (44.0374 Da, 78%/ 59%), and multiple other N losses. Such a diversity of potential N losses contradicts with previous reports, but many N compounds yield fragments in negative ion ESI-MS.[76] Loss of N_2 could indicate direct cleavage under negative ESI conditions, possibly from azo/diazo-functionalities. Lemr et al. (2000) have shown that cleavage of azo/ diazo-N in metal azo-complexes was possible directly (MS^2) or indirectly ($MS^{>2}$) as N_2 or in other reduced forms (e.g., CH_3N, $C_3H_3N_2$, or CHN).[77] Among the specifically correlated SIRIUS Δm features were 14 features assigned to amino acids, peptides or amines in the wider sense that matched to 0-30% of CHNO precursor ions in both samples (among them three proline-related ones, 11-22%) and three linked to dicarboximides with 0-41% of matched CHNO precursor ions (**Table S-20**).

S-containing precursor ions (here, all within ranges $C_{9-24}H_{6-34}O_{2-12}S_1$, n=23 in soil DOM and $C_{9-30}H_{6-34}O_{1-12}S_{1-2}$, n=39) matched with Δm's indicative of sulfonic acids: SO_2 (Δm = 63.9619, 4% of all S precursor ions in soil DOM, 33% in SRNOM), SO_3 (79.95681, 61%/ 44%) and H_2SO_3 (81.97246, 35%/ 31%). Against previous reports, however, we also found potential direct losses of S (31.97207, 65%/ 67%) which could originate from reduced sulfur functionalities, such as thiophenes, thioethers, sulfoxides and thioesters.[23] Other reduced S Δm's were also commonly matched, including CS (43.97207, 78%/ 77%) and CH_2OS (61.98263, 74%/ 56%; possibly as a combination CO+H_2S), which have been observed in positive ionization mode via atmospheric pressure photoionization (APPI) in aromatic reference compounds.[78] This may indicate a more diverse set of S-containing molecules in soil as compared to the deep ocean, where oxidized species seem to dominate.[23] Matched Δm's containing S and > 3 C atoms by tendency contained oxygen atoms as well, which indicates that extensive S-containing aliphatic chains were likely no common structural unit in our DOM sample (dominant reduced Δm features were, as mentioned, S and CS but also C_2H_6S, 62.0190, 52%/ 44%; H_4S, 36.0034, 30%/ 41%, and C_3H_8S, 76.0347, 39%/ 33%); alternatively, they may have been missed due to low ionization or because they resisted fragmentation.[78] Among the specifically correlated SIRIUS Δm features we found three major groups: Sulfonic acid-related Δm's (n = 8, 0 – 60% matched CHOS precursor ions in both samples), alkylthiol/ thiol-related Δm's (n = 3, 0 – 36% matched precursor ions), and thioether-related Δm's (n = 6, 0 - 44% matched precursor ions, **Table S-20**). This finding was in line with a proposed wider structural diversity (but not necessarily number) of terrestrial CHOS compounds compared to deep-sea DOM.[40,79]

Supplementary Material References

(1) Simon, C.; Dührkop, K.; Petras, D.; Roth, V.-N.; Böcker, S.; Dorrestein, P. C.; Gleixner, G. Structural Insight into Conifer Forest Topsoil and Blackwater Dissolved Organic Matter by Orbitrap Tandem MS Mass Difference. *PANGAEA Data Arch. Publ.* **2022**. https://doi.org/10.1594/PANGAEA.944673.

(2) Ncube, E. N.; Mhlongo, M. I.; Piater, L. A.; Steenkamp, P. A.; Dubery, I. A.; Madala, N. E. Analyses of Chlorogenic Acids and Related Cinnamic Acid Derivatives from Nicotiana Tabacum Tissues with the Aid of UPLC-QTOF-MS/MS Based on the in-Source Collision-Induced Dissociation Method. *Chem. Cent. J.* **2014**, *8* (1), 1–10. https://doi.org/10.1186/s13065-014-0066-z.

(3) Mullen, W.; Yokota, T.; Lean, M. E. J.; Crozier, A. Analysis of Ellagitannins and Conjugates of Ellagic Acid and Quercetin in Raspberry Fruits by LC-MSn. *Phytochemistry* **2003**, *64* (2), 617–624. https://doi.org/10.1016/S0031-9422(03)00281-4.

(4) Fischer, U. A.; Carle, R.; Kammerer, D. R. Identification and Quantification of Phenolic Compounds from Pomegranate (Punica Granatum L.) Peel, Mesocarp, Aril and Differently Produced Juices by HPLC-DAD-ESI/MSn. *Food Chem.* **2011**, *127* (2), 807–821. https://doi.org/10.1016/j.foodchem.2010.12.156.

(5) Engström, M. T.; Pälijärvi, M.; Salminen, J. P. Rapid Fingerprint Analysis of Plant Extracts for Ellagitannins, Gallic Acid, and Quinic Acid Derivatives and Quercetin-, Kaempferol- and Myricetin-Based Flavonol Glycosides by UPLC-QqQ-MS/MS. *J. Agric. Food Chem.* **2015**, *63* (16), 4068–4079. https://doi.org/10.1021/acs.jafc.5b00595.

(6) Wyrepkowski, C. C.; Da Costa, D. L. M. G.; Sinhorin, A. P.; Vilegas, W.; De Grandis, R. A.; Resende, F. A.; Varanda, E. A.; Dos Santos, L. C. Characterization and Quantification of the Compounds of the Ethanolic Extract from Caesalpinia Ferrea Stem Bark and Evaluation of Their Mutagenic Activity. *Molecules* **2014**, *19* (10), 16039–16057. https://doi.org/10.3390/molecules191016039.

(7) Rockenbach, I. I.; Jungfer, E.; Ritter, C.; Santiago-Schübel, B.; Thiele, B.; Fett, R.; Galensa, R. Characterization of Flavan-3-Ols in Seeds of Grape Pomace by CE, HPLC-DAD-MS n and LC-ESI-FTICR-MS. *Food Res. Int.* **2012**, *48* (2), 848–855. https://doi.org/10.1016/j.foodres.2012.07.001.

(8) Gu, L.; Kelm, M. A.; Hammerstone, J. F.; Beecher, G.; Holden, J.; Haytowitz, D.; Prior, R. L. Screening of Foods Containing Proanthocyanidins and Their Structural Characterization Using LC-MS/MS and Thiolytic Degradation. *J. Agric. Food Chem.* **2003**, *51* (25), 7513–7521. https://doi.org/10.1021/jf034815d.

(9) Miketova, P.; Schram, K. H.; Whitney, J.; Li, M.; Huang, R.; Kerns, E.; Valcic, S.; Timmermann, B. N.; Rourick, R.; Klohr, S. Tandem Mass Spectrometry Studies of Green Tea Catechins. Identification of Three Minor Components in the Polyphenolic Extract of Green Tea. *J. Mass Spectrom.* **2000**, *35* (7), 860–869. https://doi.org/10.1002/1096-9888(200007)35:7<860::AID-JMS10>3.0.CO;2-J.

(10) Yuzuak, S.; Ballington, J.; Xie, D.-Y. HPLC-QTOF-MS/MS-Based Profiling of Flavan-3-Ols and Dimeric Proanthocyanidins in Berries of Two Muscadine Grape Hybrids FLH 13-11 and FLH 17-

66. *Metabolites* **2018**, *8* (4), 57. https://doi.org/10.3390/metabo8040057.

(11) Fabre, N.; Rustan, I.; De Hoffmann, E.; Quetin-Leclercq, J. Determination of Flavone, Flavonol, and Flavanone Aglycones by Negative Ion Liquid Chromatography Electrospray Ion Trap Mass Spectrometry. *J. Am. Soc. Mass Spectrom.* **2001**, *12* (6), 707–715. https://doi.org/10.1016/S1044-0305(01)00226-4.

(12) Saldanha, L. L.; Vilegas, W.; Dokkedal, A. L. Characterization of Flavonoids and Phenolic Acids in Myrcia Bella Cambess. Using FIA-ESI-IT-MSn and HPLC-PAD-ESI-IT-MS Combined with NMR. *Molecules* **2013**, *18* (7), 8402–8416. https://doi.org/10.3390/molecules18078402.

(13) Dührkop, K.; Fleischauer, M.; Ludwig, M.; Aksenov, A. A.; Melnik, A. V.; Meusel, M.; Dorrestein, P. C.; Rousu, J.; Böcker, S. SIRIUS 4: A Rapid Tool for Turning Tandem Mass Spectra into Metabolite Structure Information. *Nat. Methods* **2019**, *16*, 299–302. https://doi.org/10.1038/s41592-019-0344-8.

(14) Dührkop, K.; Shen, H.; Meusel, M.; Rousu, J.; Böcker, S. Searching Molecular Structure Databases with Tandem Mass Spectra Using CSI:FingerID. *Proc. Natl. Acad. Sci.* **2015**, *112* (41), 12580–12585. https://doi.org/10.1073/pnas.1509788112.

(15) Zhang, F.; Harir, M.; Moritz, F.; Zhang, J.; Witting, M.; Wu, Y.; Schmitt-Kopplin, P.; Fekete, A.; Gaspar, A.; Hertkorn, N. Molecular and Structural Characterization of Dissolved Organic Matter during and Post Cyanobacterial Bloom in Taihu by Combination of NMR Spectroscopy and FTICR Mass Spectrometry. *Water Res.* **2014**, *57C*, 280–294. https://doi.org/10.1016/j.watres.2014.02.051.

(16) Longnecker, K.; Kujawinski, E. B. Using Network Analysis to Discern Compositional Patterns in Ultrahigh-Resolution Mass Spectrometry Data of Dissolved Organic Matter. *Rapid Commun. Mass Spectrom.* **2016**, *30* (22), 2388–2394. https://doi.org/10.1002/rcm.7719.

(17) Cortés-Francisco, N.; Caixach, J. Fragmentation Studies for the Structural Characterization of Marine Dissolved Organic Matter. *Anal. Bioanal. Chem.* **2015**, *407*, 2455–2462. https://doi.org/10.1007/s00216-015-8499-3.

(18) Kunenkov, E. V.; Kononikhin, A. S.; Perminova, I. V.; Hertkorn, N.; Gaspar, A.; Schmitt-kopplin, P.; Popov, I. A.; Garmash, A. V.; Nikolaev, E. N. Total Mass Difference Statistics Algorithm : A New Approach to Identification of High-Mass Building Blocks in Electrospray Ionization Fourier Transform Ion Cyclotron Mass Spectrometry Data of Natural Organic Matter. *Anal. Chem.* **2009**, *81* (24), 10106–10115. https://doi.org/10.1021/ac901476u.

(19) Kujawinski, E. B.; Behn, M. D. Automated Analysis of Electrospray Ionization Fourier Transform Ion Cyclotron Resonance Mass Spectra of Natural Organic Matter. *Anal. Chem.* **2006**, *78* (13), 4363–4373. https://doi.org/10.1021/ac0600306.

(20) Witt, M.; Fuchser, J.; Koch, B. P. Fragmentation Studies of Fulvic Acids Using Collision Induced Dissociation Fourier Transform Ion Cyclotron Resonance Mass Spectrometry. *Anal. Chem.* **2009**, *81* (7), 2688–2694. https://doi.org/10.1021/ac802624s.

(21) Osterholz, H.; Niggemann, J.; Giebel, H.-A.; Simon, M.; Dittmar, T. Inefficient Microbial Production of Refractory Dissolved Organic Matter in the Ocean. *Nat. Commun.* **2015**, *6* (May), 7422. https://doi.org/10.1038/ncomms8422.

(22) Hawkes, J. A.; Patriarca, C.; Sjöberg, P. J. R.; Tranvik, L. J.; Bergquist, J. Extreme Isomeric Complexity of Dissolved Organic Matter Found across Aquatic Environments. *Limnol. Oceanogr. Lett.* **2018**, *3* (2), 21–30. https://doi.org/10.1002/lol2.10064.

(23) Pohlabeln, A. M.; Dittmar, T. Novel Insights into the Molecular Structure of Non-Volatile Marine Dissolved Organic Sulfur. *Mar. Chem.* **2015**, *168*, 86–94. https://doi.org/10.1016/j.marchem.2014.10.018.

(24) Boye, K.; Noël, V.; Tfaily, M. M.; Bone, S. E.; Williams, K. H.; Bargar, J. R.; Fendorf, S. Thermodynamically Controlled Preservation of Organic Carbon in Floodplains. *Nat. Geosci.* **2017**, *10* (6), 415–419. https://doi.org/10.1038/ngeo2940.

(25) Herzsprung, P.; Hertkorn, N.; von Tümpling, W.; Harir, M.; Friese, K.; Schmitt-Kopplin, P. Understanding Molecular Formula Assignment of Fourier Transform Ion Cyclotron Resonance Mass Spectrometry Data of Natural Organic Matter from a Chemical Point of View. *Anal. Bioanal. Chem.* **2014**, *406* (30), 7977–7987. https://doi.org/10.1007/s00216-014-8249-y.

(26) Koch, B. P.; Dittmar, T. From Mass to Structure: An Aromaticity Index for High-Resolution Mass Data of Natural Organic Matter. *Rapid Commun. Mass Spectrom.* **2016**, *30* (1), 250. https://doi.org/10.1002/rcm.7433.

(27) Minor, E. C.; Swenson, M. M.; Mattson, B. M.; Oyler, A. R. Structural Characterization of Dissolved Organic Matter: A Review of Current Techniques for Isolation and Analysis. *Environ. Sci. Process. Impacts* **2014**, *16*, 2064–2079. https://doi.org/10.1039/C4EM00062E.

(28) Hawkes, J. A.; D'Andrilli, J.; Agar, J. N.; Barrow, M. P.; Berg, S. M.; Catalán, N.; Chen, H.; Chu, R. K.; Cole, R. B.; Dittmar, T.; Gavard, R.; Gleixner, G.; Hatcher, P. G.; He, C.; Hess, N. J.; Hutchins, R. H. S.; Ijaz, A.; Jones, H. E.; Kew, W.; Khaksari, M.; Lozano, D. C. P.; Lv, J.; Mazzoleni, L.; Noriega-Ortega, B.; Osterholz, H.; Radoman, N.; Remucal, C. K.; Schmitt, N. D.; Schum, S.; Shi, Q.; Simon, C.; Singer, G.; Sleighter, R. S.; Stubbins, A.; Thomas, M. J.; Tolic, N.; Zhang, S.; Zito, P.; Podgorski, D. C. An International Laboratory Comparison of Dissolved Organic Matter Composition by High Resolution Mass Spectrometry: Are We Getting the Same Answer? *Limnol. Oceanogr. Methods* **2020**, *18*, 235–258.

(29) Djoumbou Feunang, Y.; Eisner, R.; Knox, C.; Chepelev, L.; Hastings, J.; Owen, G.; Fahy, E.; Steinbeck, C.; Subramanian, S.; Bolton, E.; Greiner, R.; Wishart, D. S. ClassyFire: Automated Chemical Classification with a Comprehensive, Computable Taxonomy. *J. Cheminform.* **2016**, *8*, 61. https://doi.org/10.1186/s13321-016-0174-y.

(30) Horai, H.; Arita, M.; Kanaya, S.; Nihei, Y.; Ikeda, T.; Suwa, K.; Ojima, Y.; Tanaka, K.; Tanaka, S.; Aoshima, K.; Oda, Y.; Kakazu, Y.; Kusano, M.; Tohge, T.; Matsuda, F.; Sawada, Y.; Hirai, M. Y.; Nakanishi, H.; Ikeda, K.; Akimoto, N.; Maoka, T.; Takahashi, H.; Ara, T.; Sakurai, N.; Suzuki, H.; Shibata, D.; Neumann, S.; Iida, T.; Tanaka, K.; Funatsu, K.; Matsuura, F.; Soga, T.; Taguchi, R.; Saito, K.; Nishioka, T. MassBank: A Public Repository for Sharing Mass Spectral Data for Life Sciences. *J. Mass Spectrom.* **2010**, *45*, 703–714. https://doi.org/10.1002/jms.1777.

(31) Wang, M.; Carver, J. J.; Phelan, V. V.; Sanchez, L. M.; Garg, N.; Peng, Y.; Nguyen, D. T. D. D.; Watrous, J.; Kapono, C. A.; Luzzatto-Knaan, T.; Porto, C.; Bouslimani, A.; Melnik, A. V.; Meehan, M. J.; Liu, W. T.; Crüsemann, M.; Boudreau, P. D.; Esquenazi, E.; Sandoval-Calderón, M.; Kersten, R. D.; Pace, L. A.; Quinn, R. A.; Duncan, K. R.; Hsu, C. C.; Floros, D. J.; Gavilan, R. G.; Kleigrewe, K.; Northen, T.; Dutton, R. J.; Parrot, D.; Carlson, E. E.; Aigle, B.; Michelsen,

C. F.; Jelsbak, L.; Sohlenkamp, C.; Pevzner, P.; Edlund, A.; McLean, J.; Piel, J.; Murphy, B. T.; Gerwick, L.; Liaw, C. C.; Yang, Y. L.; Humpf, H. U.; Maansson, M.; Keyzers, R. A.; Sims, A. C.; Johnson, A. R.; Sidebottom, A. M.; Sedio, B. E.; Klitgaard, A.; Larson, C. B.; Boya, C. A. P.; Torres-Mendoza, D.; Gonzalez, D. J.; Silva, D. B.; Marques, L. M.; Demarque, D. P.; Pociute, E.; O'Neill, E. C.; Briand, E.; Helfrich, E. J. N.; Granatosky, E. A.; Glukhov, E.; Ryffel, F.; Houson, H.; Mohimani, H.; Kharbush, J. J.; Zeng, Y.; Vorholt, J. A.; Kurita, K. L.; Charusanti, P.; McPhail, K. L.; Nielsen, K. F.; Vuong, L.; Elfeki, M.; Traxler, M. F.; Engene, N.; Koyama, N.; Vining, O. B.; Baric, R.; Silva, R. R.; Mascuch, S. J.; Tomasi, S.; Jenkins, S.; Macherla, V.; Hoffman, T.; Agarwal, V.; Williams, P. G.; Dai, J.; Neupane, R.; Gurr, J.; Rodríguez, A. M. C.; Lamsa, A.; Zhang, C.; Dorrestein, K.; Duggan, B. M.; Almaliti, J.; Allard, P. M.; Phapale, P.; Nothias, L. F.; Alexandrov, T.; Litaudon, M.; Wolfender, J. L.; Kyle, J. E.; Metz, T. O.; Peryea, T.; Nguyen, D. T. D. D.; VanLeer, D.; Shinn, P.; Jadhav, A.; Müller, R.; Waters, K. M.; Shi, W.; Liu, X.; Zhang, L.; Knight, R.; Jensen, P. R.; Palsson, B.; Pogliano, K.; Linington, R. G.; Gutiérrez, M.; Lopes, N. P.; Gerwick, W. H.; Moore, B. S.; Dorrestein, P. C.; Bandeira, N. Sharing and Community Curation of Mass Spectrometry Data with Global Natural Products Social Molecular Networking. *Nat. Biotechnol.* **2016**, *34*, 828–837. https://doi.org/10.1038/nbt.3597.

(32) Laszakovits, J. R.; MacKay, A. A. Data-Based Chemical Class Regions for Van Krevelen Diagrams. *J. Am. Soc. Mass Spectrom.* **2022**, *33*, 198–202. https://doi.org/https://doi.org/10.1021/jasms.1c00230.

(33) Chassagne, F.; Cabanac, G.; Hubert, G.; David, B.; Marti, G. The Landscape of Natural Product Diversity and Their Pharmacological Relevance from a Focus on the Dictionary of Natural Products®. *Phytochem. Rev.* **2019**, 1–22. https://doi.org/10.1007/s11101-019-09606-2.

(34) Nakamura, Y.; Mochamad Afendi, F.; Kawsar Parvin, A.; Ono, N.; Tanaka, K.; Hirai Morita, A.; Sato, T.; Sugiura, T.; Altaf-Ul-Amin, M.; Kanaya, S. KNApSAcK Metabolite Activity Database for Retrieving the Relationships between Metabolites and Biological Activities. *Plant Cell Physiol.* **2014**, *55*, e7. https://doi.org/10.1093/pcp/pct176.

(35) Caspi, R.; Billington, R.; Keseler, I. M.; Kothari, A.; Krummenacker, M.; Midford, P. E.; Ong, W. K.; Paley, S.; Subhraveti, P.; Karp, P. D. The MetaCyc Database of Metabolic Pathways and Enzymes - a 2019 Update. *Nucleic Acids Res.* **2019**, *48*, D455–D453. https://doi.org/10.1093/nar/gkz862.

(36) Okuda, S.; Yamada, T.; Hamajima, M.; Itoh, M.; Katayama, T.; Bork, P.; Goto, S.; Kanehisa, M. KEGG Atlas Mapping for Global Analysis of Metabolic Pathways. *Nucleic Acids Res.* **2008**, *36*, 423–426. https://doi.org/10.1093/nar/gkn282.

(37) Wishart, D. S.; Tzur, D.; Knox, C.; Eisner, R.; Guo, A. C.; Young, N.; Cheng, D.; Jewell, K.; Arndt, D.; Sawhney, S.; Fung, C.; Nikolai, L.; Lewis, M.; Coutouly, M. A.; Forsythe, I.; Tang, P.; Shrivastava, S.; Jeroncic, K.; Stothard, P.; Amegbey, G.; Block, D.; Hau, D. D.; Wagner, J.; Miniaci, J.; Clements, M.; Gebremedhin, M.; Guo, N.; Zhang, Y.; Duggan, G. E.; MacInnis, G. D.; Weljie, A. M.; Dowlatabadi, R.; Bamforth, F.; Clive, D.; Greiner, R.; Li, L.; Marrie, T.; Sykes, B. D.; Vogel, H. J.; Querengesser, L. HMDB: The Human Metabolome Database. *Nucleic Acids Res.* **2007**, *35*, 521–526. https://doi.org/10.1093/nar/gkl923.

(38) Jeffryes, J. G.; Colastani, R. L.; Elbadawi-Sidhu, M.; Kind, T.; Niehaus, T. D.; Broadbelt, L. J.; Hanson, A. D.; Fiehn, O.; Tyo, K. E. J.; Henry, C. S. MINEs: Open Access Databases of Computationally Predicted Enzyme Promiscuity Products for Untargeted Metabolomics. *J. Cheminform.* **2015**, *7*, 44. https://doi.org/10.1186/s13321-015-0087-1.

(39) Brown, T. A.; Jackson, B. A.; Bythell, B. J.; Stenson, A. C. Benefits of Multidimensional Fractionation for the Study and Characterization of Natural Organic Matter. *J. Chromatogr. A* **2016**, *1470*, 84–96. https://doi.org/10.1016/j.chroma.2016.10.005.

(40) Simon, C.; Roth, V.-N.; Dittmar, T.; Gleixner, G. Molecular Signals of Heterogeneous Terrestrial Environments Identified in Dissolved Organic Matter: A Comparative Analysis of Orbitrap and Ion Cyclotron Resonance Mass Spectrometers. *Front. Earth Sci.* **2018**, *6*, 1–16. https://doi.org/10.3389/feart.2018.00138.

(41) Merder, J.; Freund, J. A.; Feudel, U.; Hansen, C. T.; Hawkes, J. A.; Jacob, B.; Klaproth, K.; Niggemann, J.; Noriega-Ortega, B. E.; Osterholz, H.; Rossel, P. E.; Seidel, M.; Singer, G.; Stubbins, A.; Waska, H.; Dittmar, T. ICBM-OCEAN: Processing Ultrahigh-Resolution Mass Spectrometry Data of Complex Molecular Mixtures. *Anal. Chem.* **2020**, *92*, 6832–6838. https://doi.org/10.1021/acs.analchem.9b05659.

(42) Zark, M.; Dittmar, T. Universal Molecular Structures in Natural Dissolved Organic Matter. *Nat. Commun.* **2018**, *9* (1), 3178. https://doi.org/10.1038/s41467-018-05665-9.

(43) Chambers, M. C.; Maclean, B.; Burke, R.; Amodei, D.; Ruderman, D. L.; Neumann, S.; Gatto, L.; Fischer, B.; Pratt, B.; Egertson, J.; Hoff, K.; Kessner, D.; Tasman, N.; Shulman, N.; Frewen, B.; Baker, T. a; Brusniak, M.-Y.; Paulse, C.; Creasy, D.; Flashner, L.; Kani, K.; Moulding, C.; Seymour, S. L.; Nuwaysir, L. M.; Lefebvre, B.; Kuhlmann, F.; Roark, J.; Rainer, P.; Detlev, S.; Hemenway, T.; Huhmer, A.; Langridge, J.; Connolly, B.; Chadick, T.; Holly, K.; Eckels, J.; Deutsch, E. W.; Moritz, R. L.; Katz, J. E.; Agus, D. B.; MacCoss, M.; Tabb, D. L.; Mallick, P. A Cross-Platform Toolkit for Mass Spectrometry and Proteomics. *Nat. Biotechnol.* **2012**, *30*, 918–920. https://doi.org/10.1038/nbt.2377.

(44) Strohalm, M.; Kavan, D.; Novák, P.; Volný, M.; Havlíček, V. MMass 3: A Cross-Platform Software Environment for Precise Analysis of Mass Spectrometric Data. *Anal. Chem.* **2010**, *82*, 4648–4651. https://doi.org/10.1021/ac100818g.

(45) Roth, V.-N.; Dittmar, T.; Gaupp, R.; Gleixner, G. Ecosystem-Specific Composition of Dissolved Organic Matter. *Vadose Zo. J.* **2014**, *13*. https://doi.org/http://dx.doi.org/10.2136/vzj2013.09.0162.

(46) Green, N. W.; Mcinnis, D.; Hertkorn, N.; Maurice, P. A.; Perdue, M. E. Suwannee River Natural Organic Matter : Isolation of the 2R101N Reference Sample by Reverse Osmosis. *Environ. Eng. Sci.* **2014**, *32*, 38–44. https://doi.org/10.1089/ees.2014.0284.

(47) Kindler, R.; Siemens, J.; Kaiser, K.; Walmsley, D. C.; Bernhofer, C.; Buchmann, N.; Cellier, P.; Lehuger, S.; Jones, S. K.; Skiba, U.; Eugster, W.; Ibrom, A.; Kutsch, W.; Osborne, B.; Soussana, J.-F.; Tefs, C.; Moors, E.; Heim, A.; Saunders, M.; Jones, M.; Grünwald, T.; Gleixner, G.; Loubet, B.; McKenzie, R.; Pilegaard, K.; Schmidt, M. W. I.; Zeeman, M. J.; Seyfferth, J.; Larsen, K. S.; Vowinckel, B.; Klumpp, K.; Schrumpf, M.; Rebmann, C.; Sutton, M. A.; Kaupenjohann, M. Dissolved Carbon Leaching from Soil Is a Crucial Component of the Net Ecosystem Carbon Balance. *Glob. Chang. Biol.* **2010**, *17* (2), 1167–1185. https://doi.org/10.1111/j.1365-2486.2010.02282.x.

(48) Roth, V.-N.; Dittmar, T.; Gaupp, R.; Gleixner, G. The Molecular Composition of Dissolved Organic Matter in Forest Soils as a Function of PH and Temperature. *PLoS One* **2015**, *10*, e0119188. https://doi.org/10.1371/journal.pone.0119188.

(49) Dittmar, T.; Koch, B.; Hertkorn, N.; Kattner, G. A Simple and Efficient Method for the Solid-Phase Extraction of Dissolved Organic Matter (SPE-DOM) from Seawater. *Limnol. Oceanogr. Methods* **2008**, *6*, 230–235. https://doi.org/10.4319/lom.2008.6.230.

(50) Riedel, T.; Dittmar, T. A Method Detection Limit for the Analysis of Natural Organic Matter via Fourier Transform Ion Cyclotron Resonance Mass Spectrometry. *Anal. Chem.* **2014**, *86*, 8376–8382. https://doi.org/10.1021/ac501946m.

(51) Wagner, S.; Dittmar, T.; Jaffé, R. Molecular Characterization of Dissolved Black Nitrogen via Electrospray Ionization Fourier Transform Ion Cyclotron Resonance Mass Spectrometry. *Org. Geochem.* **2015**, *79*, 21–30. https://doi.org/10.1016/j.orggeochem.2014.12.002.

(52) Smirnov, K. S.; Forcisi, S.; Moritz, F.; Lucio, M.; Schmitt-Kopplin, P. Mass Difference Maps and Their Application for the Re-Calibration of Mass Spectrometric Data in Non-Targeted Metabolomics. *Anal. Chem.* **2019**. https://doi.org/10.1021/acs.analchem.8b04555.

(53) Koch, B. P.; Dittmar, T. From Mass to Structure: An Aromaticity Index for High-Resolution Mass Data of Natural Organic Matter. *Rapid Commun. Mass Spectrom.* **2006**, *30* (1), 250. https://doi.org/10.1002/rcm.2386.

(54) Fisher, R. A. On the Interpretation of X2 from Contingency Tables , and the Calculation of P. *J. R. Stat. Soc.* **1922**, *85*, 87–94.

(55) Rivas-Ubach, A.; Liu, Y.; Bianchi, T. S.; Tolić, N.; Jansson, C.; Paša-Tolić, L. Moving beyond the van Krevelen Diagram: A New Stoichiometric Approach for Compound Classification in Organisms. *Anal. Chem.* **2018**, *90*, 6152–6160. https://doi.org/10.1021/acs.analchem.8b00529.

(56) Hammer, Ø.; Harper, D. A.; Ryan, P. D. PAST: Paleontological Statistics Software Package for Education and Data Analysis. *Palaeontol. Electron.* **2001**, *4*, 9.

(57) Zark, M.; Christoffers, J.; Dittmar, T. Molecular Properties of Deep-Sea Dissolved Organic Matter Are Predictable by the Central Limit Theorem: Evidence from Tandem FT-ICR-MS. *Mar. Chem.* **2017**, *191*, 9–15. https://doi.org/10.1016/j.marchem.2017.02.005.

(58) Capley, E. N.; Tipton, J. D.; Marshall, A. G.; Stenson, A. C. Chromatographic Reduction of Isobaric and Isomeric Complexity of Fulvic Acids to Enable Multistage Tandem Mass Spectral Characterization. *Anal. Chem.* **2010**, *82* (19), 8194–8202. https://doi.org/10.1021/ac1016216.

(59) These, A.; Winkler, M.; Thomas, C.; Reemtsma, T. Determination of Molecular Formulas and Structural Regularities of Low Molecular Weight Fulvic Acids by Size-Exclusion Chromatography with Electrospray Ionization Quadrupole Time-of-Flight Mass Spectrometry. *Rapid Commun. Mass Spectrom.* **2004**, *18* (16), 1777–1786. https://doi.org/10.1002/rcm.1550.

(60) Reemtsma, T. The Carbon versus Mass Diagram to Visualize and Exploit FTICR-MS Data of Natural Organic Matter. *J. Mass Spectrom.* **2010**, *45* (4), 382–390. https://doi.org/10.1002/jms.1722.

(61) Lee, J. H.; Johnson, J. V.; Talcott, S. T. Identification of Ellagic Acid Conjugates and Other Polyphenolics in Muscadine Grapes by HPLC-ESI-MS. *J. Agric. Food Chem.* **2005**, *53* (15), 6003–6010. https://doi.org/10.1021/jf050468r.

(62) Poon, G. K. Analysis of Catechins in Tea Extracts by Liquid Chromatography-Electrospray Ionization Mass Spectrometry. *J. Chromatogr. A* **1998**, *794* (1–2), 63–74. https://doi.org/10.1016/S0021-9673(97)01050-9.

(63) Galaverna, R. S.; Sampaio, P. T. B.; Barata, L. E. S.; Eberlin, M. N.; Fidelis, C. H. V. Differentiation of Two Morphologically Similar Amazonian Aniba Species by Mass Spectrometry Leaf Fingerprinting. *Anal. Methods* **2015**, *7* (5), 1984–1990. https://doi.org/10.1039/c4ay02598a.

(64) Stöggl, W. M.; Huck, C. W.; Bonn, G. K. Structural Elucidation of Catechin and Epicatechin in Sorrel Leaf Extracts Using Liquid-Chromatography Coupled to Diode Array-, Fluorescence- ,and Mass Spectrometric Detection. *J. Sep. Sci.* **2004**, *27* (7–8), 524–528. https://doi.org/10.1002/jssc.200301694.

(65) da Costa, M. F.; Galaverna, R. S.; Pudenzi, M. A.; Ruiz, A. L. T. G.; de Carvalho, J. E.; Eberlin, M. N.; dos Santos, C. Profiles of Phenolic Compounds by FT-ICR MS and Antioxidative and Antiproliferative Activities of Stryphnodendron Obovatum Benth Leaf Extracts. *Anal. Methods* **2016**, *8* (31), 6056–6063. https://doi.org/10.1039/C6AY01272H.

(66) Nimmagadda, R. D.; McRae, C. Characterisation of the Backbone Structures of Several Fulvic Acids Using a Novel Selective Chemical Reduction Method. *Org. Geochem.* **2007**, *38* (7), 1061–1072. https://doi.org/10.1016/j.orggeochem.2007.02.016.

(67) Perdue, E. M.; Hertkorn, N.; Kettrup, A. Substitution Patterns in Aromatic Rings by Increment Analysis. Model Development and Application to Natural Organic Matter. *Anal. Chem.* **2007**, *79* (3), 1010–1021. https://doi.org/10.1021/ac061611y.

(68) Zubarev, R. A.; Makarov, A. Orbitrap Mass Spectrometry. *Anal. Chem.* **2013**, *85*, 5288–5296. https://doi.org/10.1021/ac4001223.

(69) Schymanski, E. L.; Singer, H. P.; Slobodnik, J.; Ipolyi, I. M.; Oswald, P.; Krauss, M.; Schulze, T.; Haglund, P.; Letzel, T.; Grosse, S.; Thomaidis, N. S.; Bletsou, A.; Zwiener, C.; Ibáñez, M.; Portolés, T.; De Boer, R.; Reid, M. J.; Onghena, M.; Kunkel, U.; Schulz, W.; Guillon, A.; Noyon, N.; Leroy, G.; Bados, P.; Bogialli, S.; Stipaničev, D.; Rostkowski, P.; Hollender, J. Non-Target Screening with High-Resolution Mass Spectrometry: Critical Review Using a Collaborative Trial on Water Analysis. *Anal. Bioanal. Chem.* **2015**, *407*, 6237–6255. https://doi.org/10.1007/s00216-015-8681-7.

(70) Hollender, J.; Schymanski, E. L.; Singer, H. P.; Ferguson, P. L. Nontarget Screening with High Resolution Mass Spectrometry in the Environment: Ready to Go? *Environ. Sci. Technol.* **2017**, *51*, 11505–11512. https://doi.org/10.1021/acs.est.7b02184.

(71) Luek, J. L.; Schmitt-kopplin, P.; Mouser, P. J.; Petty, W. T.; Richardson, S. D.; Gonsior, M. Halogenated Organic Compounds Identified in Hydraulic Fracturing Wastewaters Using Ultrahigh Resolution Mass Spectrometry. *Environ. Sci. Technol.* **2017**, *51*, 5377–5385. https://doi.org/10.1021/acs.est.6b06213.

(72) Liu, Z.; Sleighter, R. L.; Zhong, J.; Hatcher, P. G. The Chemical Changes of DOM from Black Waters to Coastal Marine Waters by HPLC Combined with Ultrahigh Resolution Mass Spectrometry. *Estuar. Coast. Shelf Sci.* **2011**, *92*, 205–216. https://doi.org/10.1016/j.ecss.2010.12.030.

(73) Flerus, R.; Koch, B. P.; Schmitt-Kopplin, P.; Witt, M.; Kattner, G. Molecular Level Investigation of Reactions between Dissolved Organic Matter and Extraction Solvents Using FT-ICR MS. *Mar. Chem.* **2011**, *124*, 100–107. https://doi.org/10.1016/j.marchem.2010.12.006.

(74) Benk, S. A.; Li, Y.; Roth, V.-N.; Gleixner, G. Lignin Dimers as Potential Markers for 14C-Young Terrestrial Dissolved Organic Matter in the Critical Zone. *Front. Earth Sci.* **2018**, 1–9. https://doi.org/10.3389/feart.2018.00168.

(75) Reemtsma, T.; These, A.; Linscheid, M.; Leenheer, J.; Spitzy, A. Molecular and Structural Characterization of Dissolved Organic Matter from the Deep Ocean by FTICR-MS, Including Hydrophilic Nitrogenous Organic Molecules. *Environ. Sci. Technol.* **2008**, *42*, 1430–1437. https://doi.org/10.1021/es7021413.

(76) Piraud, M.; Vianey-Saban, C.; Petritis, K.; Elfakir, C.; Steghens, J. P.; Morla, A.; Bouchu, D. ESI-MS/MS Analysis of Underivatised Amino Acids: A New Tool for the Diagnosis of Inherited Disorders of Amino Acid Metabolism. Fragmentation Study of 79 Molecules of Biological Interest in Positive and Negative Ionisation Mode. *Rapid Commun. Mass Spectrom.* **2003**, *17*, 1297–1311. https://doi.org/10.1002/rcm.1054.

(77) Lemr, K.; Holčapek, M.; Jandera, P.; Lyka, A. Analysis of Metal Complex Azo Dyes by High-Performance Liquid Chromatography/Electrospray Ionization Mass Spectrometry and Multistage Mass Spectrometry. *Rapid Commun. Mass Spectrom.* **2000**, *14*, 1881–1888.

(78) Liu, L.; Song, C.; Tian, S.; Zhang, Q.; Cai, X.; Liu, Y.; Liu, Z.; Wang, W. Structural Characterization of Sulfur-Containing Aromatic Compounds in Heavy Oils by FT-ICR Mass Spectrometry with a Narrow Isolation Window. *Fuel* **2019**, *240*, 40–48. https://doi.org/10.1016/j.fuel.2018.11.130.

(79) Poulin, B. A.; Ryan, J. N.; Nagy, K. L.; Stubbins, A.; Dittmar, T.; Orem, W.; Krabbenhoft, D. P.; Aiken, G. R. Spatial Dependence of Reduced Sulfur in Everglades Dissolved Organic Matter Controlled by Sulfate Enrichment. *Environ. Sci. Technol.* **2017**, *51*, 3630–3639. https://doi.org/10.1021/acs.est.6b04142.
